# Enhanced‐Type Quantitative Luminescence Recognition for Per‐ and Polyfluoroalkyl Substances (PFAS) by a Metal–Organic Framework Single Crystal

**DOI:** 10.1002/anie.202515775

**Published:** 2025-11-24

**Authors:** Zongsu Han, Yifan Guo, Kun‐Yu Wang, Wenxuan Li, Jiatong Huo, Qingya Huang, Vladimir I. Bakhmutov, Yihao Yang, Rong‐Ran Liang, Peter R. Taylor, Wei Shi, Hong‐Cai Zhou

**Affiliations:** ^1^ Department of Chemistry Texas A&M University College Station TX 77843 USA; ^2^ School of Pharmaceutical Science and Technology Tianjin University Tianjin 300072 China; ^3^ Frontiers Science Center for New Organic Matter State Key Laboratory of Elemento‐Organic Chemistry and Department of Chemistry College of Chemistry Nankai University Tianjin 300071 China; ^4^ Department of Chemistry Princeton University Princeton NJ 08544 USA; ^5^ Department of Materials Science and Engineering University of Pennsylvania Philadelphia PA 19104 USA

**Keywords:** Enhanced luminescence, Metal–organic framework, PFAS detecting, Single crystal sensor

## Abstract

Rapid and quantitative detection of per‐ and polyfluoroalkyl substances (PFAS) remains a critical challenge in environmental monitoring due to their low light absorption capacities. Luminescent sensing based on metal–organic frameworks (MOFs) enables analyte‐specific optical responses through well‐defined host–guest interactions, though conventional MOF powders as sensing materials face limitations in practical deployment for their stabilities and recyclable capacities. Herein, we report a millimeter‐sized luminescent MOF single crystal that functions as a reusable sensor for PFAS, exhibiting an enhanced‐type luminescence response upon analyte binding. In contrast to MOF powders, which often suffer from suspension instability and material loss during recycling, the single‐crystal format offers robust structural integrity, direct handling, and facile recovery. The sensor exhibits exponential luminescence responses toward five commonly encountered PFAS and retains its sensing performance for 10 cycles. This work presents a durable and scalable luminescent platform for PFAS detection and underscores the role of dimensional control in advancing MOF‐based sensing technologies.

## Graphical abstract

A millimeter‐sized luminescent metal–organic framework (MOF) single crystal is developed as a robust, reusable sensor for per‐ and polyfluoroalkyl substances (PFAS). Unlike conventional sensing conducted by MOF powders, the single crystal enables easy usage, improved stability, and enhanced luminescence response, facilitating practical PFAS detection via well‐defined host–guest interactions.

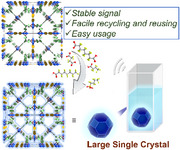

Per‐ and polyfluoroalkyl substances (PFAS) represent a class of synthetic chemicals defined by exceptionally strong carbon─fluorine bonds, imparting extreme resistance to environmental degradation.^[^ ^1^, ^2^, ^3^ ^]^ Widely referred to as “forever chemicals”, PFAS have been used extensively in industrial and consumer applications for their hydrophobic, oleophobic, and thermal stability, leading to their presence in non‐stick cookware, water‐resistant textiles, firefighting foams, and food packaging.^[^ ^4^, ^5^ ^]^ Their environmental persistence and bioaccumulative nature have raised growing concern in recent years.^[^ ^6^, ^7^, ^8^ ^]^ Chronic exposure to certain PFAS compounds is associated with immunotoxicity, developmental toxicity, and elevated cancer risk. The pervasive contamination of soil, water systems, and the food chain has prompted urgent interest in technologies for rapid PFAS detection, efficient removal, and regulatory control.

Quantitative luminescence recognition is a powerful analytical technique that leverages the unique optical properties of luminescent materials to rapidly and precisely detect and quantify specific analytes.^[^ ^9^, ^10^, ^11^ ^]^ Luminescence, the light emitted upon energy absorption, serves as a highly sensitive and non‐invasive method for probing biological, chemical, and physical processes.^[^ ^12^, ^13^, ^14^ ^]^ Quantitative luminescence recognition is typically achieved by monitoring changes in emission intensity (quantum yield), lifetime, or maximum emission wavelength. This approach is widely employed in molecular biology, medical diagnostics, environmental monitoring, and materials science, where it facilitates analysis of analyte–receptor interactions and enables high‐fidelity quantification.^[^ ^15^, ^16^, ^17^, ^18^ ^]^ By offering both sensitivity and selectivity, luminescence‐based sensing has become an essential tool across fundamental studies and real‐world applications.

Metal–organic frameworks (MOFs) have emerged as highly effective luminescence sensors due to their modular structures and tunable chemical compositions.^[^ ^19^, ^20^, ^21^, ^22^, ^23^ ^]^ MOFs are crystalline materials composed of metal nodes coordinated with organic linkers, forming periodic porous frameworks capable of hosting functional guest molecules.^[^ ^24^, ^25^, ^26^ ^]^ Their structure tunability enables precise modulation of luminescent properties, affording high sensitivity and selectivity toward specific analytes.^[^ ^27^, ^28^, ^29^, ^30^ ^]^ Features such as large surface area, diverse pore environments, and customizable photophysical behavior further expand their utility in sensing applications.^[^ ^31^, ^32^, ^33^, ^34^ ^]^ By integrating tailored metal centers, guest molecules, or functional groups, MOFs can be engineered for real‐time, non‐invasive, and highly accurate detection. Traditional luminescent sensing strategies generally follow two pathways, each with inherent benefits and limitations.^[^ ^18^, ^27^, ^35^, ^36^ ^]^ The first pathway uses soluble luminescent molecules, which can form homogeneous solutions and provide stable signals (Figure 1); however, this system faces challenges in recovery and reuse. The second pathway employs insoluble luminescent powders, for example, polymers, MOFs, or covalent organic frameworks (COFs), which will be ground into fine powders first and dispersed in solvents under sonication to create suspensions for sensing. This approach also faces challenges: suspension non‐uniformity leads to sedimentation over time, diminishing luminescence consistency and requiring repeated sonication or stirring before each measurement, and recovery/reuse of powdered materials is cumbersome, often involving time‐intensive centrifugation with substantial sample loss (Figure 1). Such inefficiencies not only reduce reusability but also raise concerns over material waste and environmental impact.

**Figure 1:**
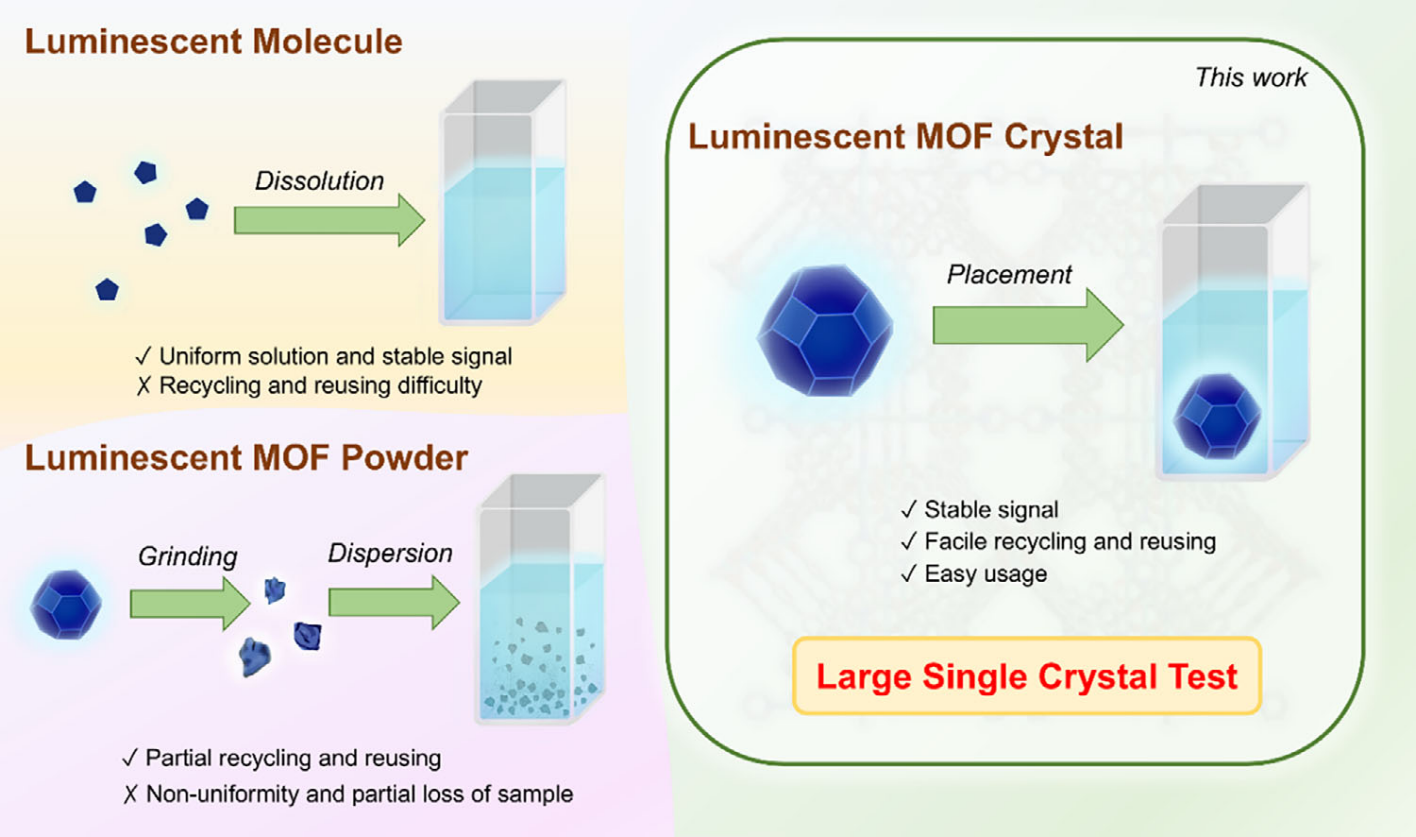
Advantages of MOF single crystal for sensing compared with luminescent molecules and conventional MOF powders.

In this work, a millimeter‐sized luminescent MOF single crystal was synthesized and employed as a sensor (Figure S1) for detecting various PFAS, including perfluorooctanoic acid (PFOA), as well as its analogues tetrafluorosuccinic acid, heptafluorobutyric acid, perfluorobutanesulfonic acid, and perfluorosuberic acid (Figure S2). The MOF single crystals exhibit pronounced luminescence enhancement upon exposure to these PFAS solutions (Figure 2). Compared to conventional powder MOF sensing, the single crystal displays more stable luminescence intensity in solution and offers significantly improved recyclability and ease of reuse. Handling the crystal is considerably more straightforward than preparing powder‐based suspensions, test papers, or membranes. These advantages highlight its potential as a reusable and practical “probe unit” for routine PFAS detection.

**Figure 2:**
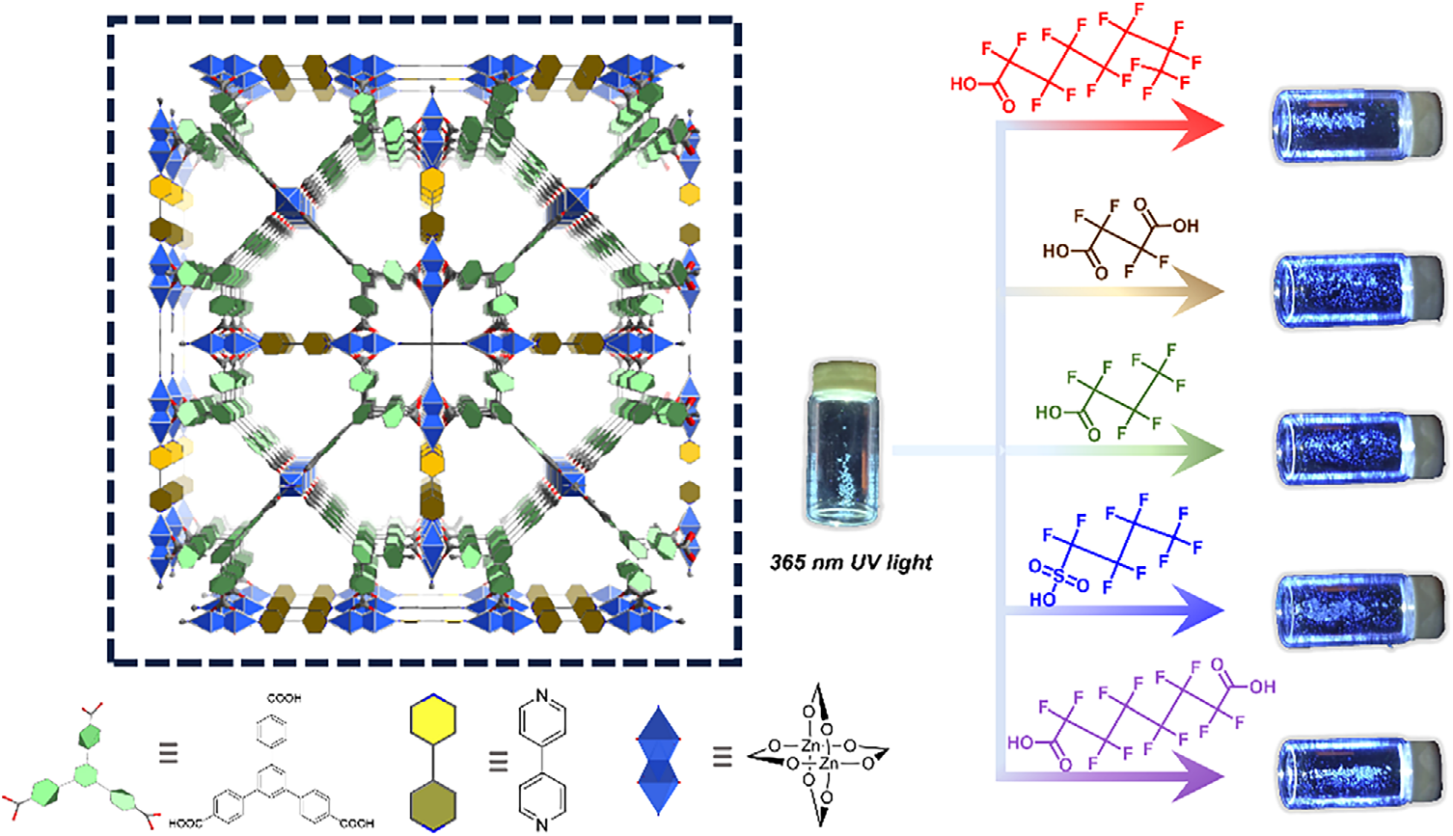
Luminescence enhancement of the single crystals upon exposure to PFAS solutions.

Millimeter‐sized single crystals of ITHD(Zn) were synthesized using Zn(NO_3_)_2_·6H_2_O, benzene‐1,3,5‐tribenzoic acid (H_3_BTB), and 4,4′‐bipyridine (4,4′‐BPY) in *N*,*N*‐dimethylformamide (DMF).^[^ ^37^ ^]^ The cavity has a free diameter of 2.1 nm and is surrounded by 12 pentagonal windows, each with a free diameter of 0.8 nm. Single crystal X‐ray diffraction (SCXRD) and powder X‐ray diffraction (PXRD) results have proven the successful synthesis and the purity of the sample (Figure S3). Thermogravimetric analysis showed continuous mass loss attributed to guest solvents within the pores (Figure S4). PXRD patterns of the ITHD(Zn) with variable temperature and solvents were performed to evaluate the structural stability under potential sensing conditions. ITHD(Zn) remains stable over 120 °C (Figure S5) and in most common solvents (Figure S6).

Excitation and emission spectra of the ITHD(Zn) single crystals were measured and exhibited strong luminescence (Figure S7). The emission spectrum of the ligand H_3_BTB is similar to that of the MOF, with a slightly blue‐shifted maximum peak (Figure S8). Time‐dependent emission spectra of the single crystal remained stable over 10 min, with a relative standard deviation (RSD) of 2.1 × 10^−3^ (Figure S9). For comparison, the single crystals were ground into fine powder, dispersed under long‐time sonication, and measured with and without stirring, showing RSD values of 1.5 × 10^−2^ (Figure S10) and 2.0 × 10^−2^ (Figure S11), respectively. This result confirms the superior emission stability of the single crystal. Besides, no detectable luminescence was observed in the supernatant of the single crystal suspension (Figure S12), indicating that the emission originates from the single crystal itself rather than from decomposition or leached species, further supporting its structural stability in solution.

PFOA, a widely produced perfluorinated carboxylic acid used as an industrial surfactant, has been classified as carcinogenic to humans by the International Agency for Research on Cancer (IARC).^[^ ^1^, ^2^, ^3^ ^]^ A series of PFOA solutions at different concentrations were sequentially added to the cuvette with the MOF single crystal placed at the bottom. Upon PFOA exposure, the emission intensities of ITHD(Zn) single crystal increase obviously (Figure 3). After each measurement, the solution was removed and the single crystal was washed with fresh solvent before the next test (Figure S13), showing great stability in luminescence intensity across cycles (Figure 3b). The emission intensity changes followed an exponential relationship with increasing PFOA concentrations. A series of PFAS analogues were also tested (Figures S14–S17), all of which induced similarly enhanced luminescence responses (Figure 4). The responding efficiencies are further evaluated by Benesi–Hildebrand (B–H) equation with the function as *I*_0_/(*I *− *I*_0_) = K_BH_/([*C*]^n^)+b, where *I*_0_ and *I* are the intensities of the MOF without and with the analytes, [*C*] is the concentration of the analyte, K_BH_ is the association constant and b is a constant^[^ ^18^, ^38^ ^]^ (Figures S18–S22), which show well‐fitted linear behavior with n = 3.

**Figure 3:**
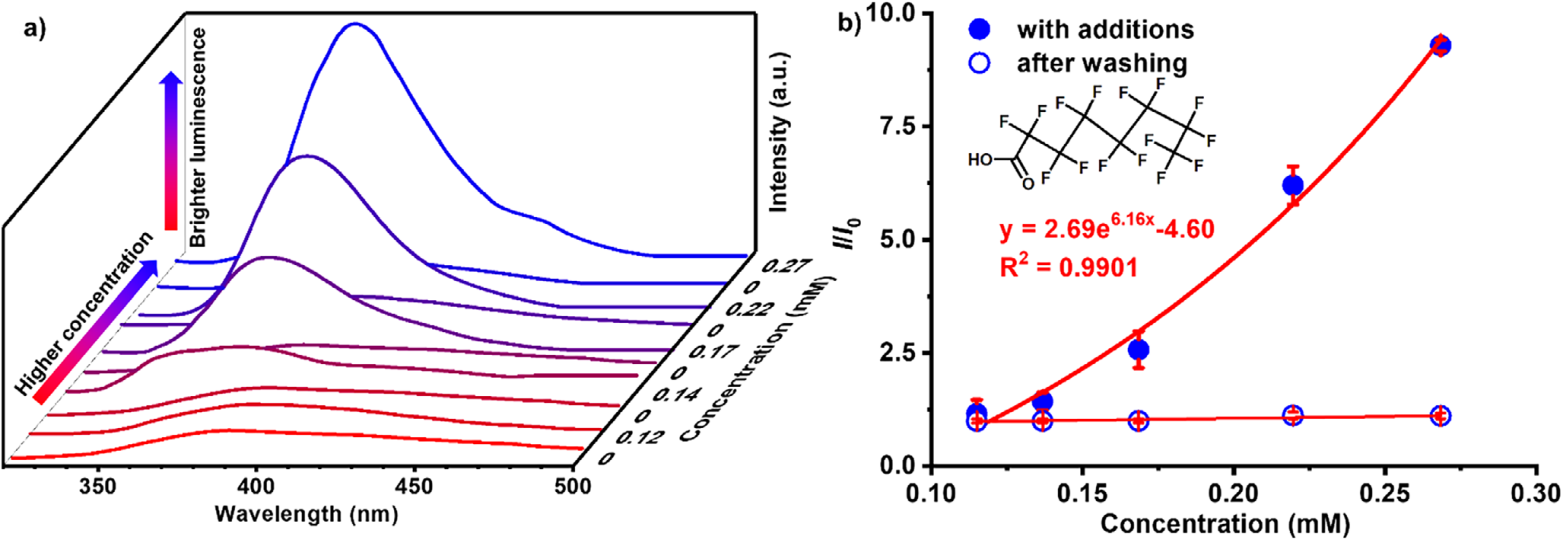
a) Emission spectra of the MOF single crystal with different concentrations of PFOA. b) Emission intensity changes of the MOF single crystal upon repeated exposure to different concentrations of PFOA and washing.

**Figure 4:**
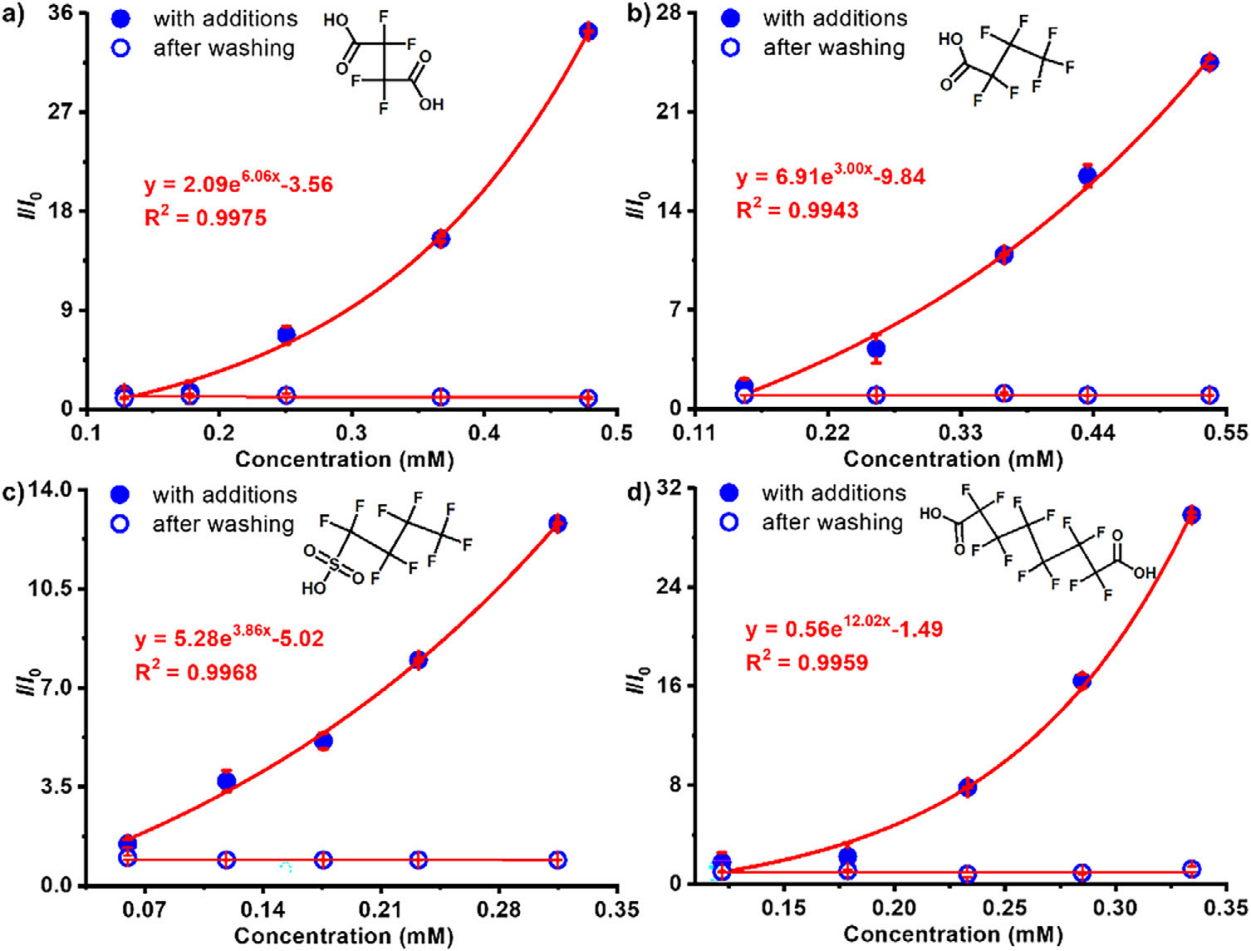
Emission intensity changes of the MOF single crystal in response to different concentrations of tetrafluorosuccinic acid a), heptafluorobutyric acid b), perfluorobutanesulfonic acid c), perfluorosuberic acid d) and after washing.

Considering the acidity of PFAS with potential for MOF degradation, the emission spectra (Figures S23–S27) and the ultraviolet–visible (UV–vis) absorption spectra (Figures S28–S32) of the single crystal supernatant solutions were recorded after PFAS addition. No obvious luminescence or ligand absorption signals were detected in the UV–vis spectra of the supernatant, which confirms the enhanced luminescence originates from the intact single crystal rather than from the MOF decomposition.

To elucidate the sensing mechanism, a series of characterizations and analyses were carried out. PXRD patterns of the ITHD(Zn) single crystals remained unchanged after the sensing experiments (Figure S33), indicating that the luminescence response is not associated with the structural transformation. The absence of luminescence and ligand absorption signals in the supernatant confirms that the MOF framework remains intact during sensing (Figures S23–S32).^[^ ^39^, ^40^ ^]^ Regarding the excitation energy, UV–vis absorption spectra of the ligands and PFAS (Figures S34–S38) show no spectral overlap, excluding the possibility of competitive absorption between the ligands and PFAS. Further from the excitation spectra of the MOF and PFAS (Figures S39–S43), which also display no overlap, ruling out the competition for excitation energy between them.^[^ ^39^, ^40^ ^]^ Additionally, in terms of the emission energy, no overlap was observed between the MOF emission spectra and the UV–vis spectra of PFAS (Figures S44–S48), indicating that the Förster resonance energy transfer (FRET) process is absent.^[^ ^39^, ^40^ ^]^ Finally for the excited state energy, the lowest unoccupied molecular orbital (LUMO) energy level of the ligands is obviously lower than those of the PFAS (Figure S49), suggesting that the photoinduced electron transfer (PET) does not occur.^[^ ^39^, ^40^ ^]^

To further study the mechanism for the enhanced luminescence, nuclear magnetic resonance (NMR) spectra and calculations were carried out to study the binding modes between MOF and PFAS. To confirm the presence of the PFAS inside the pores, MOFs after soaking in PFAS were digested and their ^19^F NMR spectra were tested (Figures S50–S54), which confirm that PFAS can be soaked by this MOF. Solid‐state NMR spectra were also carried out and analyzed in details (Figure 5). From the ^13^C{^1^H} magic angle spinning (MAS) NMR spectra of PFOA and PFOA@ITHD(Zn) (Figures 5a and S55), PFOA can be confirmed to remain in the pores of the MOF without liquid‐like behavior (fast isotropic motions). In Figure 5b, the ^13^C C─F coupling (CP) MAS NMR spectrum of PFOA@ITHD(Zn) shows reasonably the ─CF_2_, ─CF_3_ resonances. From the ^19^F MAS NMR spectra of PFOA and PFOA@ITHD(Zn) (Figures 5c and S56), there are obvious differences for free PFOA and PFOA in the MOF, which suggests the interaction between PFOA and the MOF structure. In Figure 5d and Table S1, the ─CF_3_ resonances do not change with temperature, which differ by environments of the ─CF_3_ groups of free PFOA and do not experience a movement in the pore spaces of the MOF.^[^ ^41^, ^42^, ^43^, ^44^ ^]^ All these results indicate the binding between the MOF and PFAS, which may decrease the energy consumption of the ligand vibration and induce the enhanced luminescence.^[^ ^45^ ^]^ To investigate the binding interactions, further density functional theory (DFT) calculations were performed. The results revealed that the ligand exhibits binding affinity toward these PFAS, facilitated by multiple types of non‐covalent interactions (Figures S57–S61).^[^ ^46^, ^47^ ^]^ To explore the detailed influence of such bindings, additional calculations about the molecule vibration were conducted, which indicated that the interaction imposes significant constraints on the vibrational freedom of the ligand's phenyl ring (Tables S2–S4 and Figures S62–S64). This restriction effectively reduces non‐radiative energy dissipation pathways of the ligand, thereby inducing the enhanced emission intensity of the MOF.

**Figure 5:**
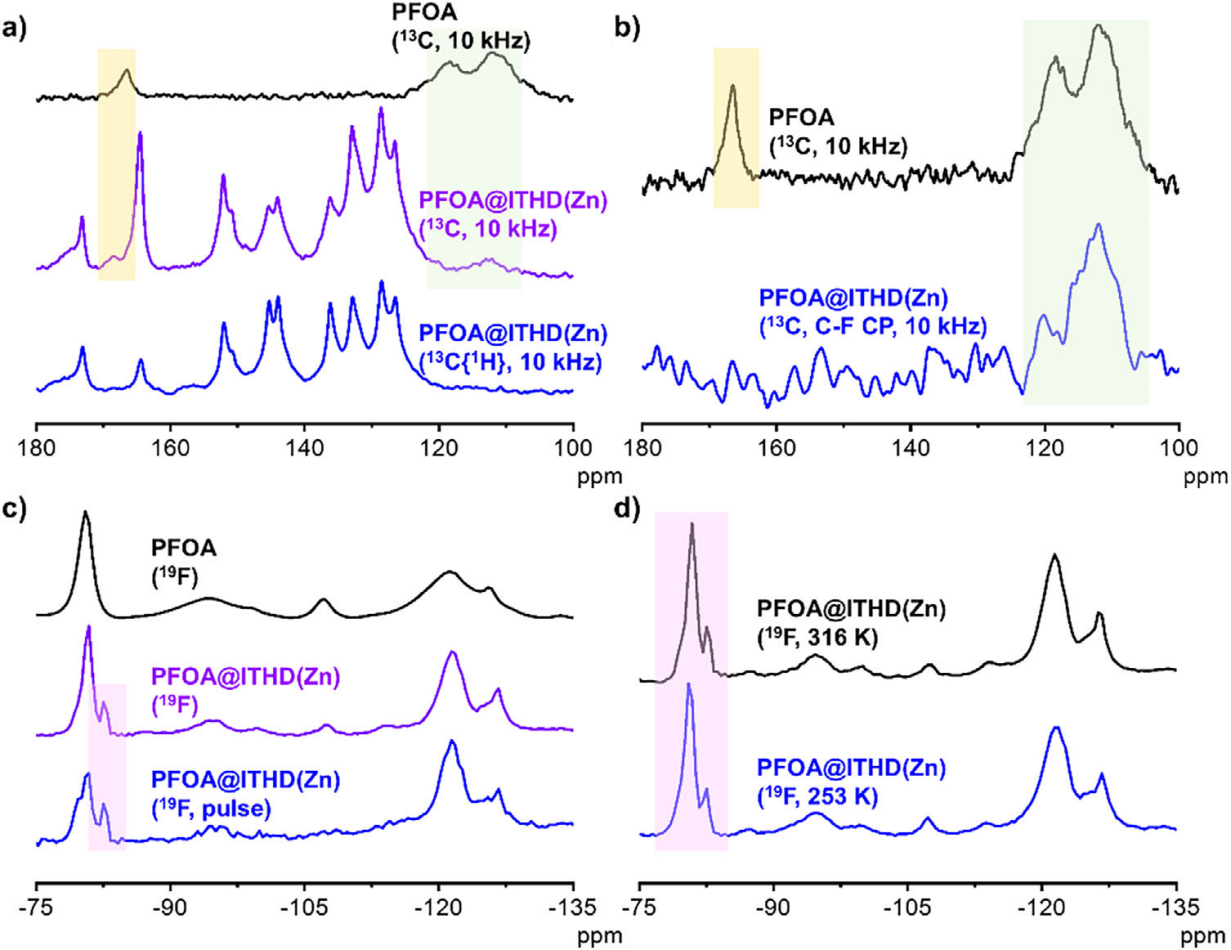
a) The ^13^C{^1^H} MAS NMR spectra recorded with direct excitation of ^13^C nuclei for pure PFOA (10 kHz) and PFOA@ITHD(Zn) (10 kHz), and the ^13^C{^1^H} CP MAS NMR spectra of PFOA@ITHD(Zn) (10 kHz, CP at a contact time of 3 ms). b) The ^13^C F‐C CP MAS NMR spectrum of PFOA@ITHD(Zn) (10 kHz, CP at a contact time of 2 ms) and the ^13^C{^1^H} MAS NMR spectrum of PFOA (10 kHz). c) The low‐field parts of the ^19^F MAS NMR spectra recorded for PFOA (10 kHz), PFOA@ITHD(Zn) (10 kHz), and PFOA@ITHD(Zn) obtained by the inversion‐recovery 180°‐*τ*‐90° pulse sequence at *τ* = 0.65 s (10 kHz). d) The ^19^F MAS NMR spectra of PFOA@ITHD‐Zn at 316 and 253 K (10 kHz).

In summary, a millimeter‐sized luminescent MOF single crystal was synthesized and employed for the detection of various PFAS. Compared to powder‐based luminescent sensors and conventional MOF powders, the single crystal exhibits superior emission stability (RSD = 2.1 × 10^−3^), improved recyclability, and facile handling without the need for dispersion or sonication. Upon exposure to PFAS analytes, including PFOA and its analogues, the crystal displays exponential increases in luminescence intensity, enabling visual detection down to low micromolar concentrations. The emission enhancement is retained over 10 reuse cycles with negligible signal degradation. Mechanistic studies confirm that the luminescence enhancement stems from guest‐framework interactions rather than framework degradation or energy transfer. PXRD and solid‐state NMR indicate structural integrity and PFAS confinement. While the single‐crystal format overcomes suspension and recovery issues, embedding millimeter‐sized MOF crystals into cartridges, optical fiber sensors, or microfluidic devices can enable practical and robust PFAS detection in environmental water. This work establishes a reusable, environmentally stable MOF‐based sensor for PFAS detection and highlights single‐crystal engineering as a powerful strategy to surpass powder‐based systems in speed, accuracy, and energy efficiency.

## Conflict of Interests

The authors declare no conflict of interest.

## Supporting information



Supporting Information

Supporting Information

## Data Availability

The data that support the findings of this study are available in the Supporting Information of this article.
