# Supplementary material for: Enhanced‐Type Quantitative Luminescence Recognition for Per‐ and Polyfluoroalkyl Substances (PFAS) by a Metal–Organic Framework Single Crystal
Source: Angew Chem Int Ed Engl. 2025 Nov 24;65(3):e15775. doi: 10.1002/anie.202515775 (PMC12811649; doi:10.1002/anie.202515775)
Supplement: Supplementary file 1 — Supporting Information [file ANIE-65-e15775-s001.docx]

Supporting Information

**Enhanced-Type Quantitative Luminescence Recognition for Per- and Polyfluoroalkyl Substances (PFAS) by a Metal-Organic Framework Single Crystal**

Zongsu Han,^[a],§^ Yifan Guo,^[b],§^ Kun-Yu Wang,^[a],§^ Wenxuan Li,^[d]^ Jiatong Huo,^[a]^ Qingya Huang,^[e]^ Vladimir I. Bakhmutov,^[a]^ Yihao Yang,^[a]^ Rong-Ran Liang,^[a]^ Peter R. Taylor,^[b]^ Wei Shi^[c],^* and Hong-Cai Zhou^[a],^*

[a] Dr. Z. Han, Dr. K.-Y. Wang, J. Huo, Prof. V. I. Bakhmutov, Y. Yang, R.-R. Liang, Prof. H.-C. Zhou
Department of Chemistry
Texas A&M University
College Station, Texas 77843, United States
E-mail: zhou@chem.tamu.edu

[b] Y. Guo, Prof. P. R. Taylor
School of Pharmaceutical Science and Technology
Tianjin University
Tianjin 300072, China

[c] Prof. W. Shi
Frontiers Science Center for New Organic Matter, State Key Laboratory of Advanced Chemical Power Sources and Key Laboratory of Advanced Energy Materials Chemistry (MOE), College of Chemistry
Nankai University
Tianjin 300071, China
E-mail: shiwei@nankai.edu.cn

[d] Dr. W. Li
Department of Chemistry
Princeton University
Princeton, New Jersey 08544, United States

[e] Q. Huang
Department of Materials Science and Engineering
University of Pennsylvania
Philadelphia, Pennsylvania 19104, United States

**Methods and analysis**

**Materials and methods**

All reagents were commercially available and used without further purification. Liquid ^19^F NMR spectra were recorded on a Bruker Avance NEO 400 NMR spectrometer. Single crystal X-ray diffraction patterns were collected by a Bruker-Axs Venture Ius Cmos Kappa X-ray Apex2 diffractometer with Cu-Kα radiation. The structures were solved by SHELXS (direct methods) and refined by SHELXL (full matrix least-squares techniques) in the Olex2 package.^[1,2]^ CCDC number 2383870 is for ITHD(Zn). PXRD measurements were performed using a Bruker Powder-ECO X-ray diffractometer with Cu-Kα radiation. Thermo-gravimetric analysis curves were obtained under nitrogen atmosphere on a Mettler Toledo TGA/DSC 1 thermogravimetric analyzer from 50 °C to 800 °C. Luminescence spectra were recorded on a Shimadzu RF-5301 fluorescence spectrophotometer. UV-vis absorption spectra were measured by a Shimadzu UV-2450 absorption spectrometer. 3 mg MOFs for liquid NMR tests were dissolved by 0.5 mL *d*_6_-DMSO and 5 μL D_2_SO_4_.

**Synthesis**

ITHD(Zn) was synthesized with a slightly modified method according to the literature.^[3]^ 76 mg Zn(NO_3_)_2_·6H_2_O, 88 mg H_3_BTB, and 18 mg 4,4’-BPY were dissolved in 10 mL dry DMF in a 20 mL glass vial. The solution was heated in an oven at 80 °C for three days, resulting in colorless millimeter-scale single crystals, which were collected by filtration and washed with fresh DMF and acetone, respectively.

**Luminescence experiments**

One single crystal was placed in the cuvette for test. For comparison tests, single crystals were ground into fine powder and dispersed in DMF by ultrasound for 10 minutes to form a clear suspension with a concentration of 0.3 mg mL^-1^.

**Solid-state NMR measurements**

The ^13^C{^1^H} and ^19^F MAS NMR experiments were carried out with a Bruker Avance-NEO solid-state NMR spectrometer (400 MHz for ^1^H nuclei) equipped with a standard two-channel 4-mm MAS probe head. The external references were TMS and CCl_3_F for ^13^C and ^19^F nuclei, respectively. The ^19^F MAS NMR spectra were recorded with a single-pulse sequence using one scan with the pulse length of 3.5 μs (90°), a relaxation delay of 20 s and at spinning rates 10 and 11 kHz. The ^19^F T_1_ times were determined with a standard inversion-recovery (180°-τ-90°) experiments with τ variations between 20 and 0.001 s. The ^13^C{^1^H} CP MAS NMR spectra were obtained at spinning rate from 8 to 12 kHz with standard cross-polarization pulse sequence at ^1^H pulse of 2.5 μs (90°), CP contact times of 2.0 and 9.0 ms (power of 77.2 W), 1600 scans, and relaxation delays of 5 s. The standard *tppm15* pulse sequence has been used for high power ^1^H decoupling. The ^13^C{^1^H} MAS NMR spectra were obtained by ^13^C direct excitation with the pulse of 2.5 μs (60°), relaxation delays of 10 s using 12000 scans.

**Solid-state NMR analysis**

^13^C MAS NMR spectra were shown in Figures 5a, 5b, and S55.

For PFOA, the spectrum shows the resonance at δ(iso) of 167.0 ppm, assigned to -**C**OO^-^ group and two overlapped resonances at δ(iso) of 118.7 and 112.6 ppm corresponding to -(**C**F_2_)_6_**C**F_3_ groups (Figures 5a and S55).

For the solvents inside the MOF, the spectrum exhibits the sharp resonances belonging to DMF (164.5, 37.5 and 32.2 ppm) and acetone (207.5 and 31.6 ppm) (Figures 5a and S55). It is remarkable that the solvent signals remain sharp and Lorenz-shaped even in the static spectrum, illustrating their liquid-like behavior (fast isotropic motions) in the pores of the MOF (Figure S55).

For the framework, the MOF shows the resonances at δ(iso) of 175.0 and 173.2 ppm belonging to the carbon atoms of non-equivalent carboxyl groups (BTB ligand) and 152.2, 151.0, 145.5, 144.1, 136.4, 133.0, 128.7 and 126.6 ppm belonging to the carbon atoms of aromatic rings (BTB and BPY ligands) (Figures 5a and S55).

For the PFOA inside the MOF, the resonances of incorporated PFOA are observed in PFOA@ITHD(Zn) as low-intense resonances at δ(iso) of 168.5, 118.7 and 112.6 ppm, which disappear in the ^13^C{^1^H} CP MAS NMR experiments even at a long cross-polarization time of 9 ms targeted to long proton-carbon distances, confirming that they belong to PFOA (Figures 5a and S55). It is also remarkable that the ^13^C F-C CP MAS NMR spectrum of PFOA@ITHD(Zn) shows reasonably -(**C**F_2_)_6_**C**F_3_ resonances (Figure 5b). The above analysis can confirm the existence of PFOA in the pore of the MOF.

^19^F MAS NMR spectra were shown in Figures 5c, 5d, and S56.

For PFOA, it shows three isotropic resonances at δ(iso) of -80.6. -121.3 and -125.6 ppm, assigned to -C**F_3_**, -(C**F_2_**)_5_- and -C**F_2_**-CF_3_ groups, respectively (Figures 5c and S56). As reported,^[4,5]^ these resonances are accompanied by intense spinning sidebands caused by chemical shift anisotropy of ^19^F nuclei and remaining ^19^F-^19^F dipolar interactions. It is remarkable that the -C**F_3_** linewidth at a spinning rate of 10 kHz is determined for PFOA as 665 Hz versus 330 Hz determined earlier for PFOA spinning at 16 kHz.^[4]^ Since the ^3^J(^19^F-^19^F) are small (10-15 Hz), such a change provides to conclude that the main factor influencing on the resonance linewidths is the remaining ^19^F-^19^F dipolar interactions. According to the ^19^F inversion-recovery experiments performed for PFOA at a spinning rate of 10 kHz in the present work, the ^19^F T_1_ times of -C**F_3_**, -(C**F_2_**)_5_- and -C**F_2_**-CF_3_ groups are calculated as 2.4, 2.3 and 2.3 s, respectively, similarly to 1.6 ± 0.3 s and 2.8 ± 1.3 s, reported for PFOA at a spinning rate of 16 kHz.^[4]^ It is interesting that for compound SPFO, these ^19^F T_1_ times obtained at a spinning rate of 20 kHz^[5]^ are reported as 2.655 (-C**F_3_**) and 3.104 s (-C**F_2_**-). Such a T_1_ elongation at increasing of the spinning rate could correspond to the presence of ^19^F spin-diffusion,^[5,6]^ which can potentially contribute to the relaxation process.

For the PFOA inside the MOF, in contrast to pure PFOA, the ^19^F MAS NMR spectrum of PFOA molecules located in the pores of the MOF display three -C**F_3_** resonances with δ(iso) of -79.4, -80.5 and -82.5 ppm (Figure 5c). These signals are more clearly observed in the inversion-recovery 180°-τ-90° spectra obtained with a τ time of 0.65 s. It should be noted that the -C**F_2_**- and -C**F_2_**-CF_3_ groups detected at δ(iso) of -120.5 and -126.4 ppm do not show such an effect. It should be emphasized that the linewidths and sideband intensities of PFOA resonances incorporated into MOF decrease strongly. This phenomenon suggests that this effect can be explained by removing the intermolecular ^19^F-^19^F dipolar interactions, which are present in free PFOA. Besides, the -C**F_3_** resonances do not change with temperature (Figure 5d). Thus, the PFOA molecules within the pore of MOF spectroscopically show various states, different from the -C**F_3_** groups of free PFOA and do not experience a mutual exchange among these states in the pore spaces of MOF, which suggests the binding between the MOF and PFOA. To characterize the mobility of PFOA molecules in the pores of the MOF, the variable-temperature experiments were carried out (Table S1), which illustrates the isotropic chemical shifts measured at different temperatures. The results show that there are limited changes of the -C**F_3_** δ(iso) values with the temperature. Thus, the temperature does not affect the interactions of PFOA with the MOF.

**Calculation methods**

All calculations were carried out using density functional theory (DFT) with the M06-2X functional^[7]^ and the def-TZVP basis set,^[8]^ as implemented in the Turbomole 7.5 program package.^[9]^ The molecular geometries were fully optimized without symmetry constraints, followed by vibrational frequency analyses to confirm the absence of imaginary frequencies (all real frequencies confirmed true minima on the potential energy surface).

**Calculation analysis**

To understand the enhanced mechanism, we carried out a series of theoretical calculations focusing on the rotational flexibility of the H_3_BTB ligand. The inherent rotational freedom of the ligand’s phenyl rings is known to facilitate non-radiative energy dissipation, which can reduce the emission intensity. Therefore, restricting such motions through guest-ligand interactions could potentially enhance the emission intensity.

To elucidate how PFAS regulates the photophysical behavior of the H_3_BTB ligand system, we first investigated the nature of noncovalent interactions between the two components through reduced density gradient (RDG) analysis.^[10]^ The RDG function is defined as:

$$RDG\left( r \right)= \frac{1}{2{(3\pi^{2})}^{1/3}}\cdot\frac{\mid\nabla\rho(r)\mid}{{\rho(r)}^{4/3}}$$

where ρ(r) is the electron density and ∇ρ(r) is its gradient. The RDG isosurfaces mapped with sign(λ_2_)ρ were computed using **Multiwfn** and visualized in **VMD**.^[11,12]^ As shown in the RDG map, **green spindle-shaped regions** at the H_3_BTB and PFAS interface indicate van der Waals interactions, while **blue regions** suggest hydrogen bonding between the carboxyl group of PFAS and the hydrogen atoms on the H_3_BTB phenyl rings (Figures S57-61). These findings confirm that **multiple weak interactions spatially restrict the** H_3_**BTB molecule**, particularly around its aromatic framework.

To assess the impact of such noncovalent interactions on molecular dynamics, we performed a **vibrational frequency analysis**. The results show that out-of-plane modes of the H_3_BTB phenyl rings exhibited **increased frequencies** with the addition of tetrafluorosuccinic acid, heptafluorobutyric acid, and perfluorobutanesulfonic, suggesting an increase in structural rigidity (Figure S62 and Table S2). According to the harmonic oscillator model:

$$\nu= \frac{1}{2\pi}\sqrt{\frac{k}{\mu}}$$

where *ν* is the vibrational frequency, *k* is the force constant, and *μ* is the reduced mass. Higher frequencies typically indicate a stiffer molecular environment. This aligns with the **Restriction of Intramolecular Vibrations (RIV)** mechanism, which suppresses non-radiative decay and contributes to enhanced luminescence.^[13]^

In contrast, the complexation with perfluorooctanoic acid and perfluorobutanesulfonic acid led to an asymmetric response: while two phenyl rings showed increased vibrational frequencies, one exhibited a slight decrease. To further interpret the effect, we examined the **atomic displacement amplitudes** based on the eigenvectors. The displacement magnitude *d_i_* for each atom was calculated as:

$$d_{i}= \sqrt{{dx}^{2}+{dy}^{2}+{dz}^{2}}$$

and the root-mean-square (RMS) amplitude was computed as:

$$RMS= \sqrt{\frac{1}{N}\sum_{i=1}^{N} d_{i}^{2}}$$

The amplitudes of atomic displacements were significantly reduced of the complexation with perfluorooctanoic acid and perfluorobutanesulfonic acid (Figures S63, S64, and Tables S3, S4). This confirms that the observed slight softening in vibrational energy does not reflect increased flexibility, but rather a suppression of vibrational freedom due to spatial constraints from noncovalent interactions.

**Figures**

**
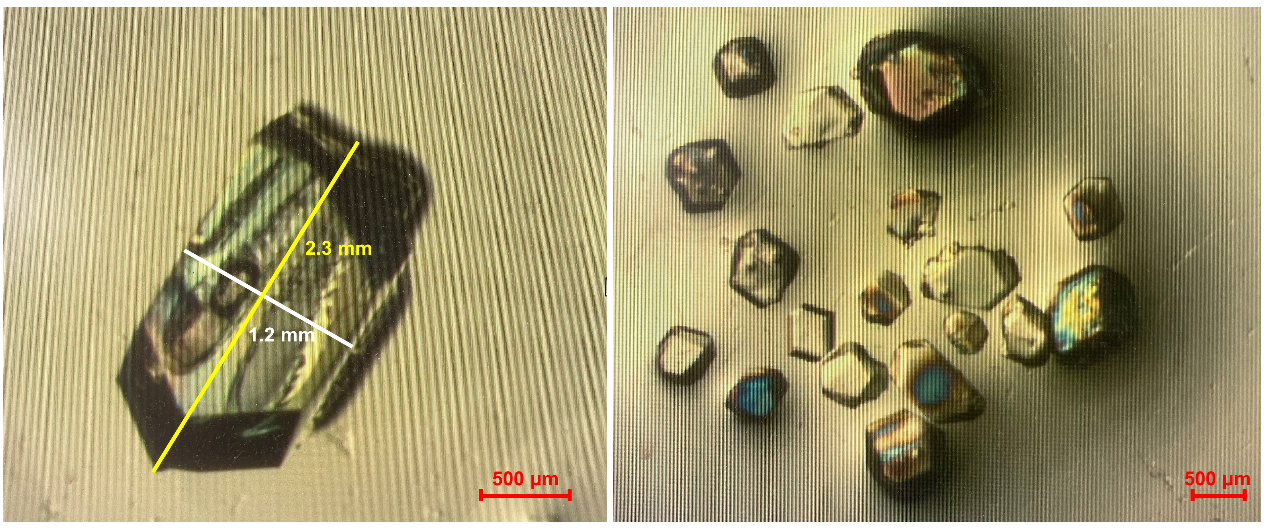
**

**Figure S1.** Single crystals of ITHD(Zn) under microscope.

**
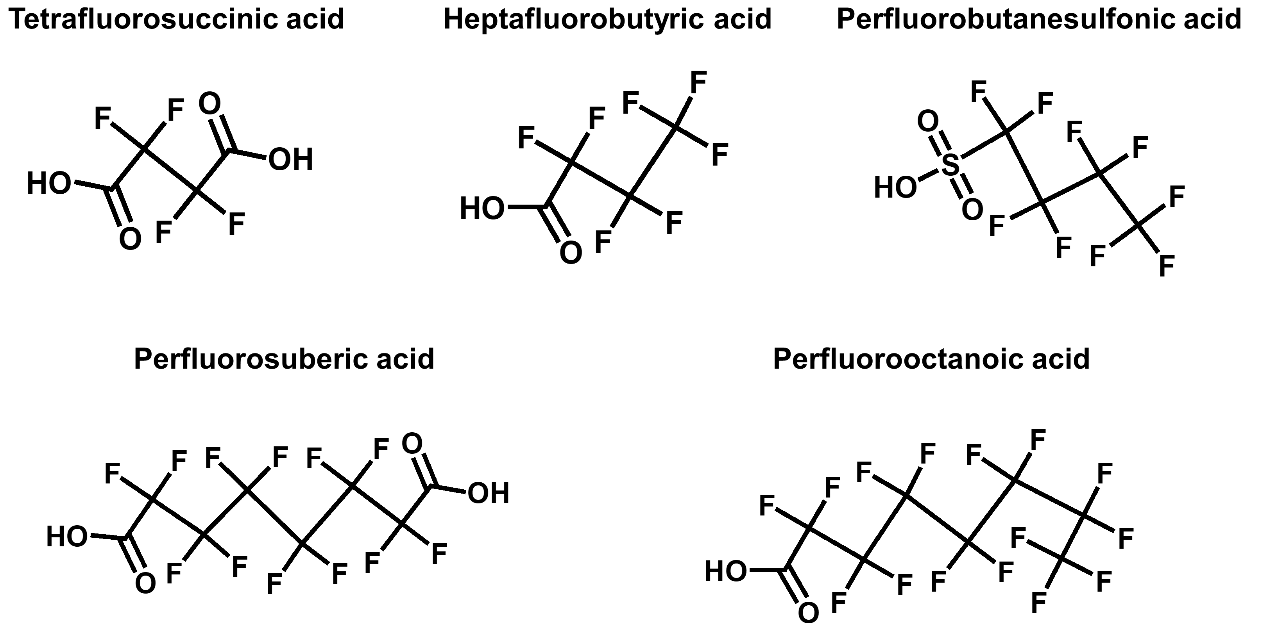
**

**Figure S2.** PFAS analytes in this work.

**
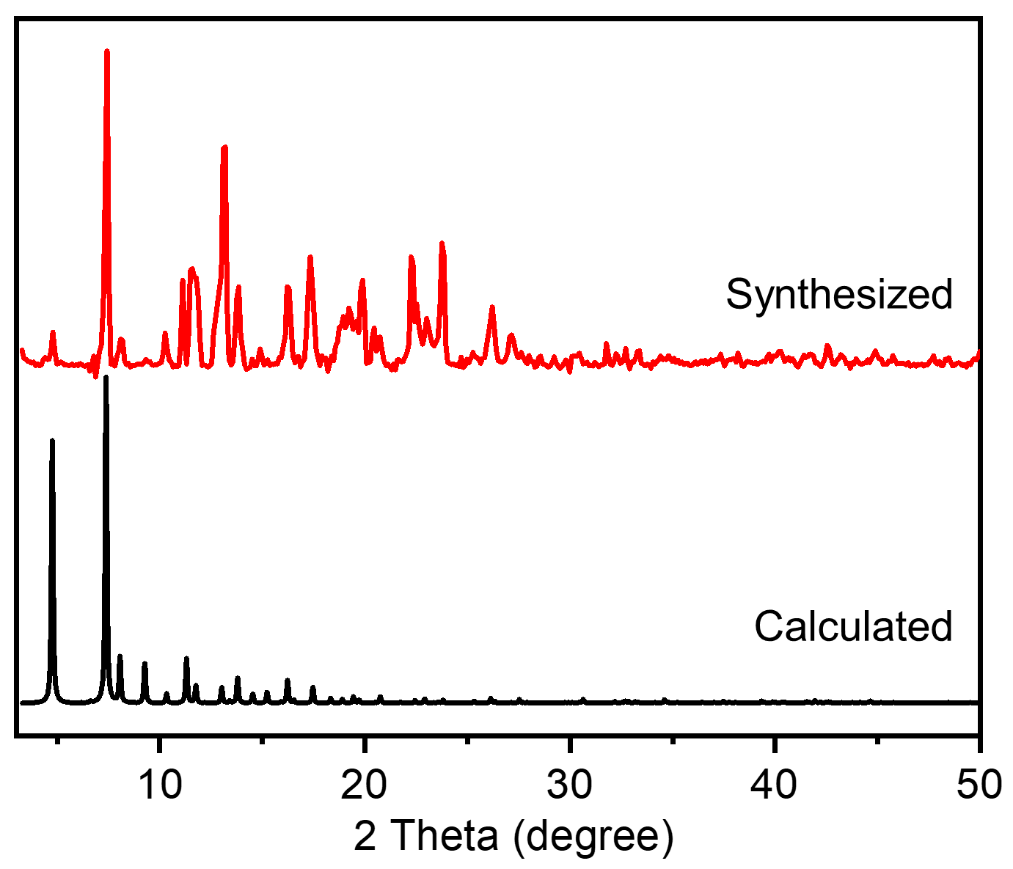
**

**Figure S3.** PXRD pattern of ITHD(Zn).

**
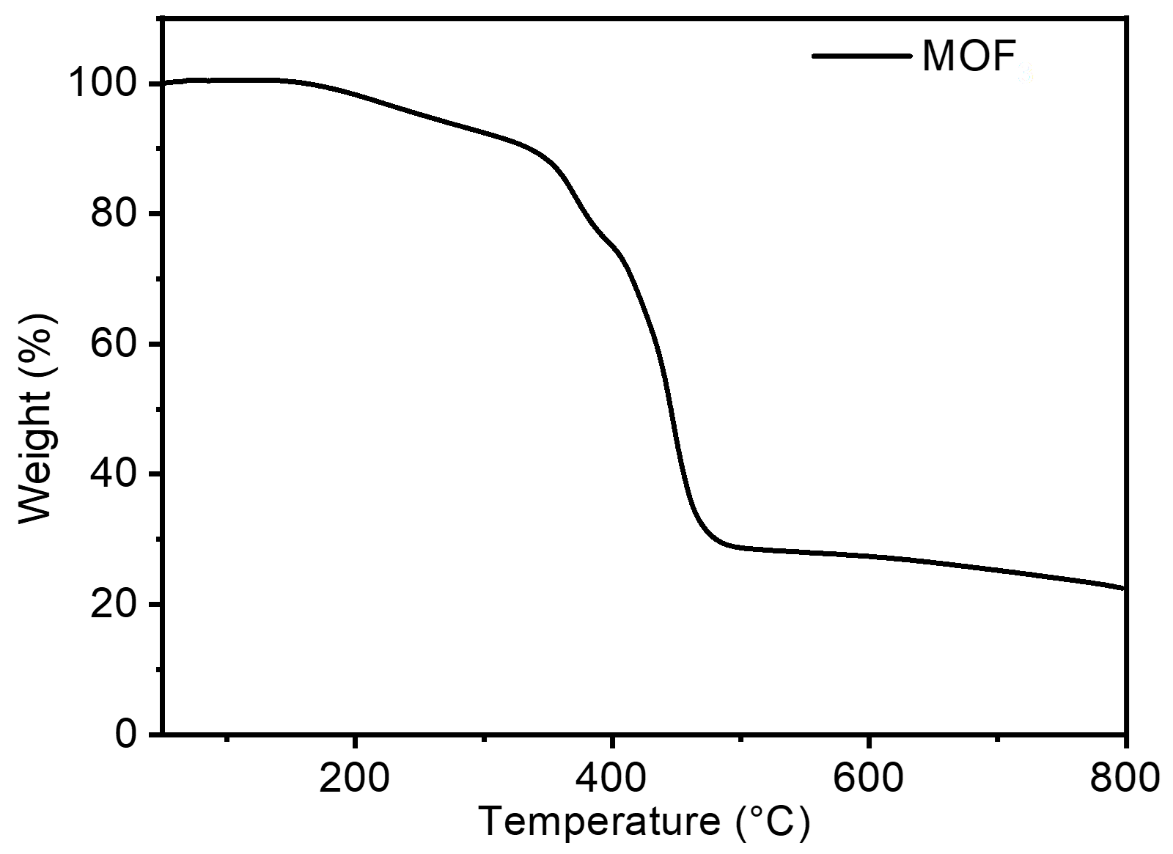
**

**Figure S4.** TGA curve of ITHD(Zn).

**
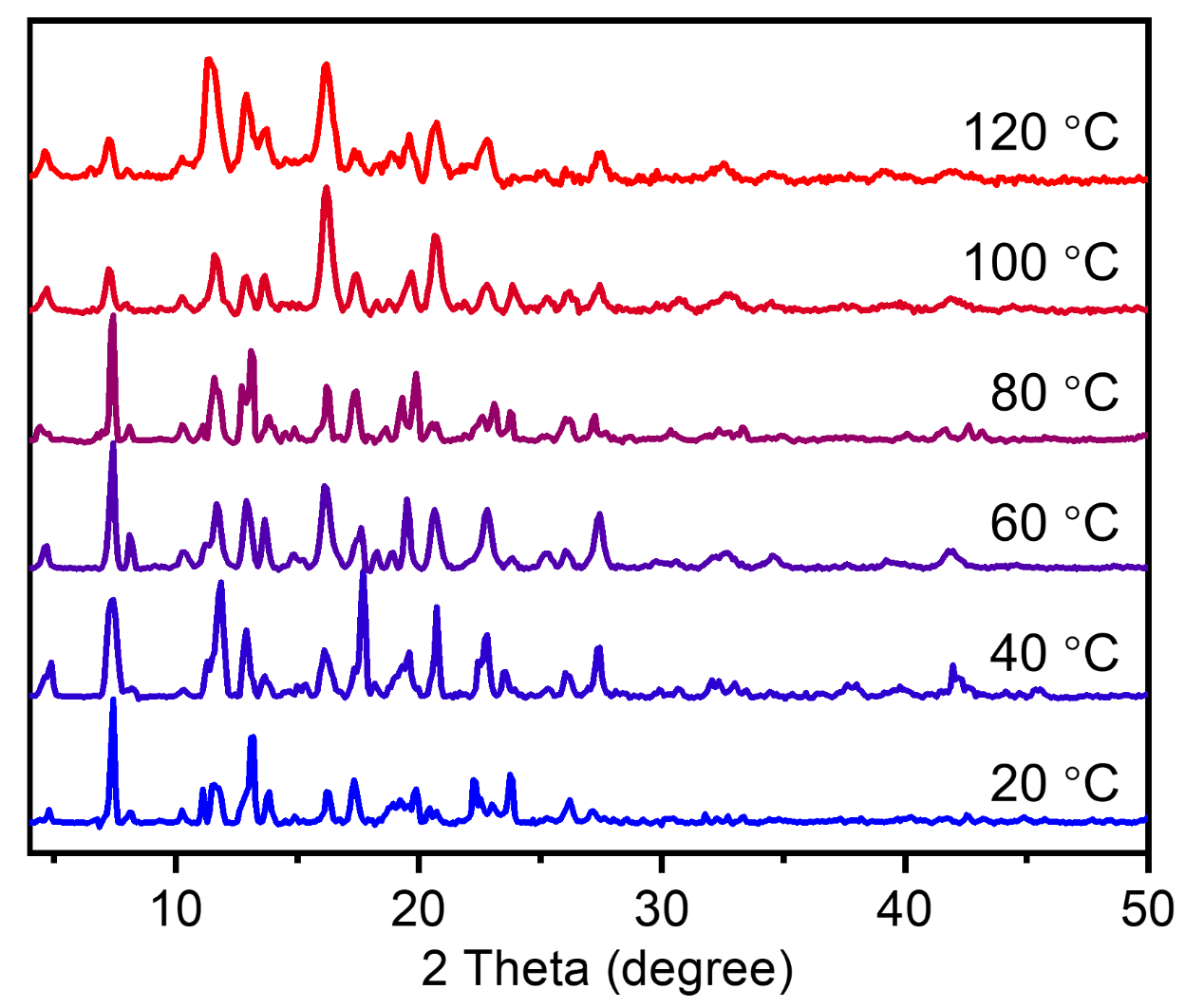
**

**Figure S5.** PXRD patterns of ITHD(Zn) with temperature changes.

**
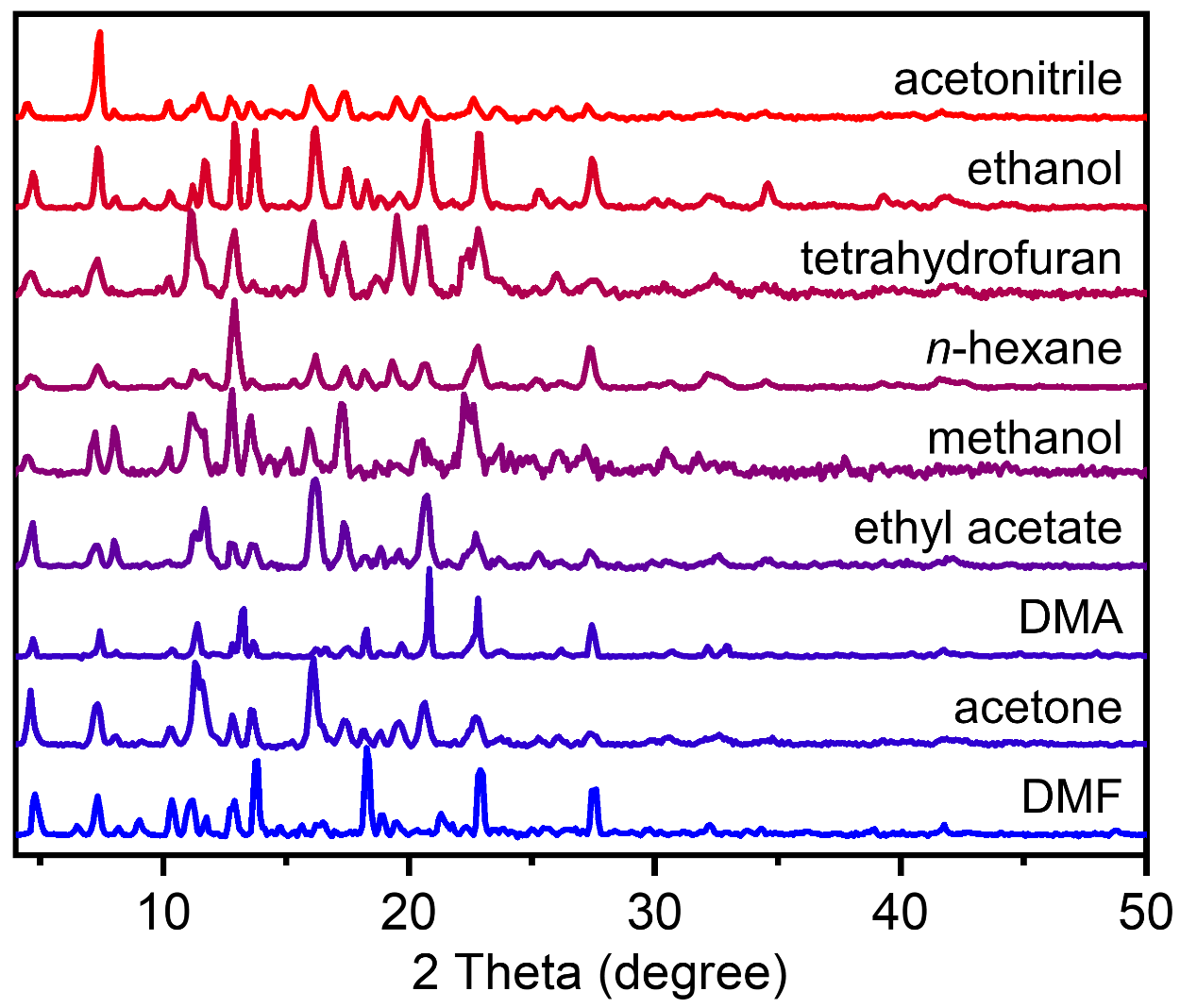
**

**Figure S6.** PXRD patterns of ITHD(Zn) in different solvents.

**
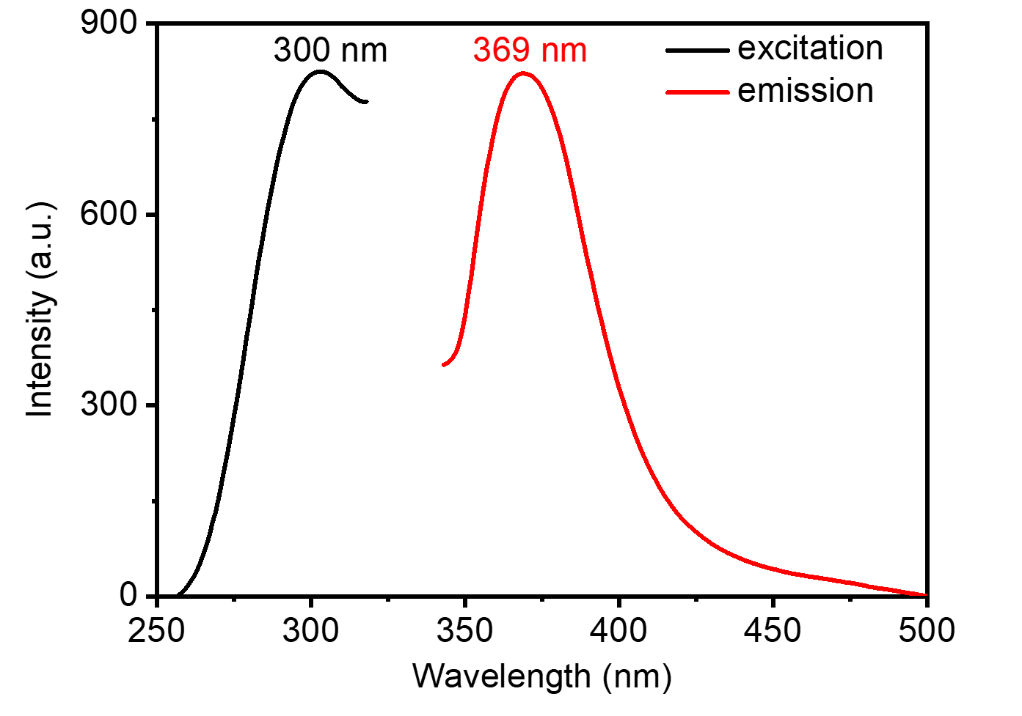
**

**Figure S7.** Excitation and emission spectra of ITHD(Zn).

**
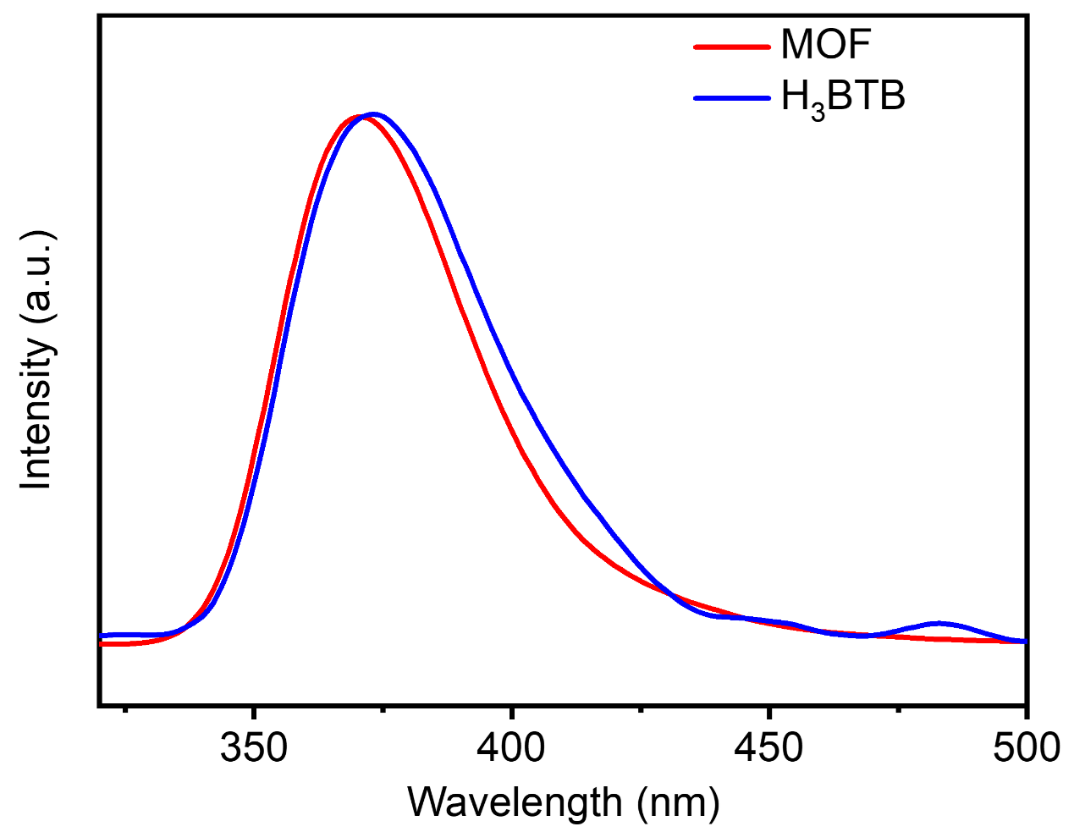
**

**Figure S8.** Emission spectra of ITHD(Zn) and H_3_BTB.

**
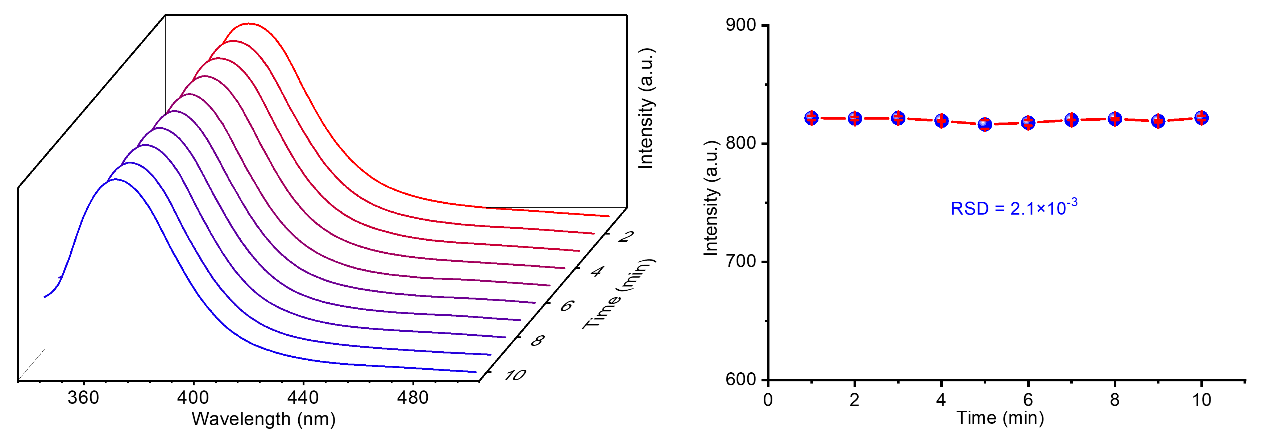
**

**Figure S9.** Emission spectra and intensity changes of the single crystal of ITHD(Zn) with time.

**
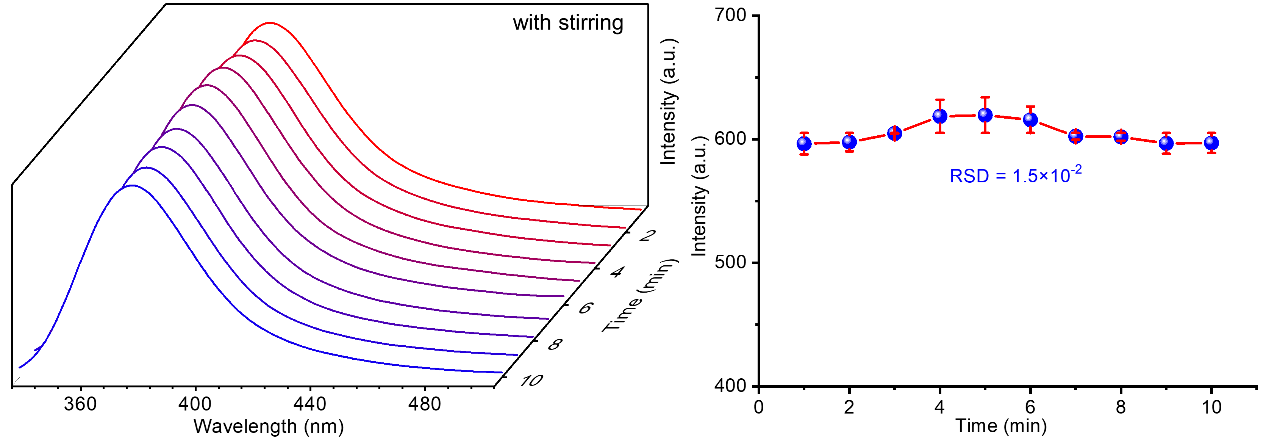
**

**Figure S10.** Emission spectra and intensity changes of the ground powders of ITHD(Zn) with time with continuous stirring.

**
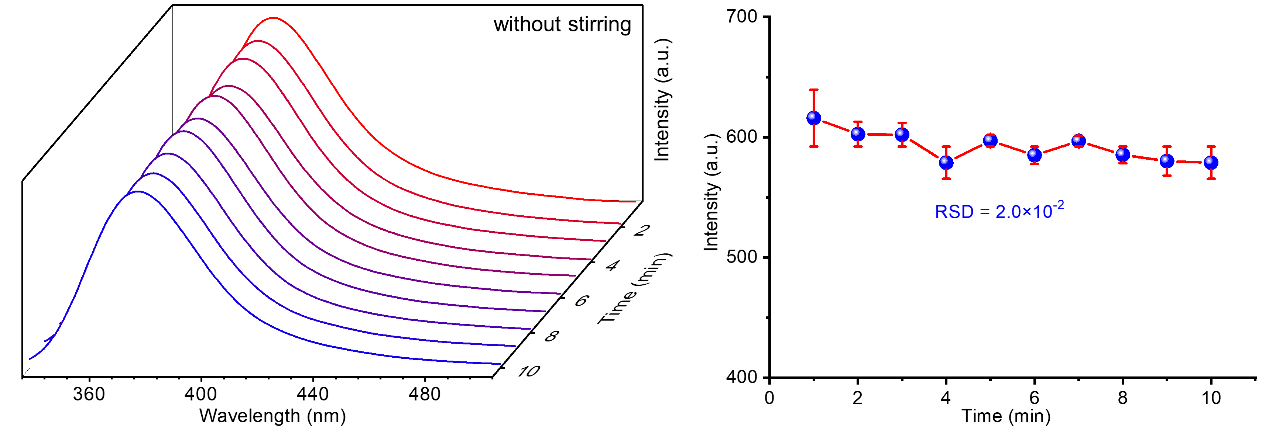
**

**Figure S11.** Emission spectra and intensity changes of the ground powders of ITHD(Zn) with time without stirring.

**
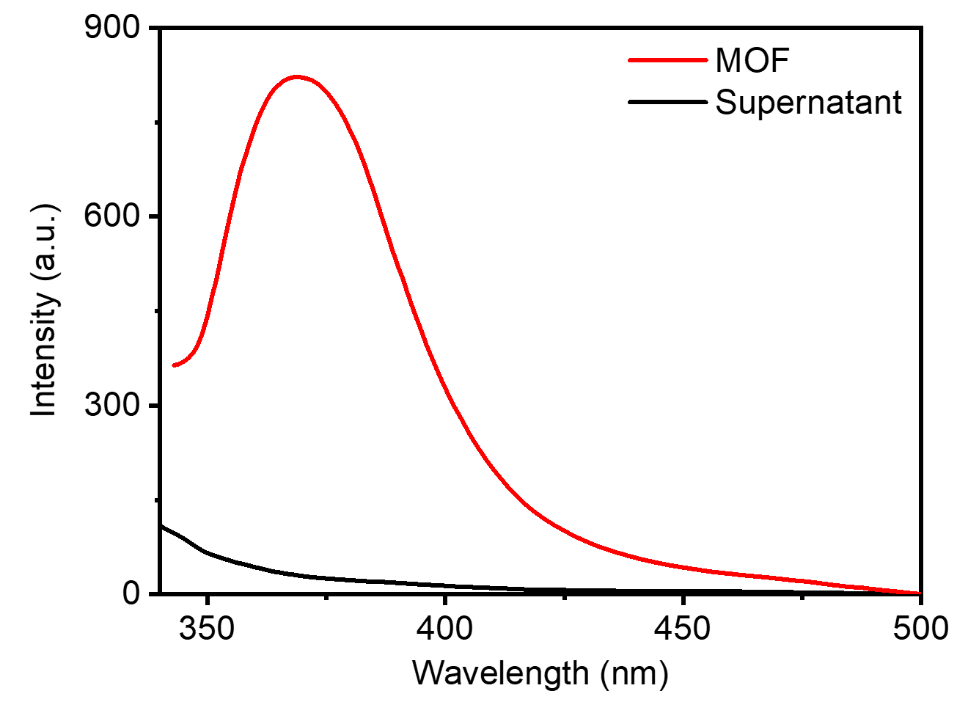
**

**Figure S12.** Emission spectra of ITHD(Zn) and its supernatant.

**
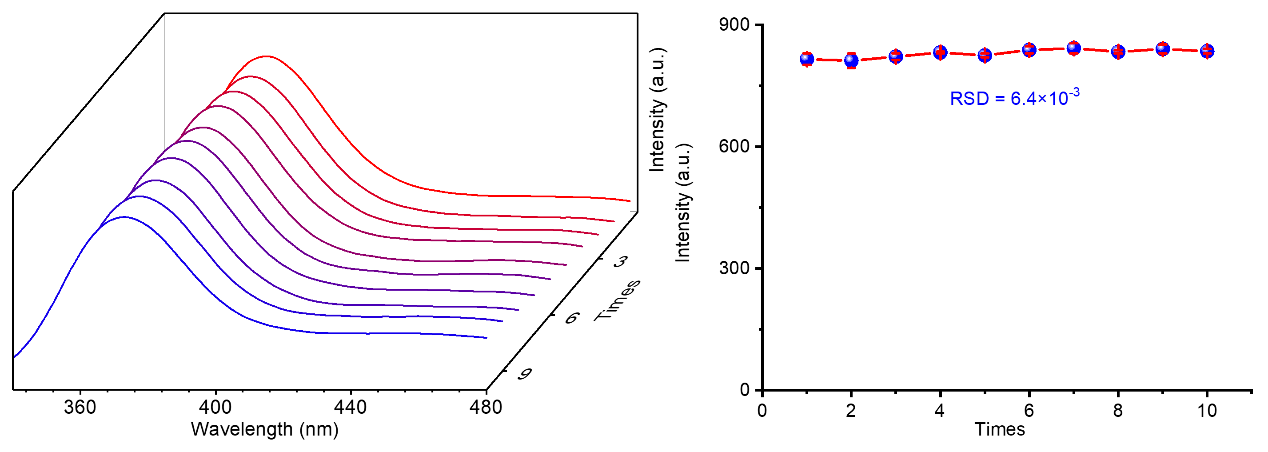
**

**Figure S13.** Emission spectra and intensity changes of ITHD(Zn) with recycled times.

**
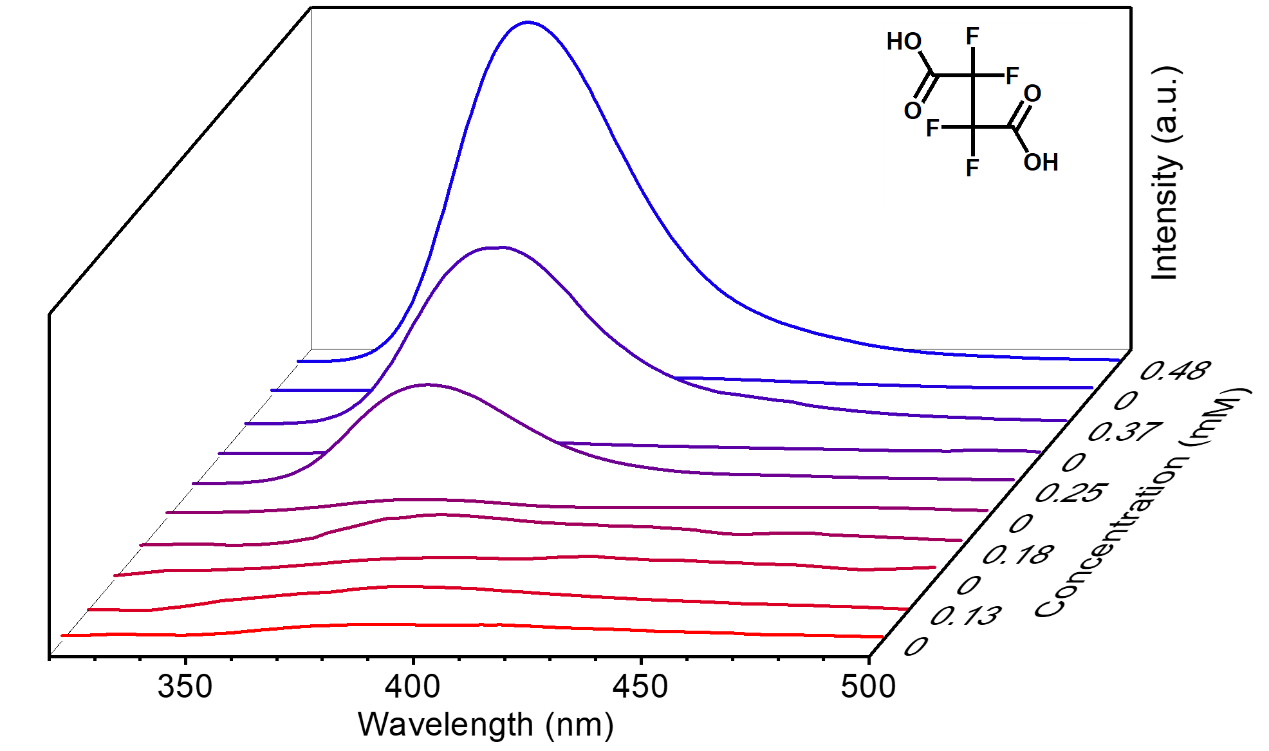
**

**Figure S14.** Emission spectra of ITHD(Zn) with the additions of tetrafluorosuccinic acid.

**
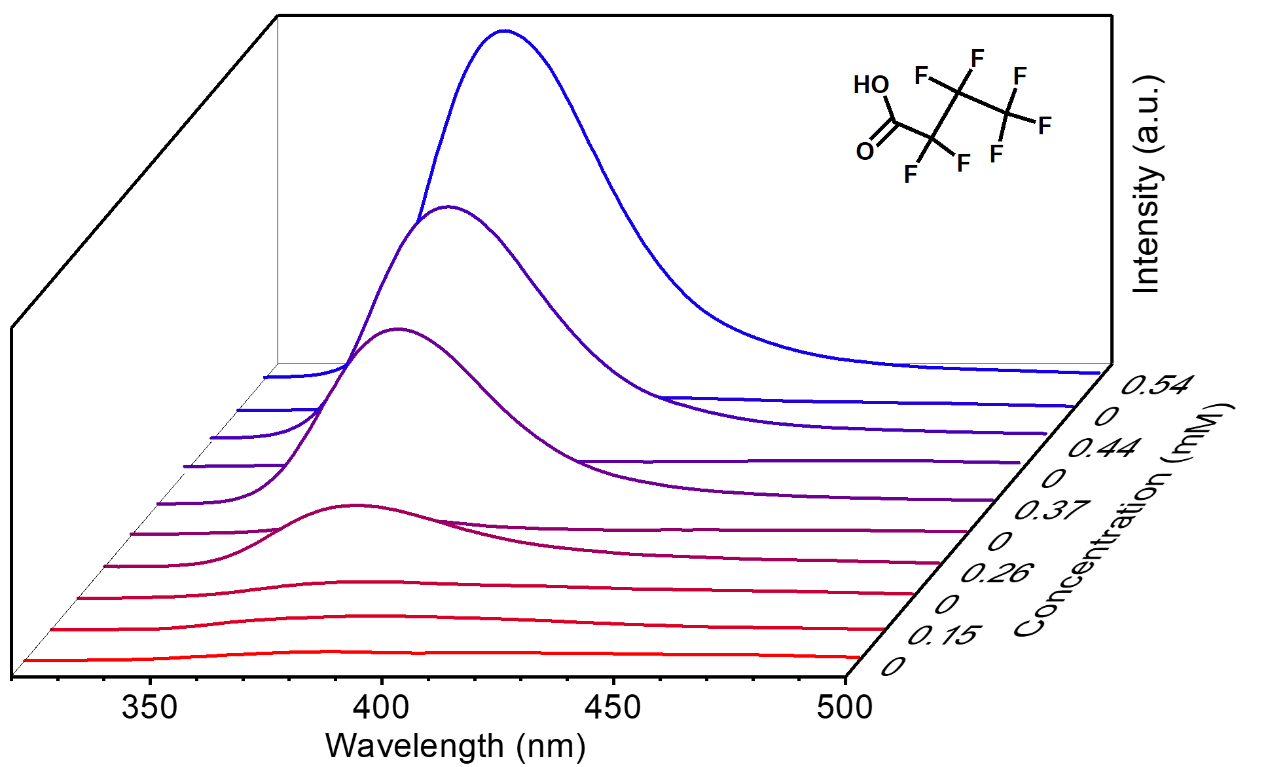
**

**Figure S15.** Emission spectra of ITHD(Zn) with the additions of heptafluorobutyric acid.

**
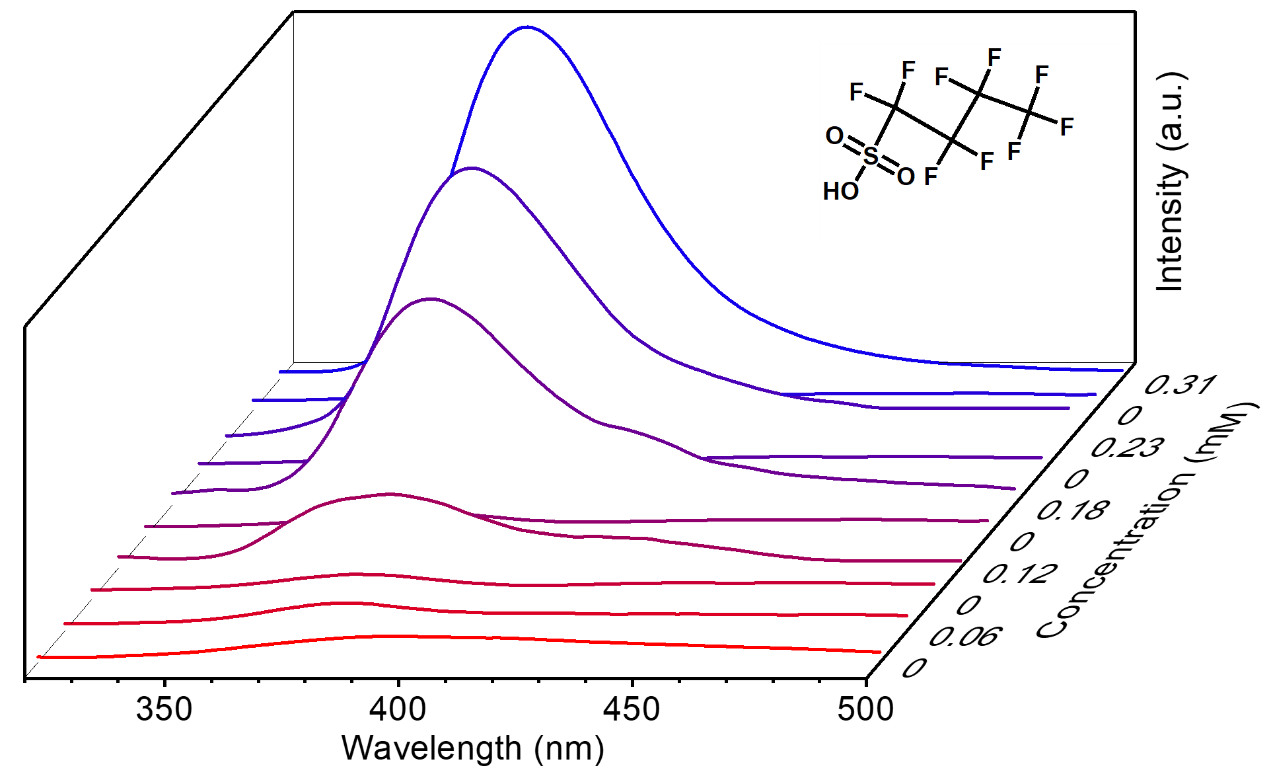
**

**Figure S16.** Emission spectra of ITHD(Zn) with the additions of perfluorobutanesulfonic acid.

**
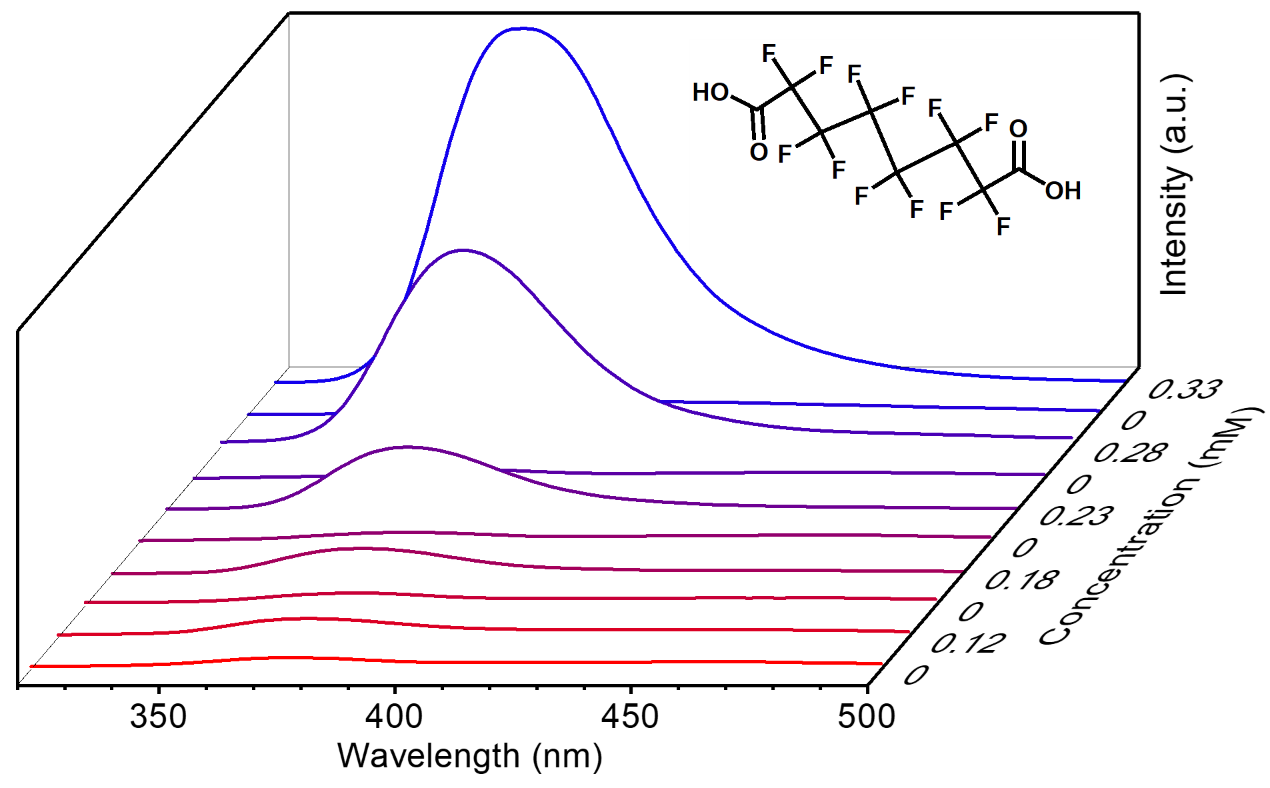
**

**Figure S17.** Emission spectra of ITHD(Zn) with the additions of perfluorosuberic acid.

**
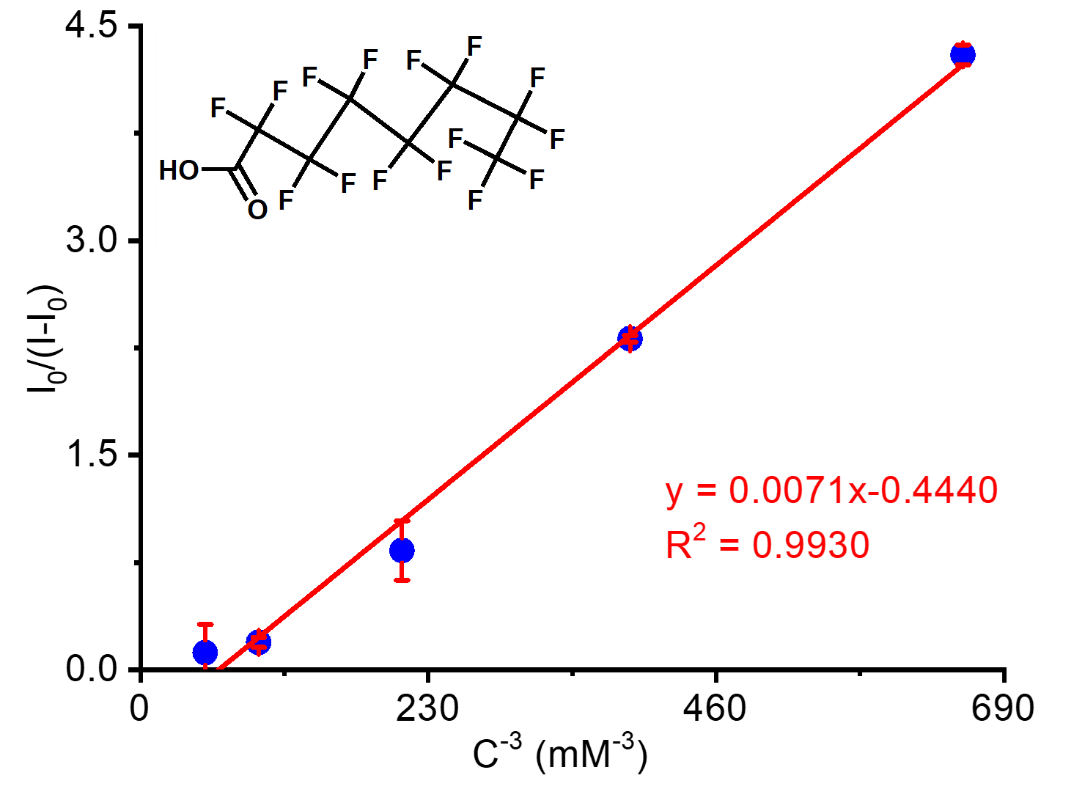
**

**Figure S18.** Emission intensity changes of the MOF single crystal upon exposure to different concentrations of PFOA.

**
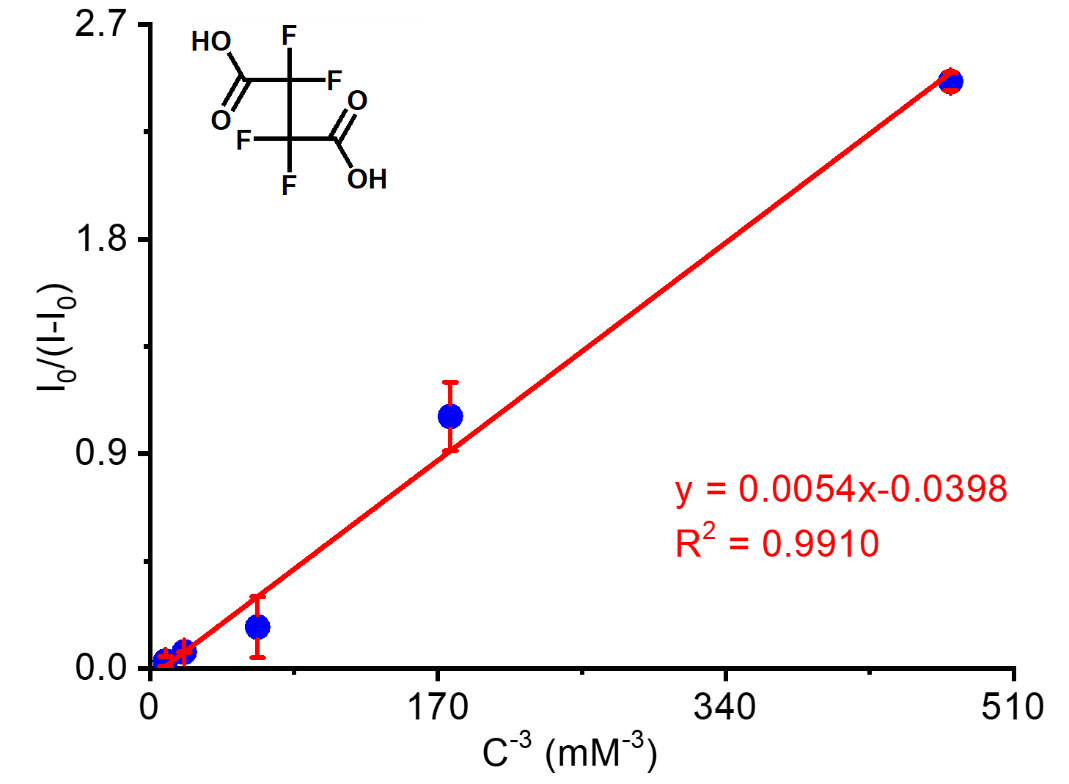
**

**Figure S19.** Emission intensity changes of the MOF single crystal upon exposure to different concentrations of tetrafluorosuccinic acid.

**
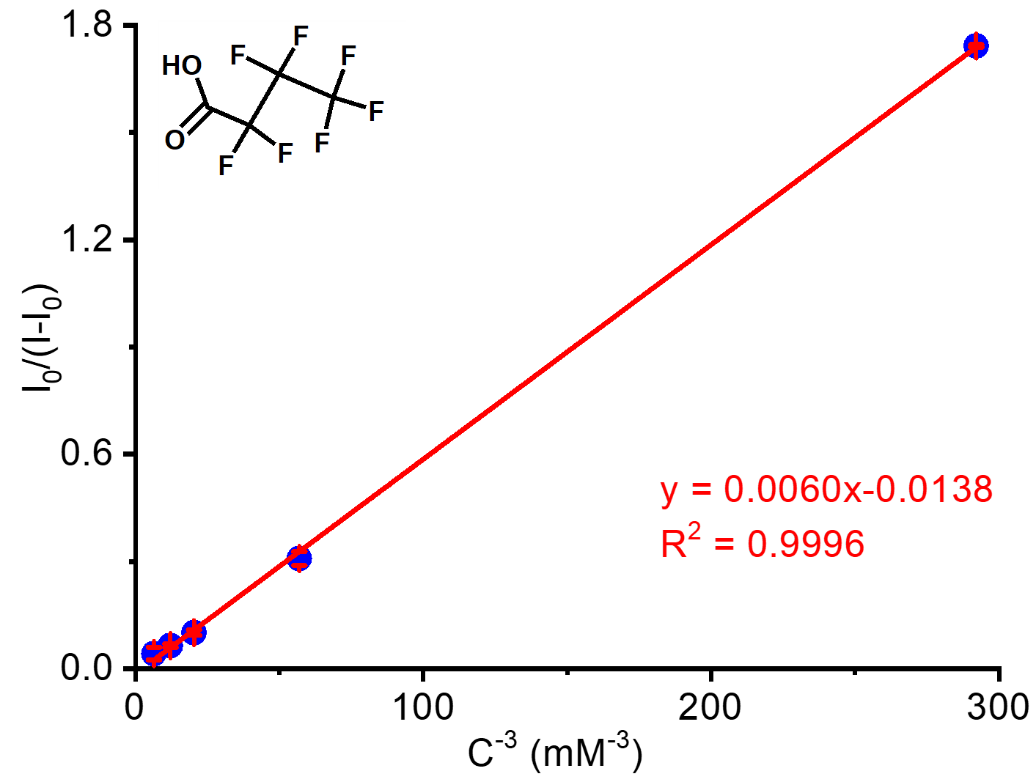
**

**Figure S20.** Emission intensity changes of the MOF single crystal upon exposure to different concentrations of heptafluorobutyric acid.

**
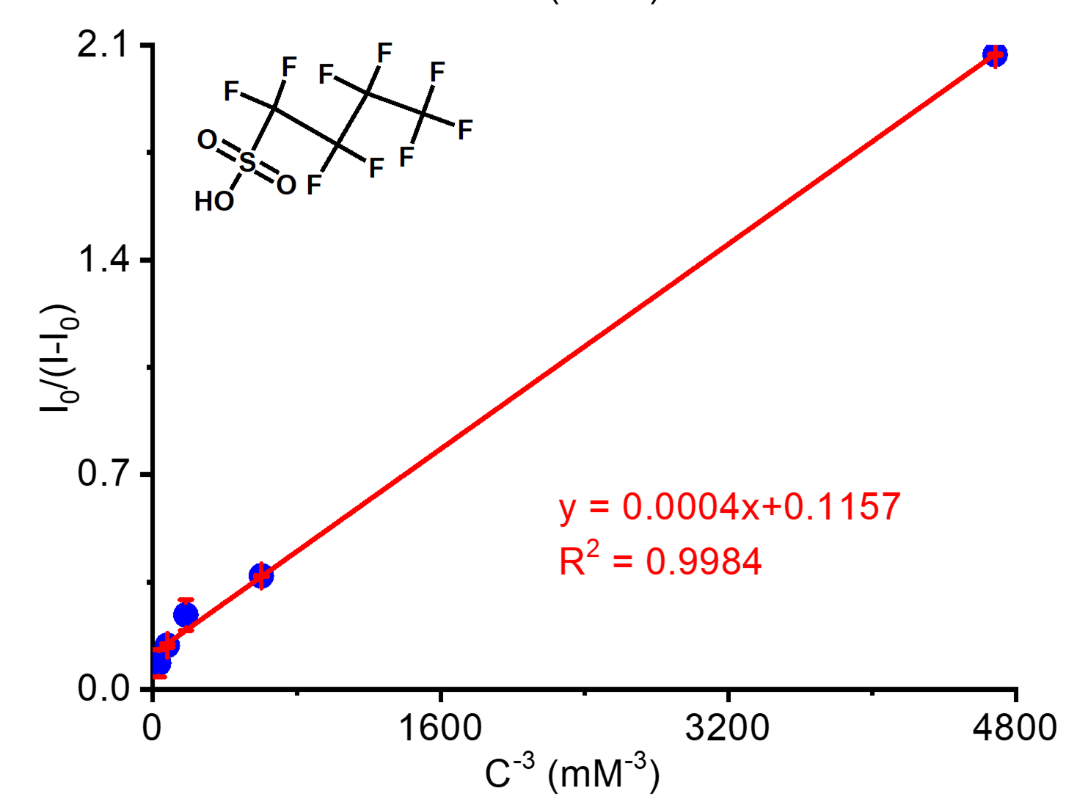
**

**Figure S21.** Emission intensity changes of the MOF single crystal upon exposure to different concentrations of perfluorobutanesulfonic acid.

**
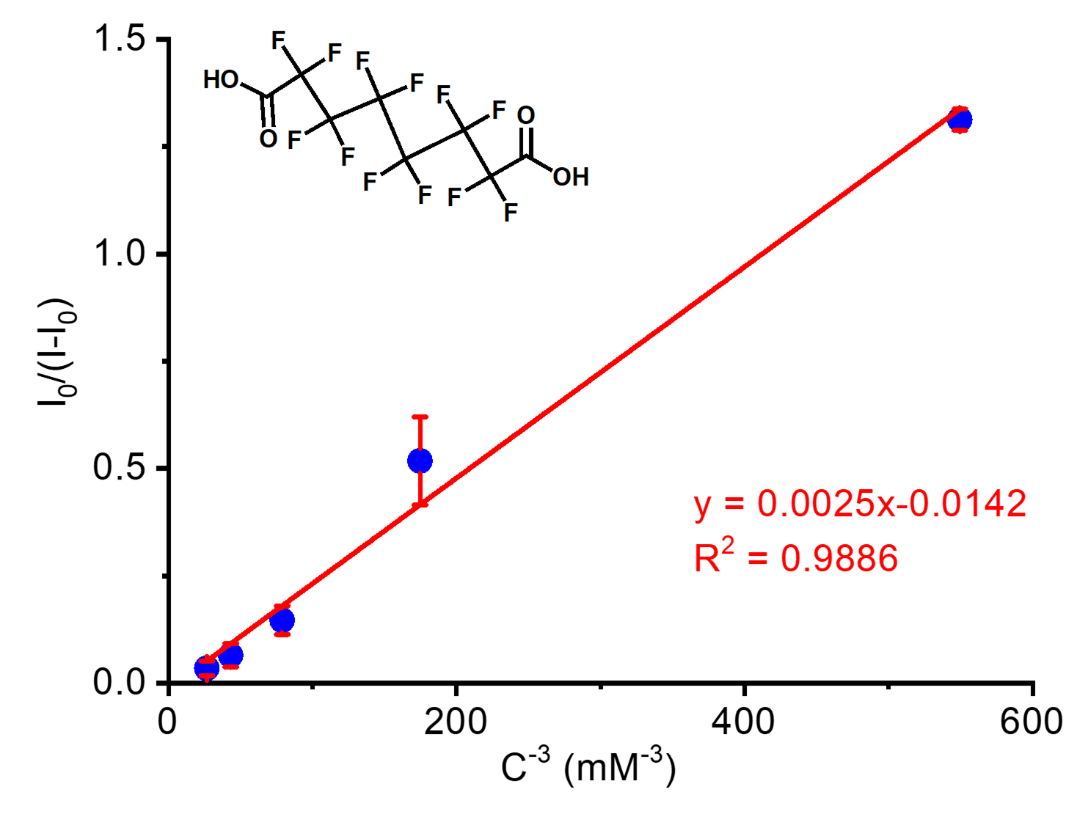
**

**Figure S22.** Emission intensity changes of the MOF single crystal upon exposure to different concentrations of perfluorosuberic acid.

**
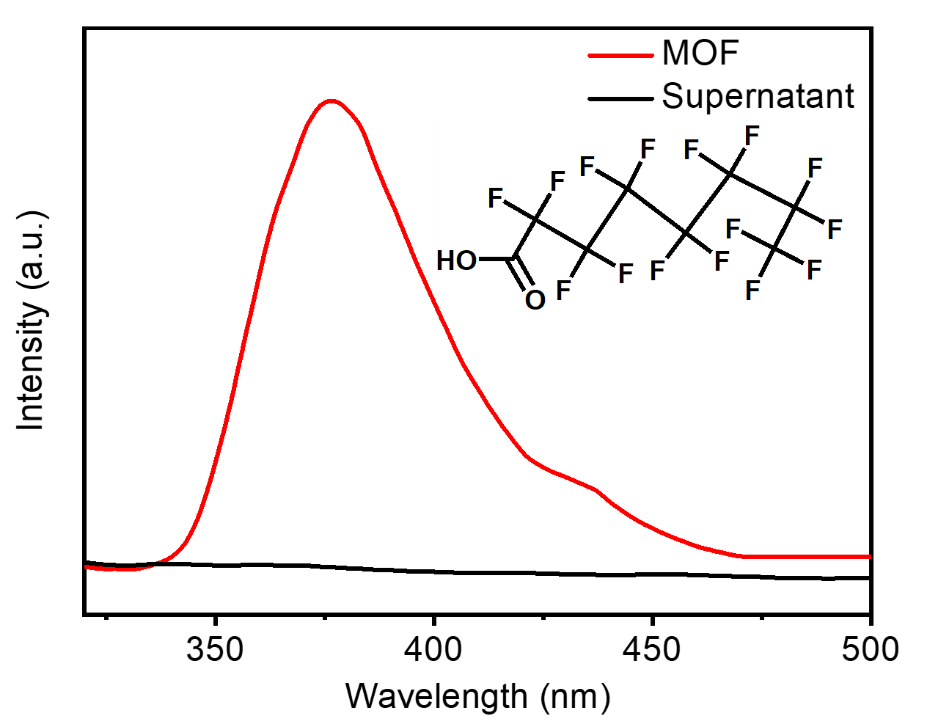
**

**Figure S23.** Emission spectra of ITHD(Zn) and its supernatant after the addition of perfluorooctanoic acid.

**
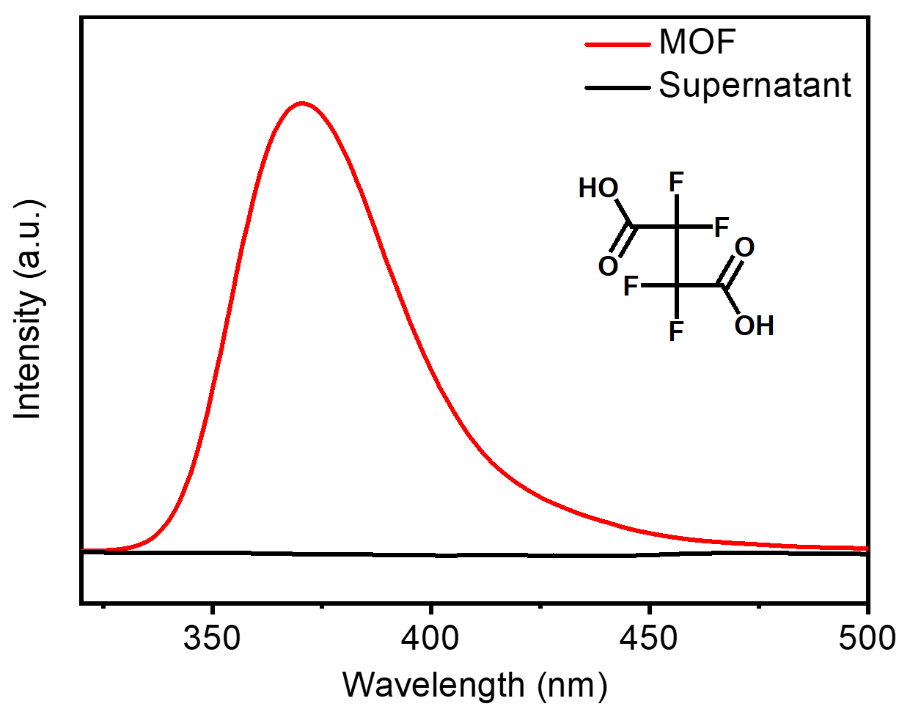
**

**Figure S24.** Emission spectra of ITHD(Zn) and its supernatant after the addition of tetrafluorosuccinic acid.

**
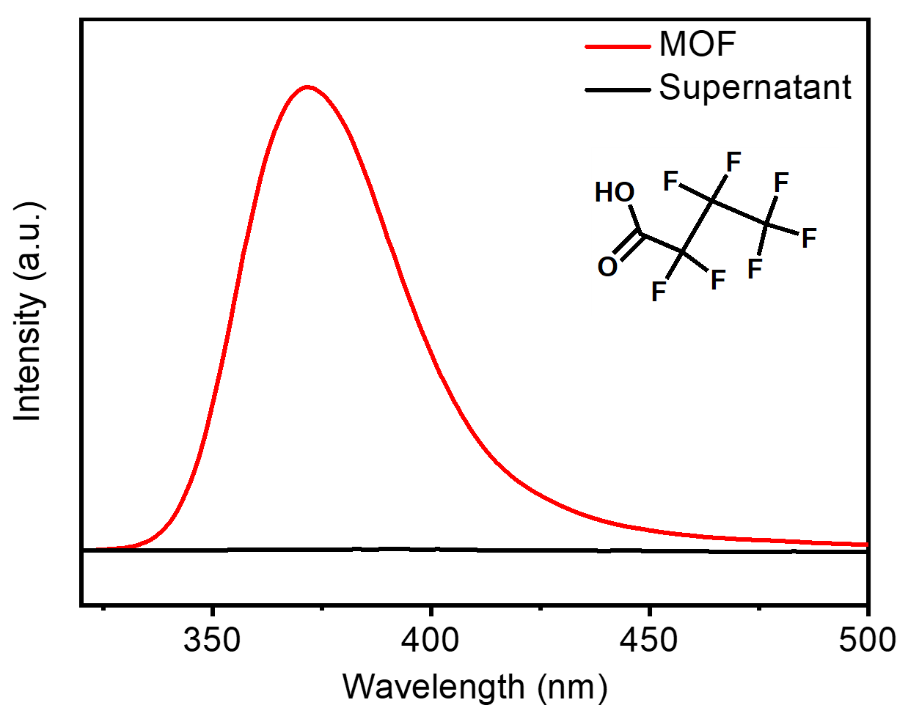
**

**Figure S25.** Emission spectra of ITHD(Zn) and its supernatant after the addition of heptafluorobutyric acid.

**
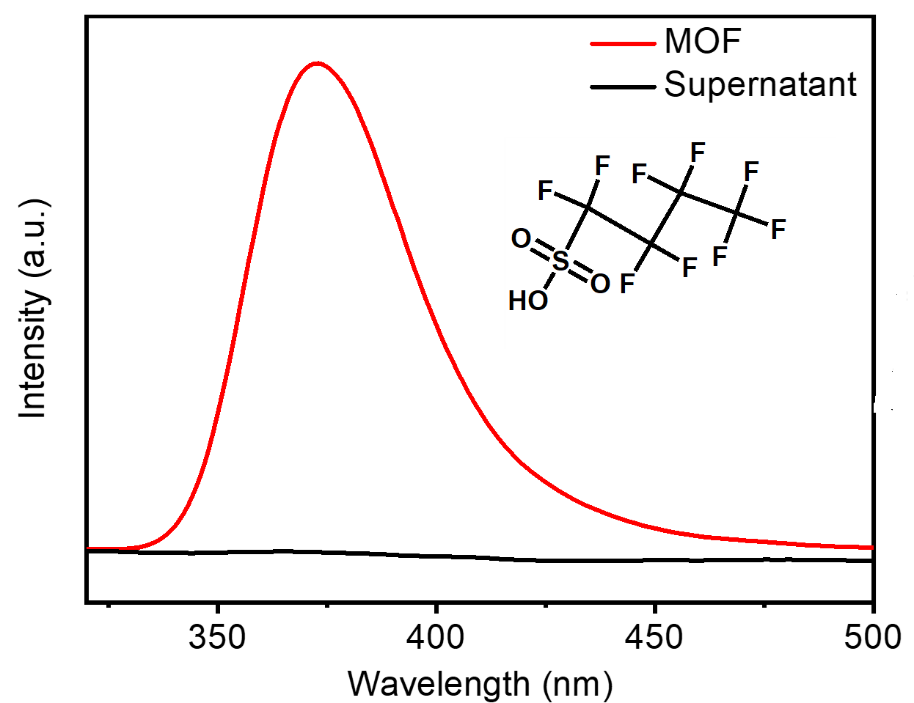
**

**Figure S26.** Emission spectra of ITHD(Zn) and its supernatant after the addition of perfluorobutanesulfonic acid.

**
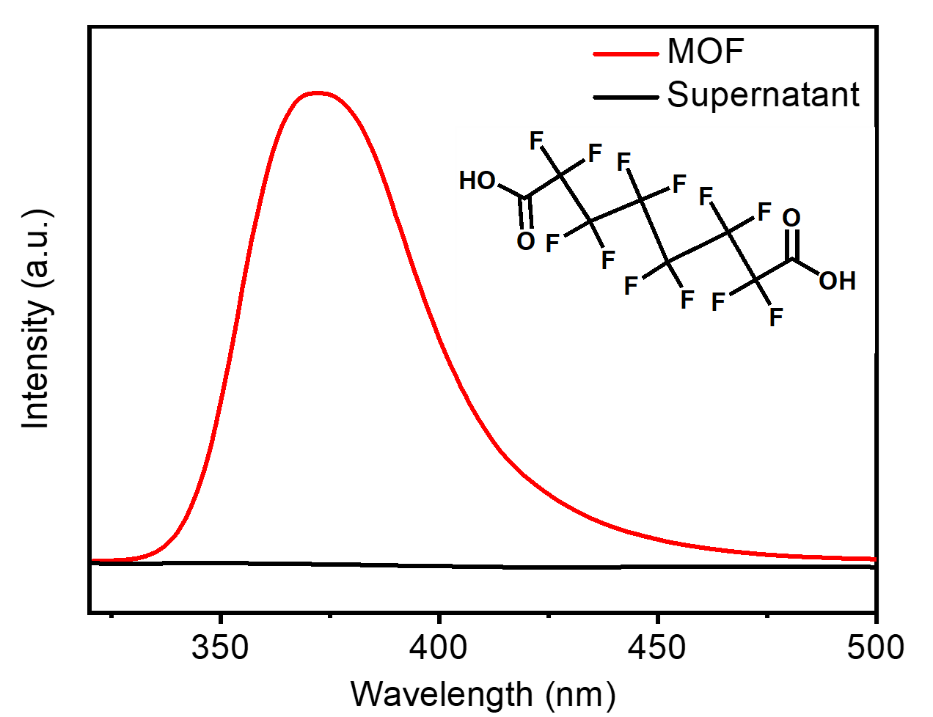
**

**Figure S27.** Emission spectra of ITHD(Zn) and its supernatant after the addition of perfluorosuberic acid.

**
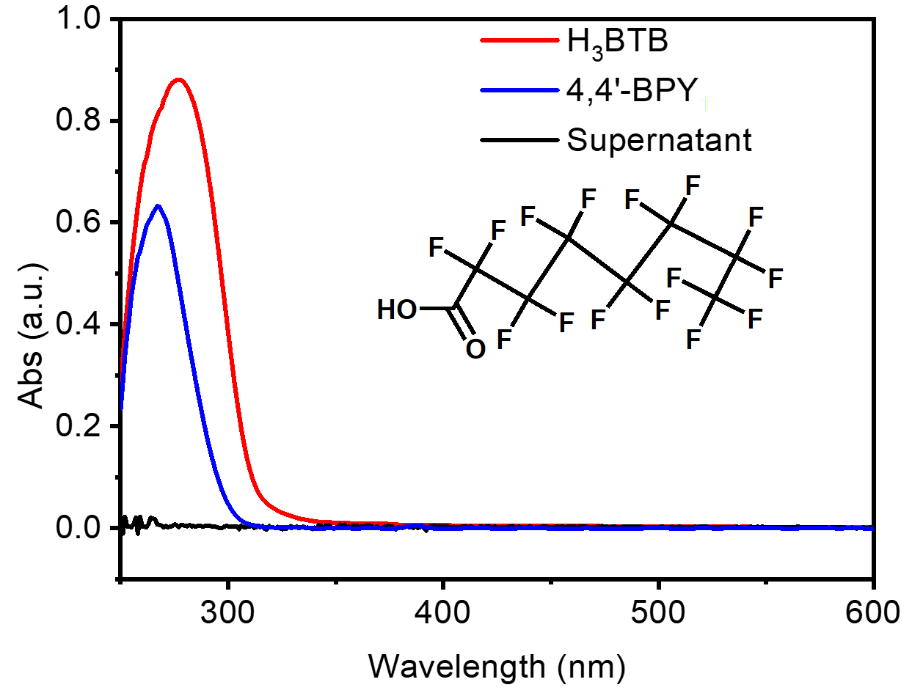
**

**Figure S28.** UV-vis spectra of H_3_BTB, 4,4’-BPY, and the supernatant of ITHD(Zn) after the addition of perfluorooctanoic acid.

**
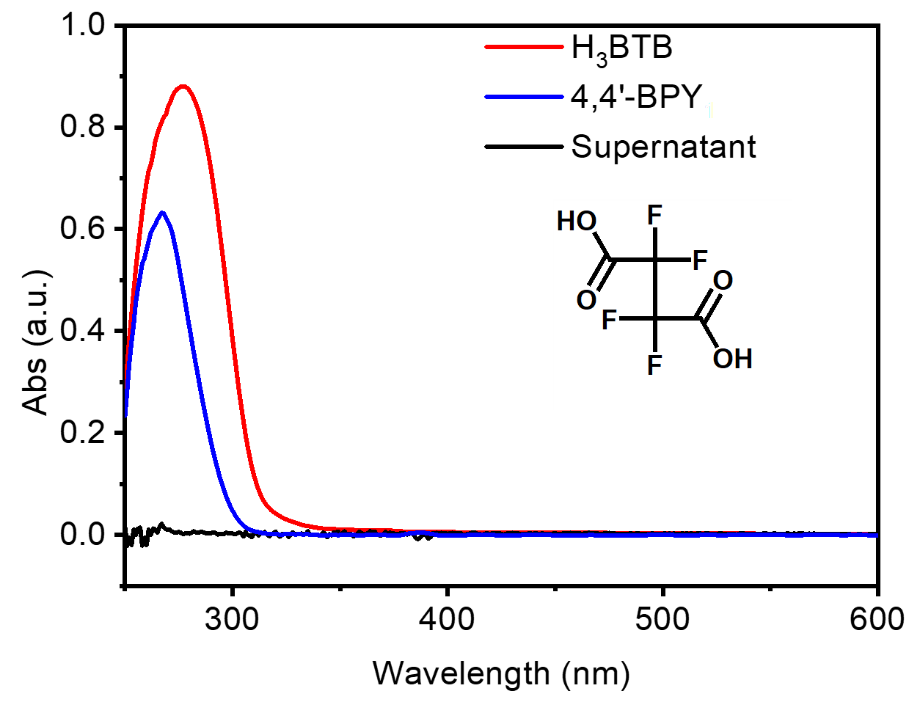
**

**Figure S29.** UV-vis spectra of H_3_BTB, 4,4’-BPY, and the supernatant of ITHD(Zn) after the addition of tetrafluorosuccinic acid.

**
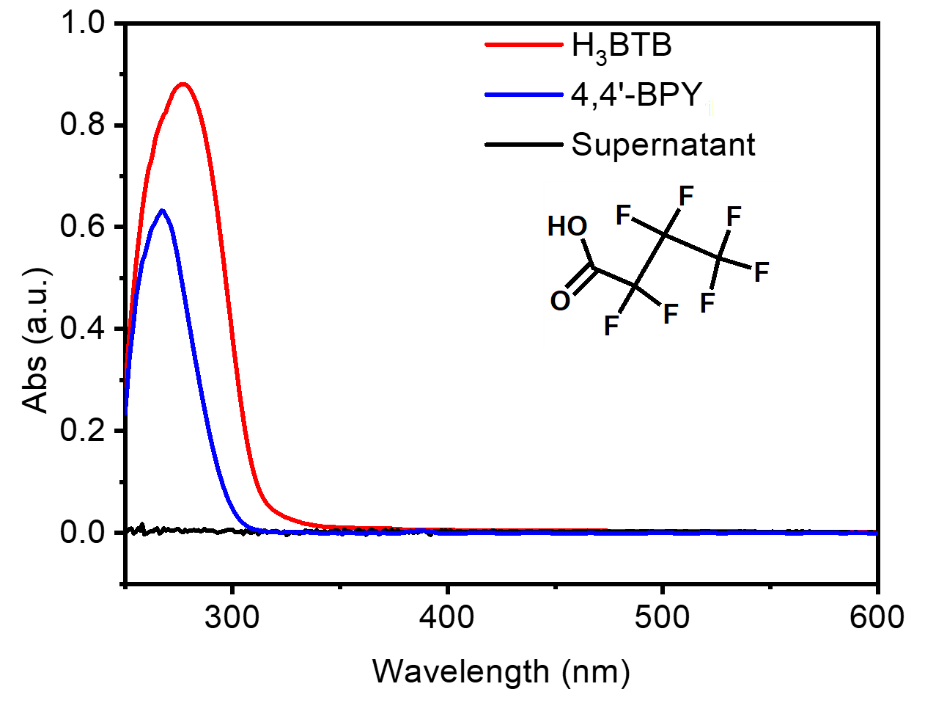
**

**Figure S30.** UV-vis spectra of H_3_BTB, 4,4’-BPY, and the supernatant of ITHD(Zn) after the addition of heptafluorobutyric acid.

**
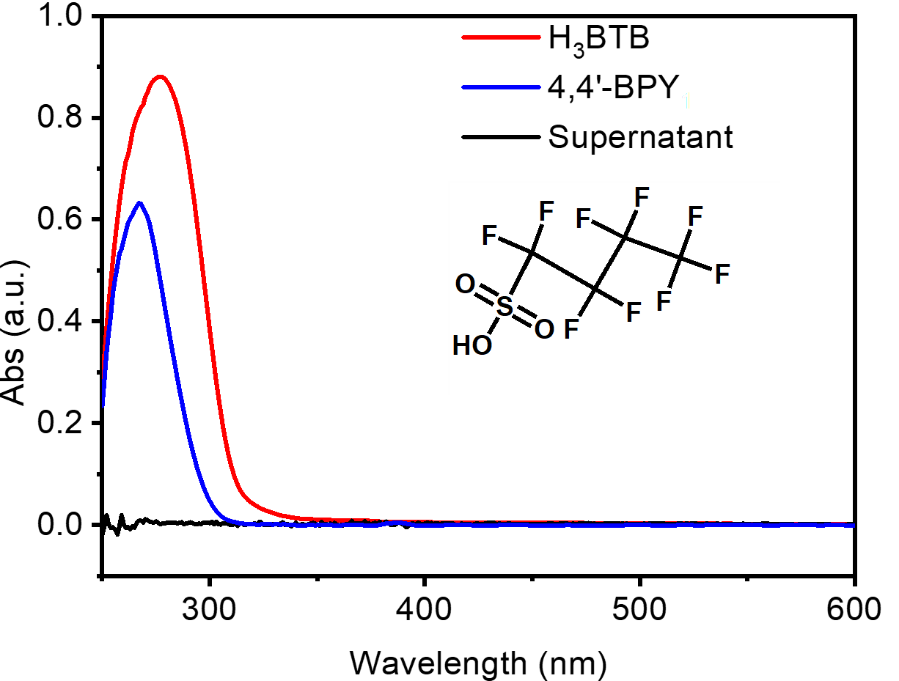
**

**Figure S31.** UV-vis spectra of H_3_BTB, 4,4’-BPY, and the supernatant of ITHD(Zn) after the addition of perfluorobutanesulfonic acid.

**
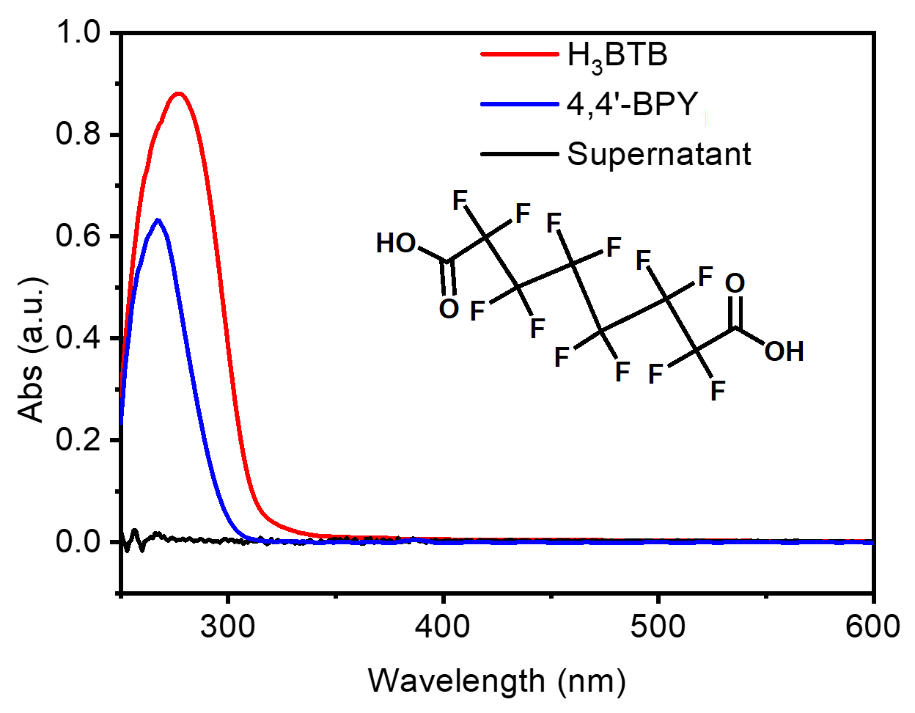
**

**Figure S32.** UV-vis spectra of H_3_BTB, 4,4’-BPY, and the supernatant of ITHD(Zn) after the addition of perfluorosuberic acid.

**
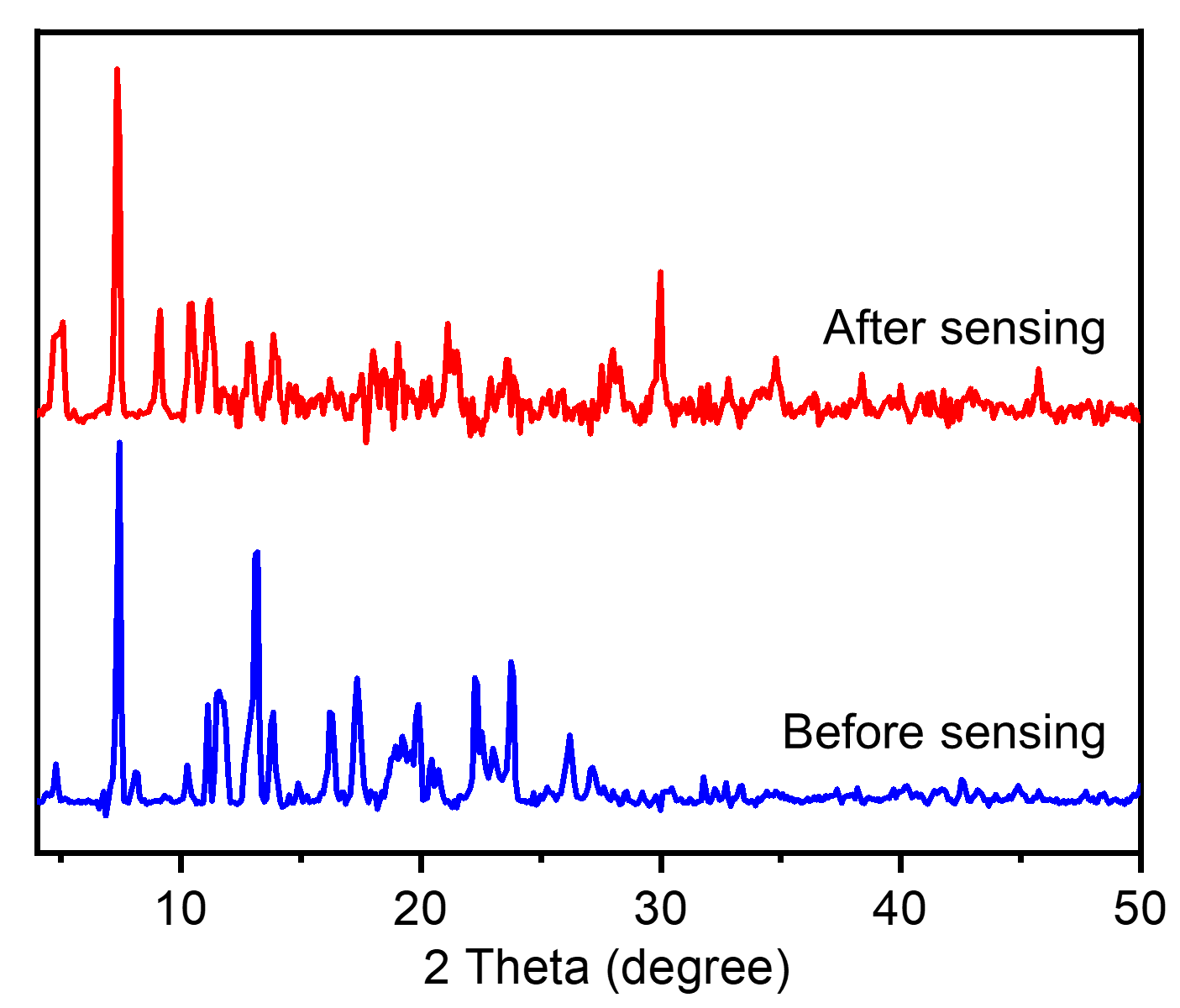
**

**Figure S33.** PXRD patterns of ITHD(Zn) before and after sensing process.

**
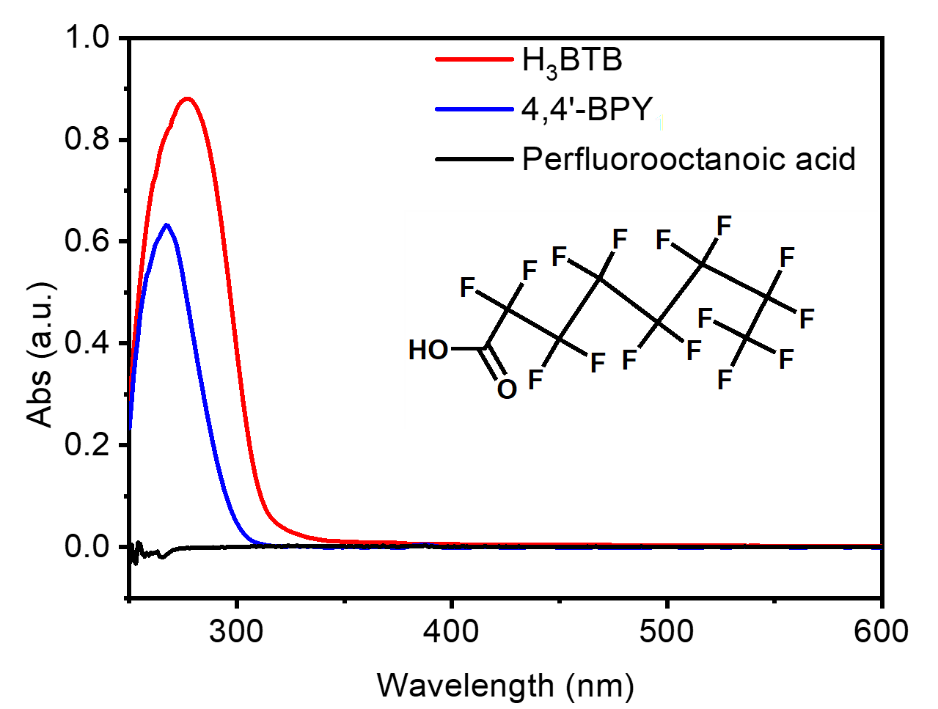
**

**Figure S34.** UV-vis spectra of H_3_BTB, 4,4’-BPY, and perfluorosuberic acid.

**
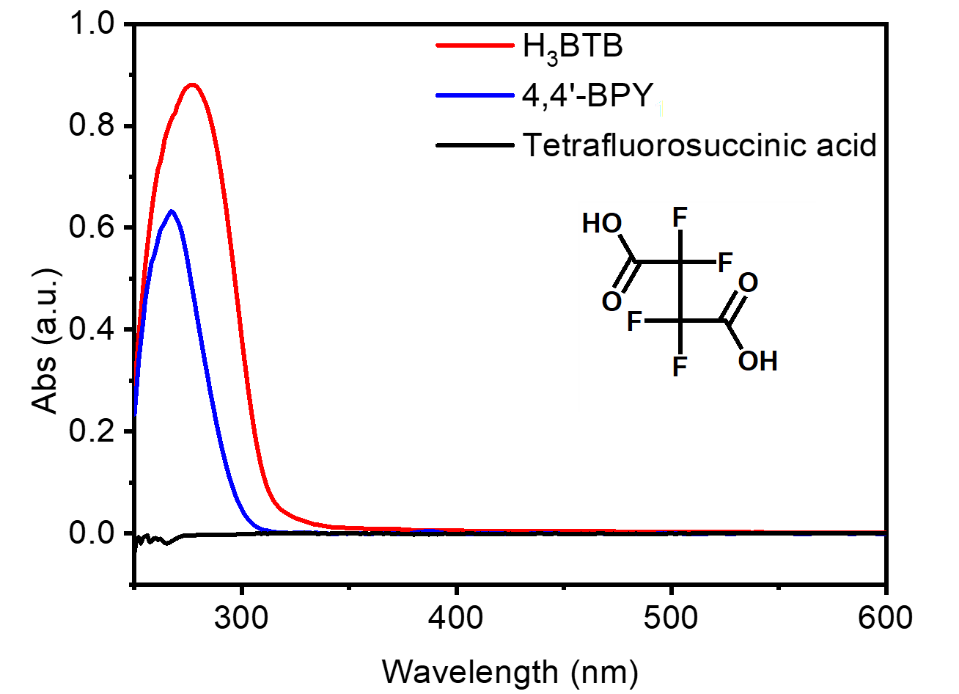
**

**Figure S35.** UV-vis spectra of H_3_BTB, 4,4’-BPY, and tetrafluorosuccinic acid.

**
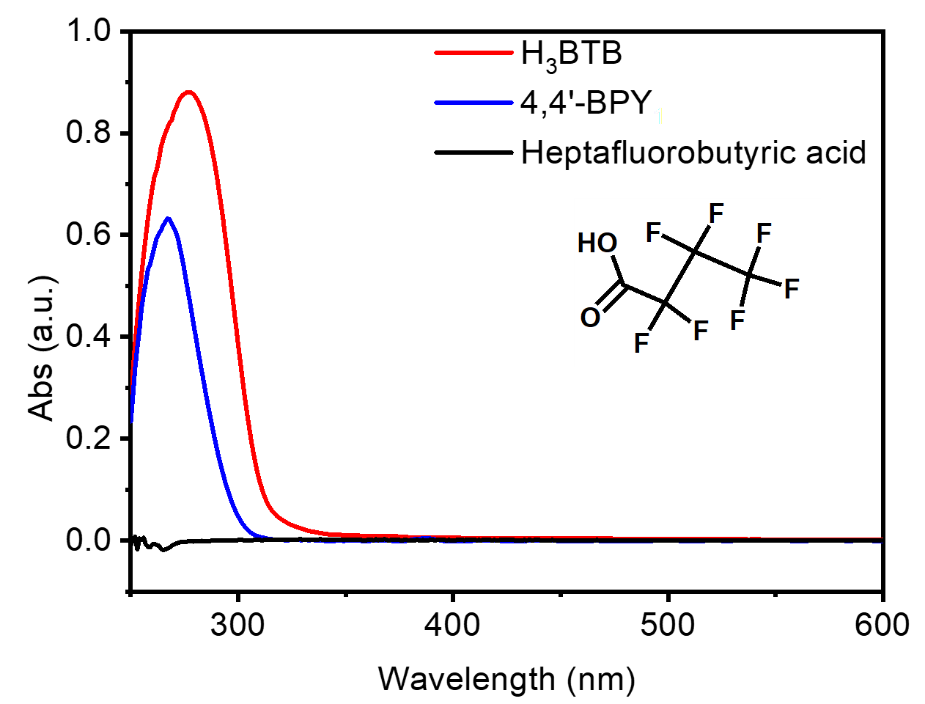
**

**Figure S36.** UV-vis spectra of H_3_BTB, 4,4’-BPY, and heptafluorobutyric acid.

**
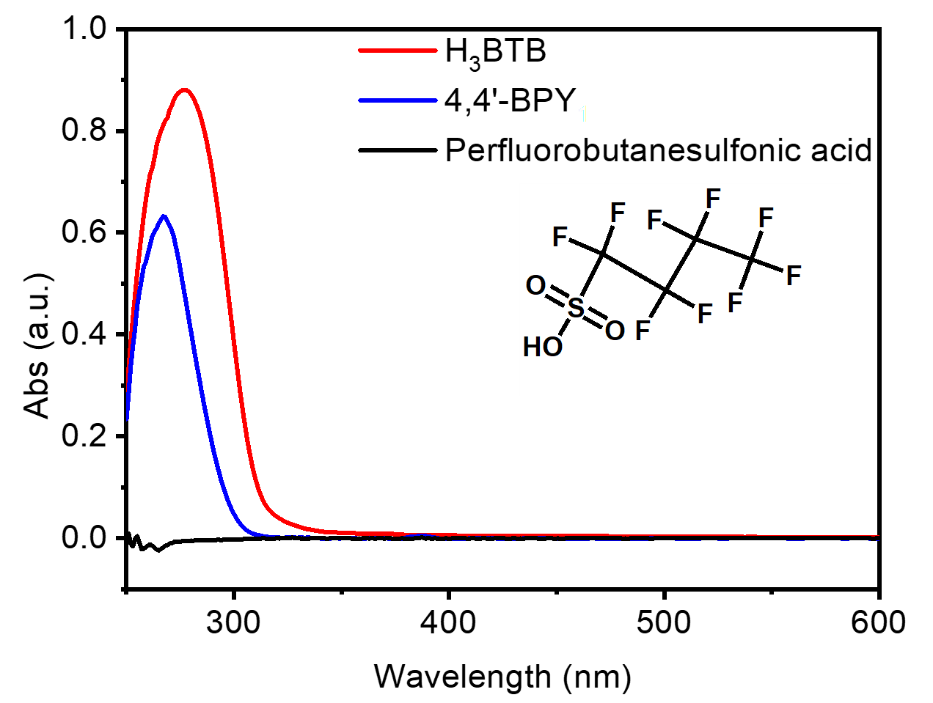
**

**Figure S37.** UV-vis spectra of H_3_BTB, 4,4’-BPY, and perfluorobutanesulfonic acid.

**
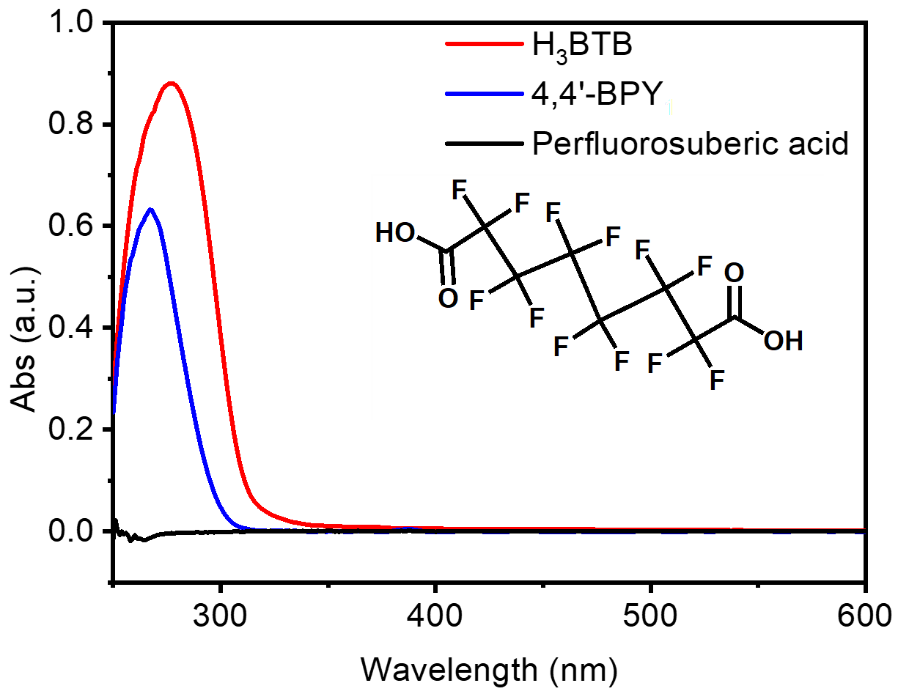
**

**Figure S38.** UV-vis spectra of H_3_BTB, 4,4’-BPY, and perfluorosuberic acid.

**
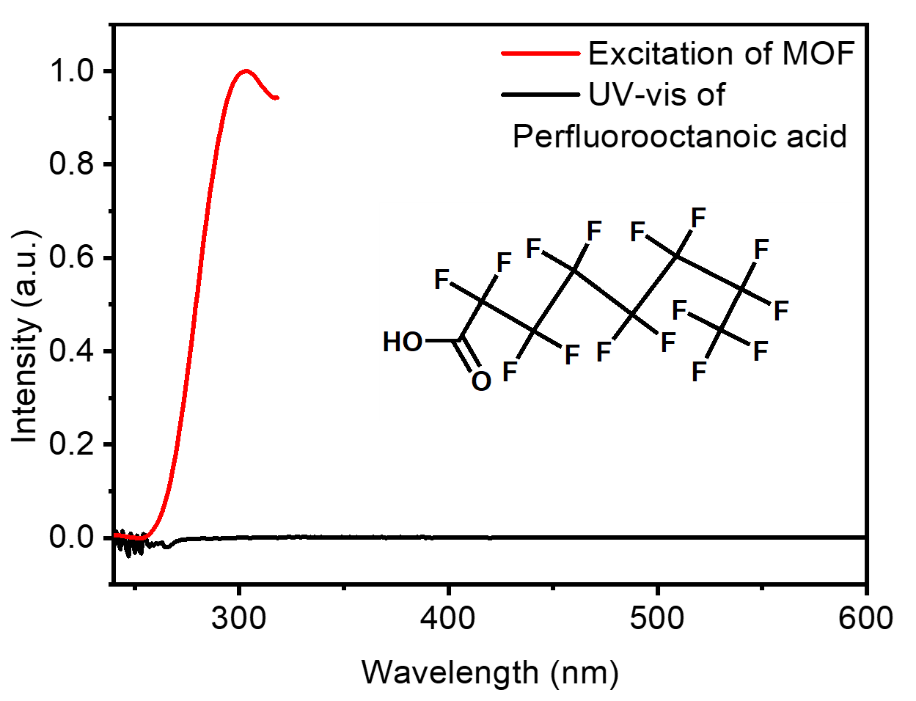
**

**Figure S39.** UV-vis spectrum of perfluorooctanoic acid and excitation spectrum of ITHD(Zn).

**
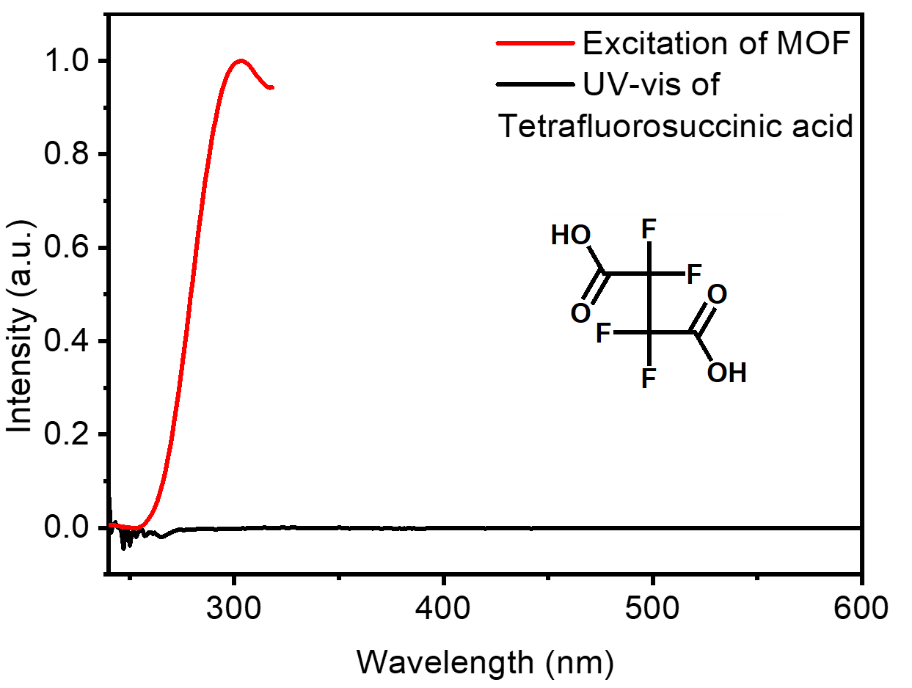
**

**Figure S40.** UV-vis spectrum of tetrafluorosuccinic acid and excitation spectrum of ITHD(Zn).

**
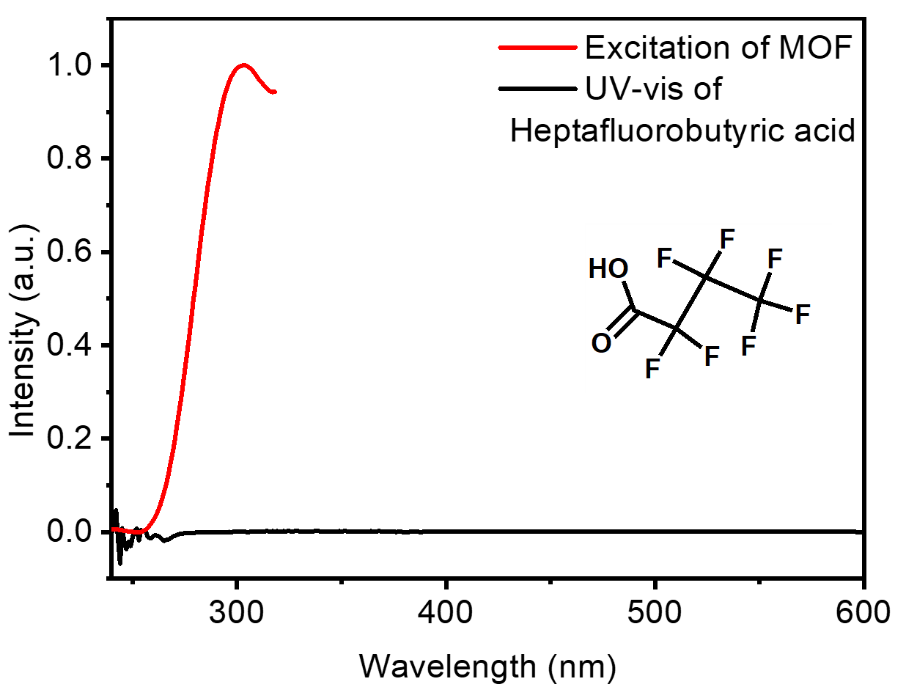
**

**Figure S41.** UV-vis spectrum of heptafluorobutyric acid and excitation spectrum of ITHD(Zn).

**
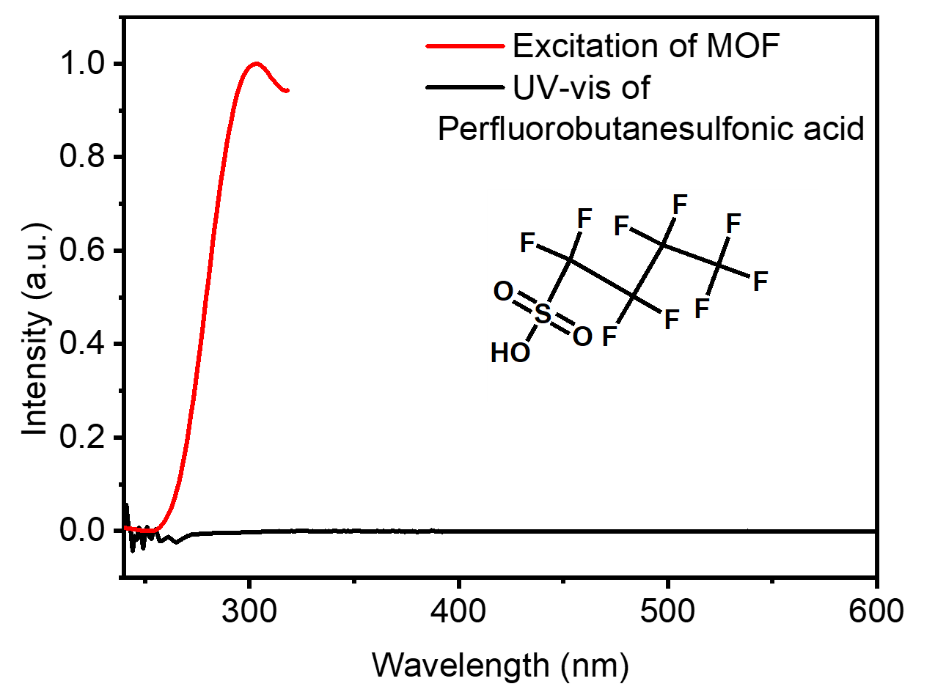
**

**Figure S42.** UV-vis spectrum of perfluorobutanesulfonic acid and excitation spectrum of ITHD(Zn).

**
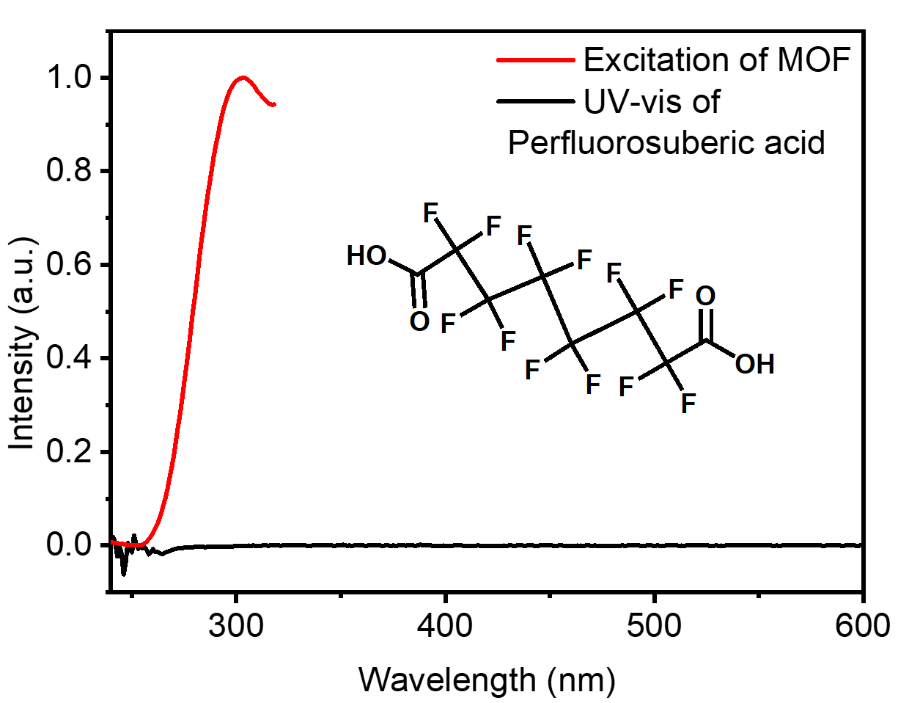
**

**Figure S43.** UV-vis spectrum of perfluorosuberic acid and excitation spectrum of ITHD(Zn).

**
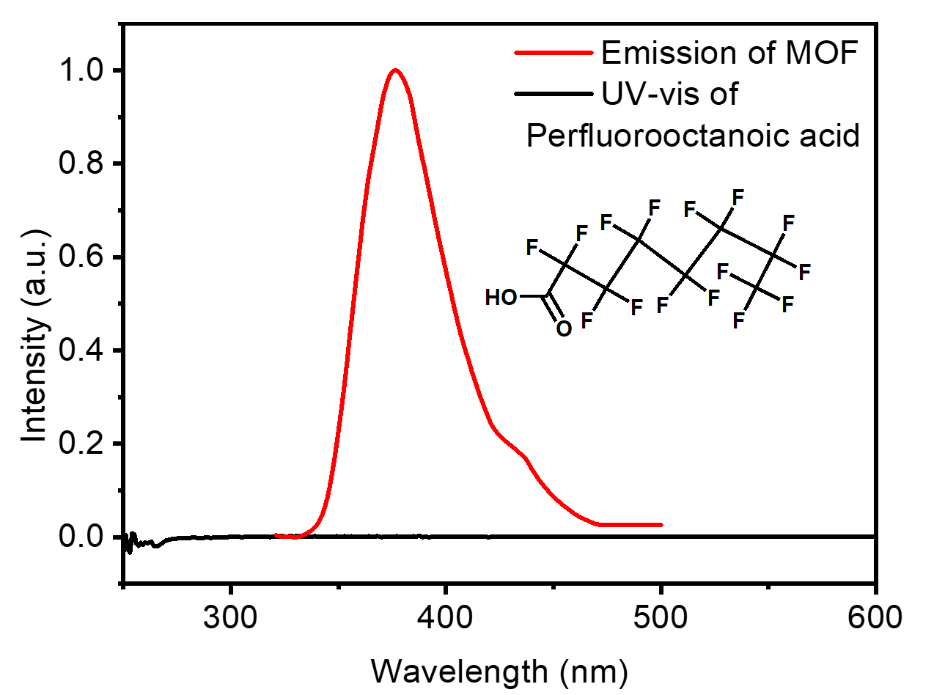
**

**Figure S44.** UV-vis spectrum of perfluorooctanoic acid and emission spectrum of ITHD(Zn).

**
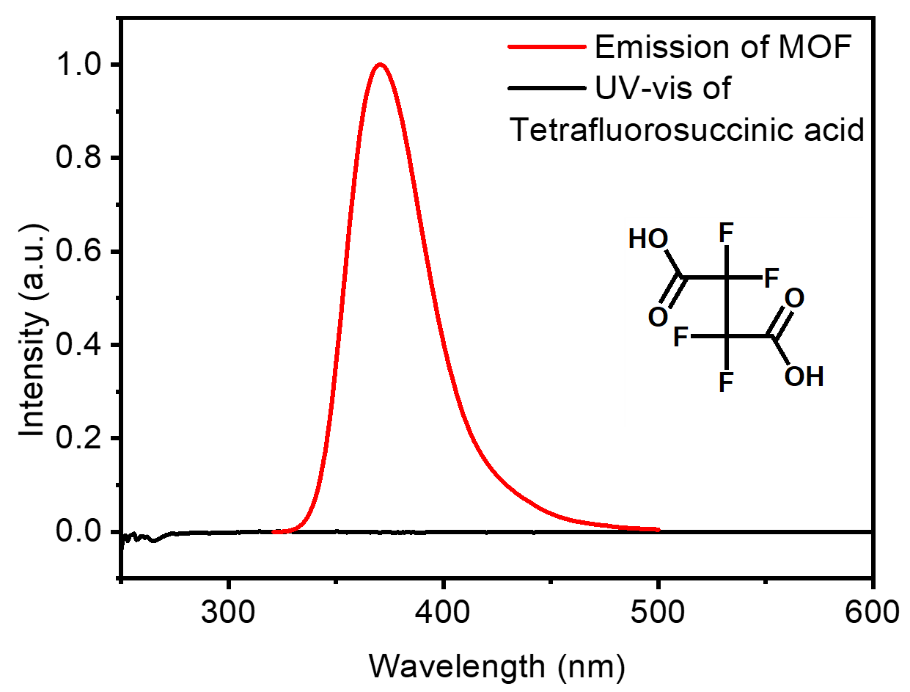
**

**Figure S45.** UV-vis spectrum of tetrafluorosuccinic acid and emission spectrum of ITHD(Zn).

**
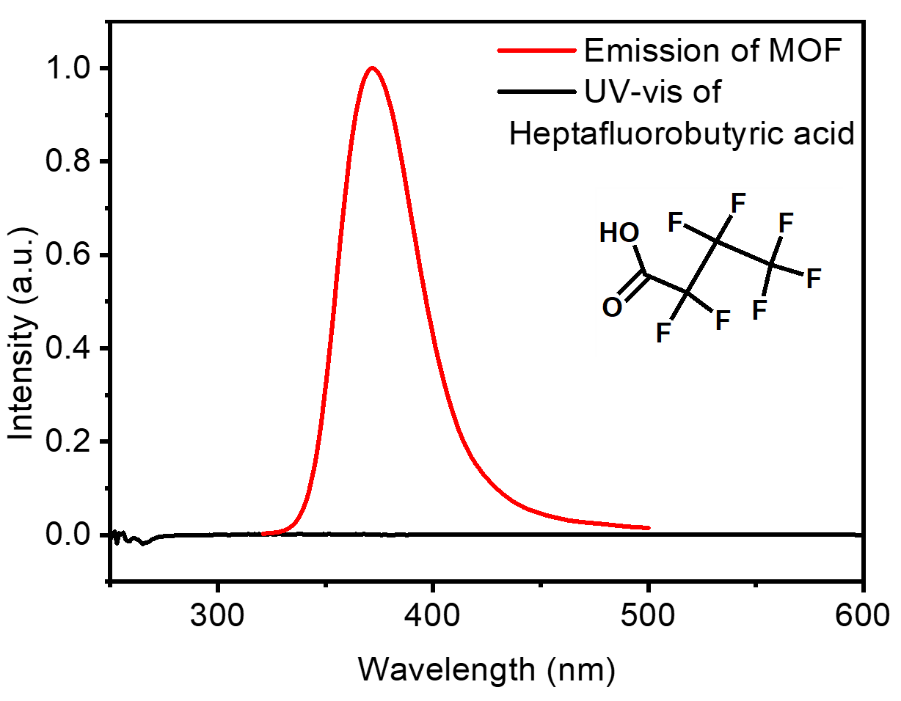
**

**Figure S46.** UV-vis spectrum of heptafluorobutyric acid and emission spectrum of ITHD(Zn).

**
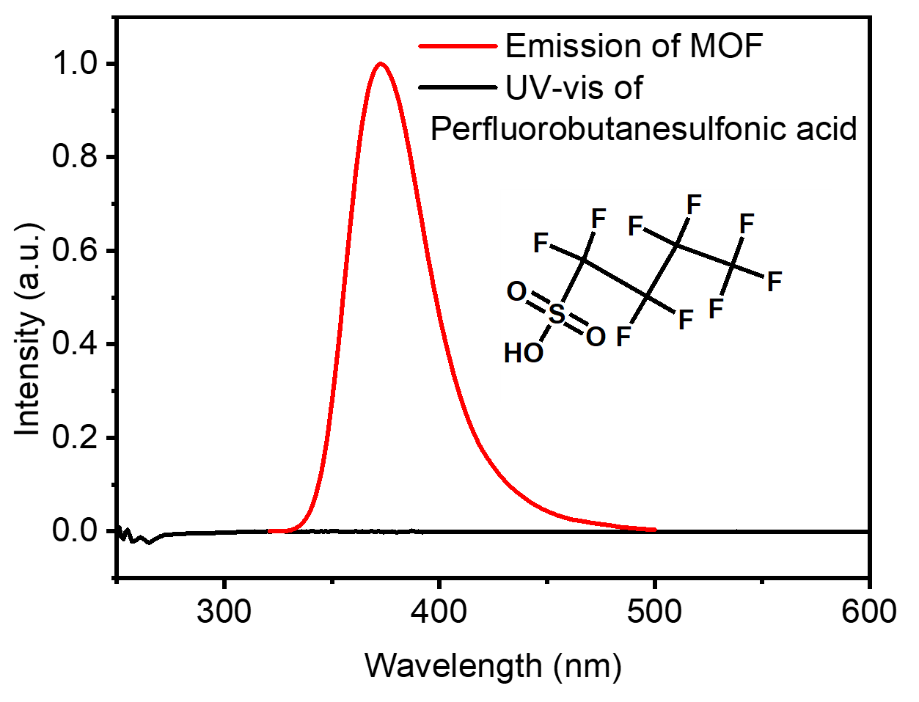
**

**Figure S47.** UV-vis spectrum of perfluorobutanesulfonic acid and emission spectrum of ITHD(Zn).

**
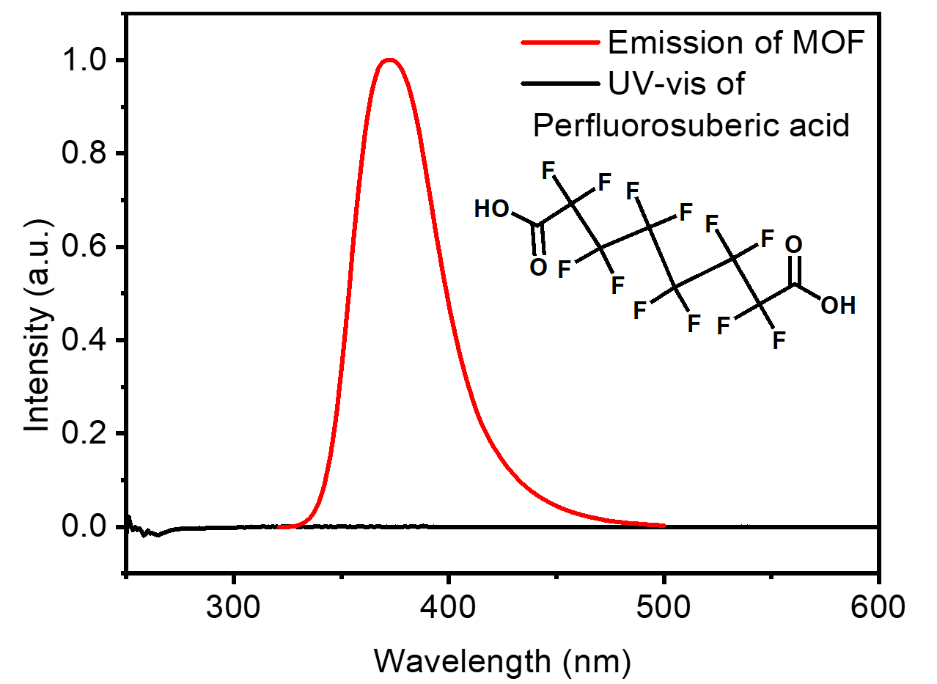
**

**Figure S48.** UV-vis spectrum of perfluorosuberic acid and emission spectrum of ITHD(Zn).

**
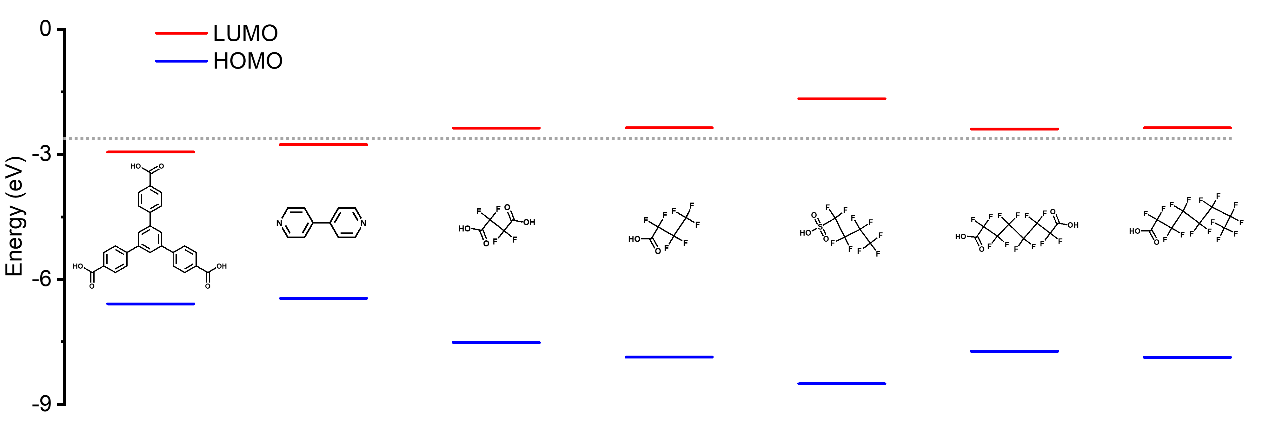
**

**Figure S49.** HOMO-LUMO energy levels of the ligands and the analytes.

**
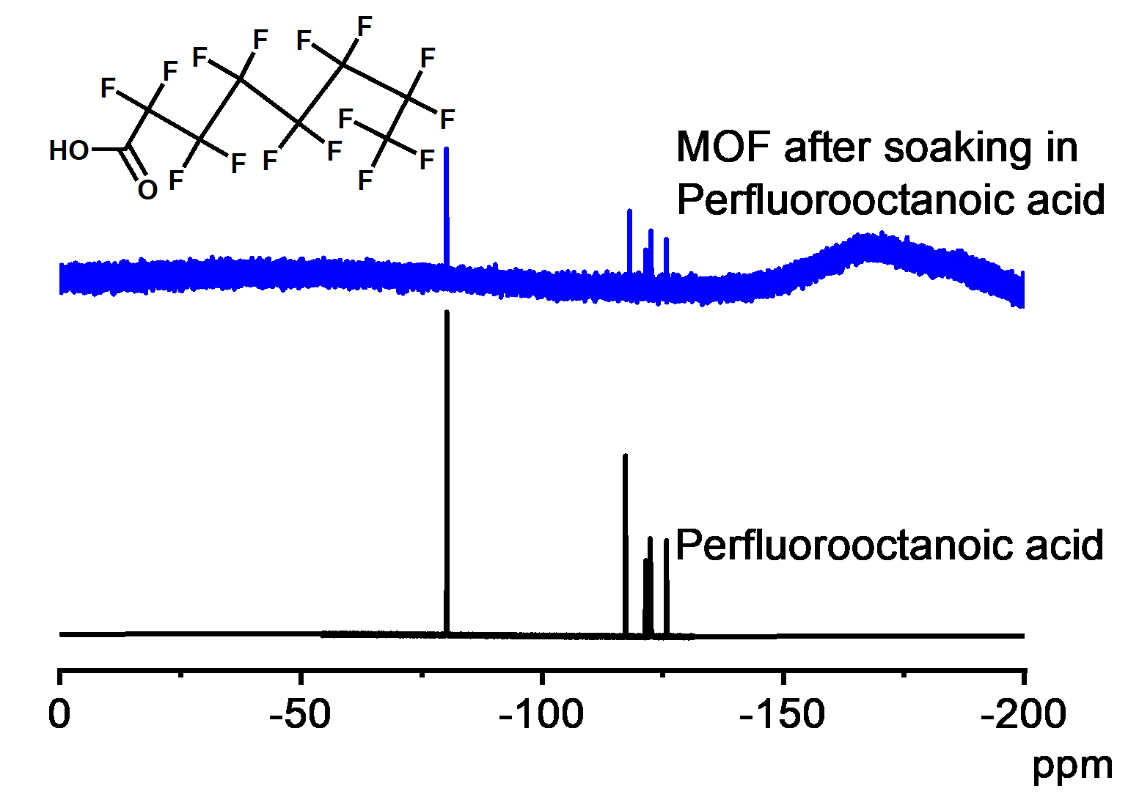
**

**Figure S50.** Liquid ^19^F NMR spectra of perfluorooctanoic acid and ITHD(Zn) after soaking in perfluorooctanoic acid.

**
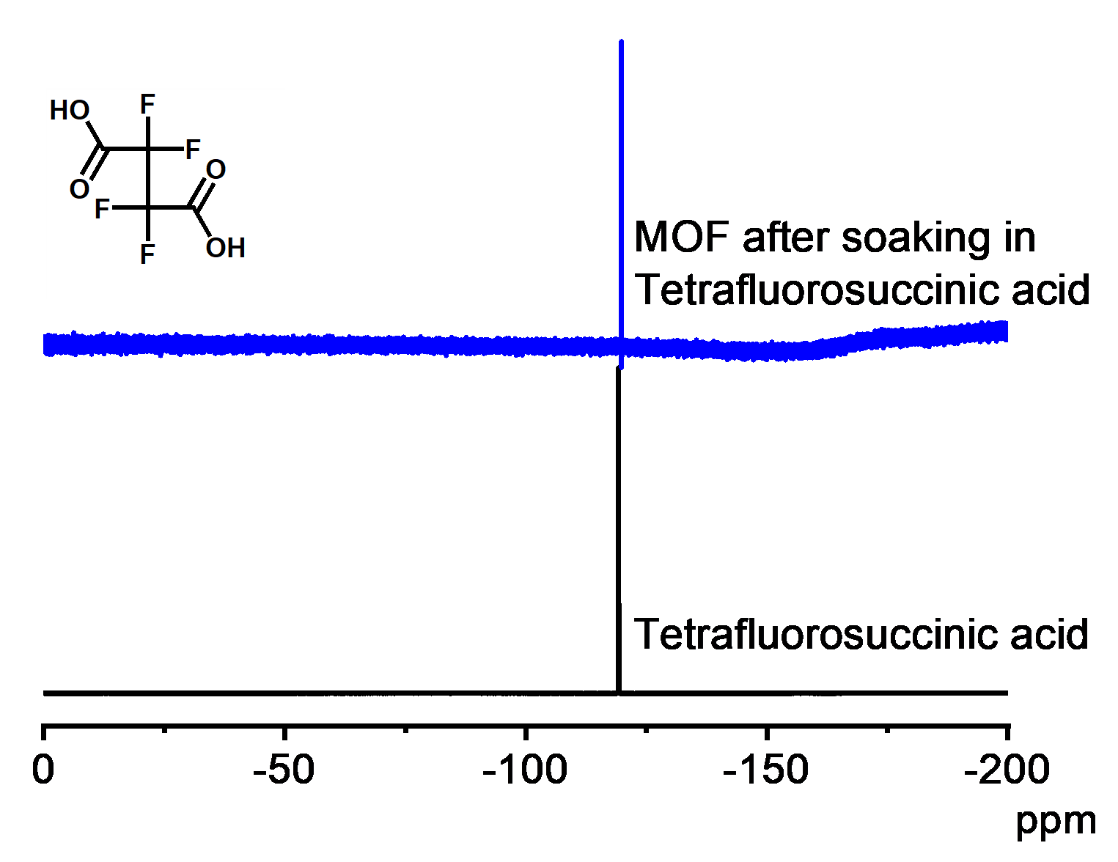
**

**Figure S51.** Liquid ^19^F NMR spectra of tetrafluorosuccinic acid and ITHD(Zn) after soaking in tetrafluorosuccinic acid.

**
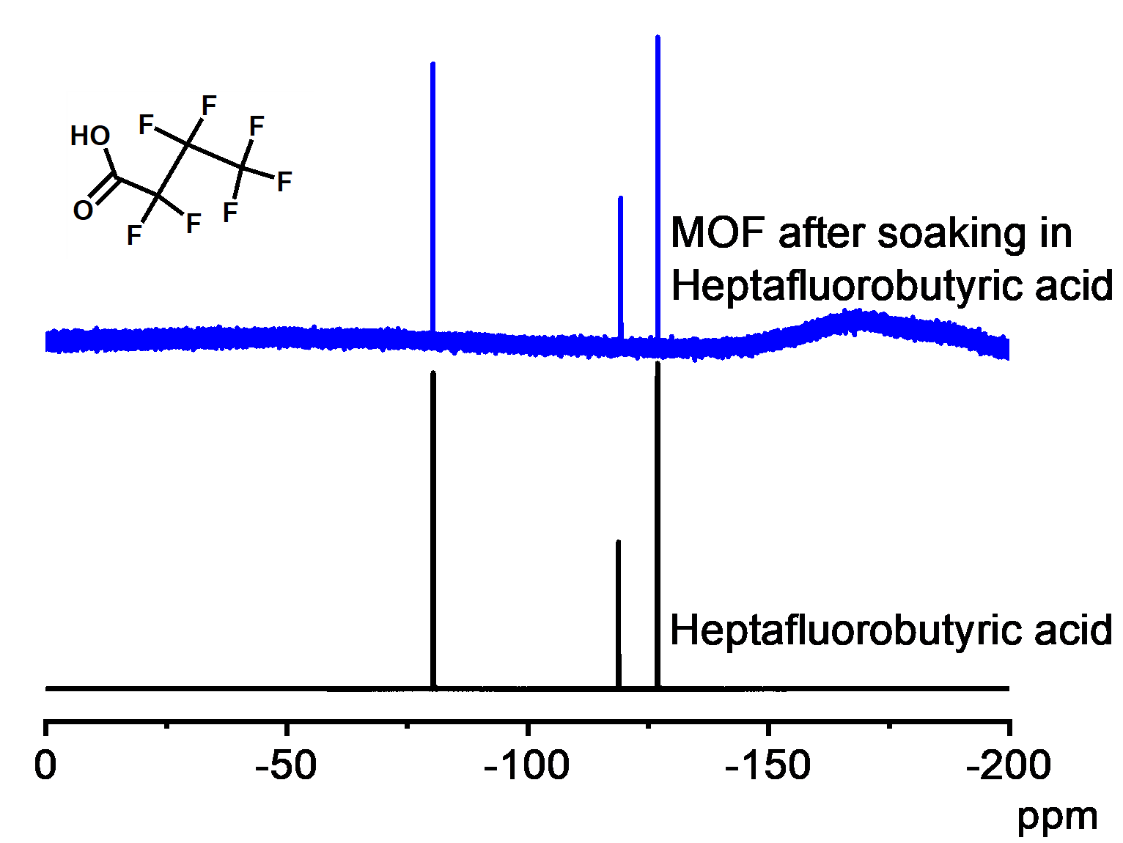
**

**Figure S52.** Liquid ^19^F NMR spectra of heptafluorobutyric acid and ITHD(Zn) after soaking in heptafluorobutyric acid.

**
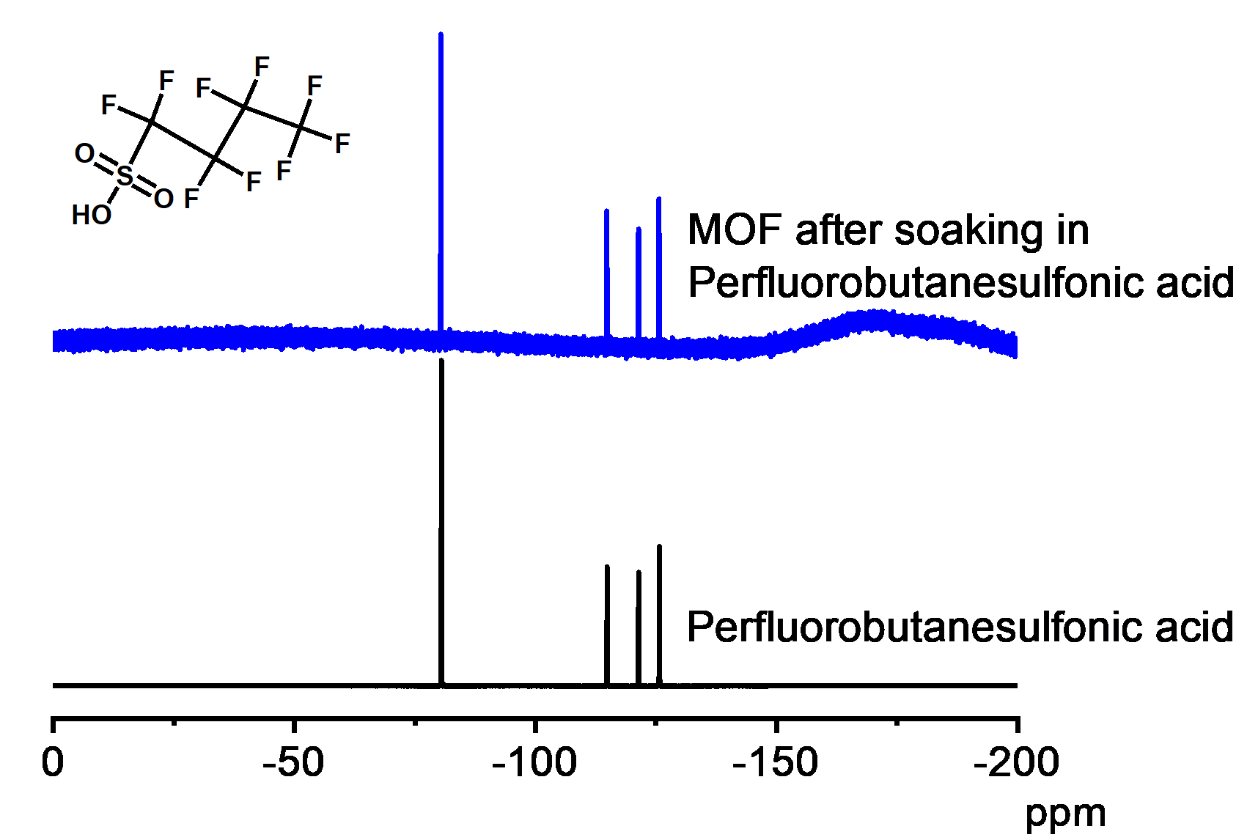
**

**Figure S53.** Liquid ^19^F NMR spectra of perfluorobutanesulfonic acid and ITHD(Zn) after soaking in perfluorobutanesulfonic acid.

**
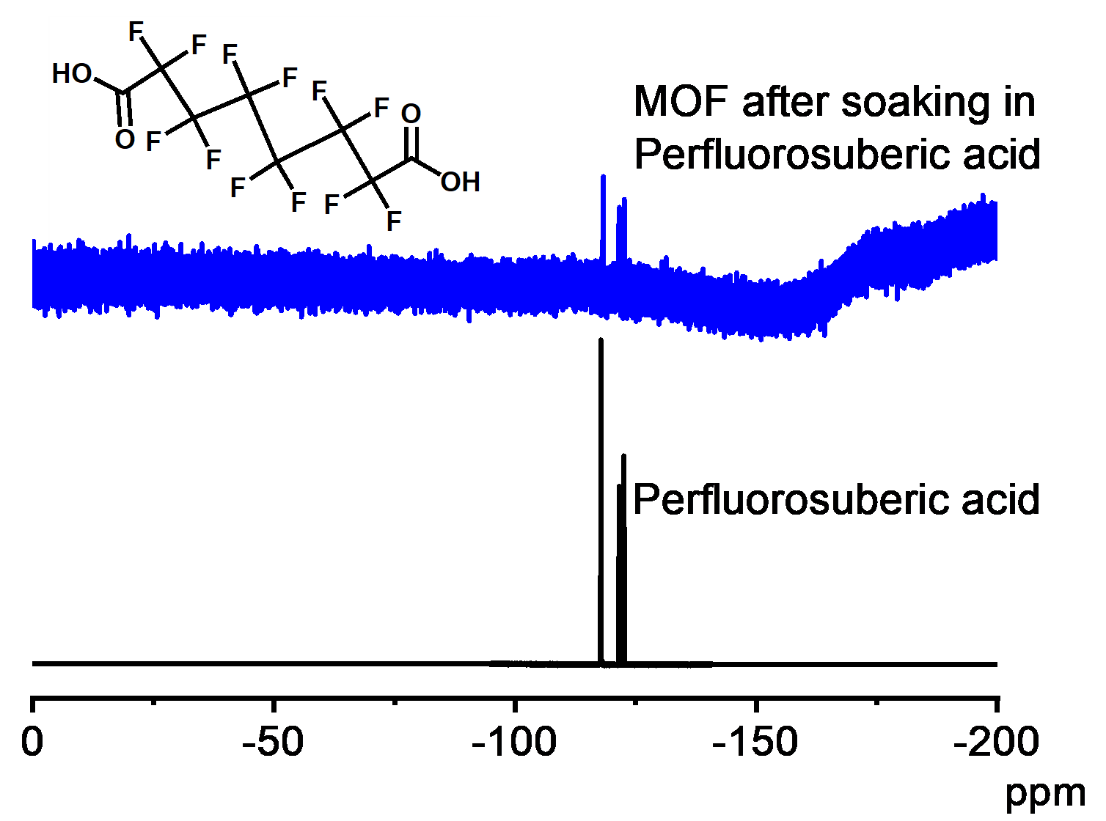
**

**Figure S54.** Liquid ^19^F NMR spectra of perfluorosuberic acid and ITHD(Zn) after soaking in perfluorosuberic acid.


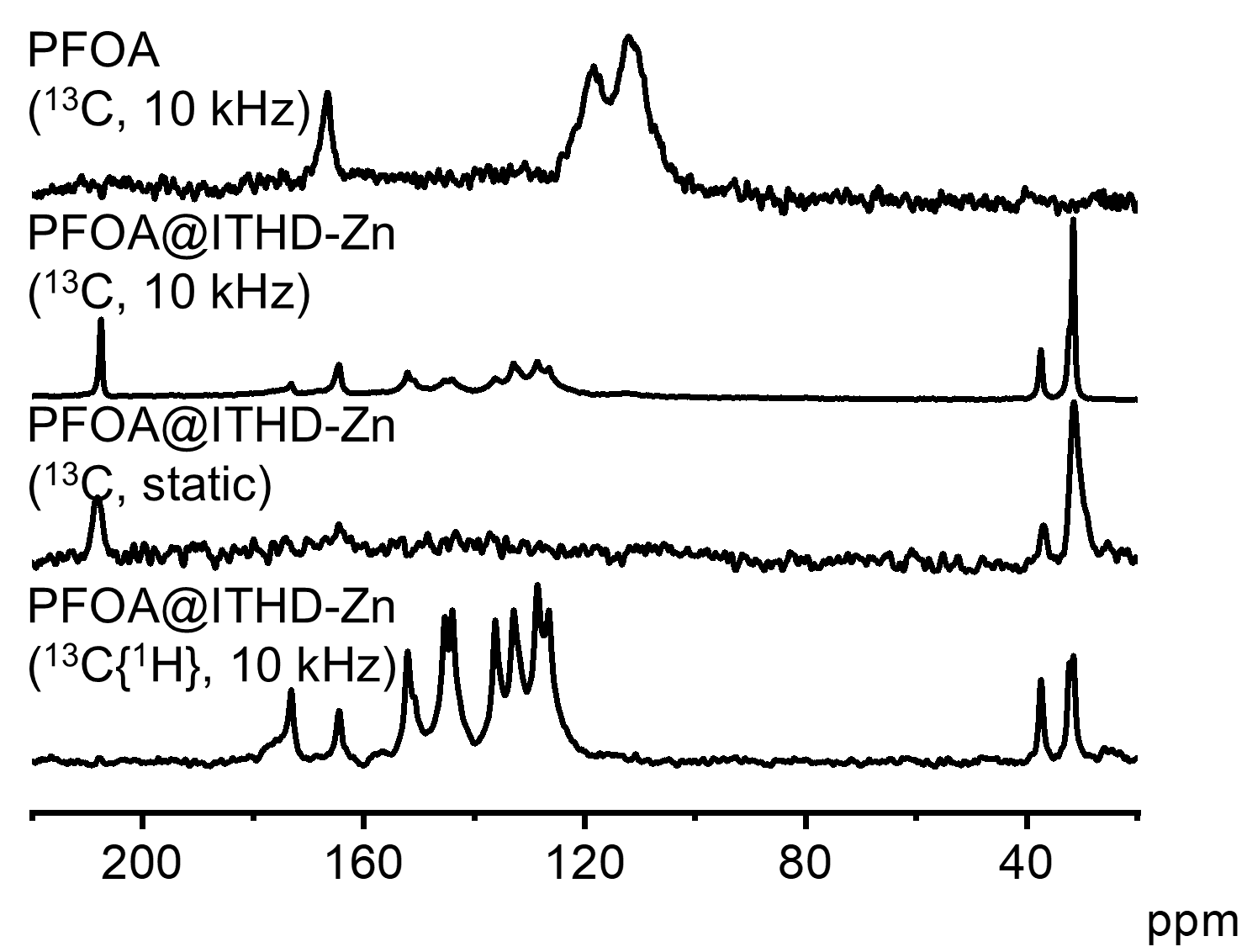


**Figure S55.** The ^13^C{^1^H} MAS NMR spectra recorded with direct excitation of ^13^C nuclei for pure PFOA (10 kHz), PFOA@ITHD(Zn) (10 kHz), and PFOA@ITHD(Zn) (static), and the ^13^C{^1^H} MAS NMR spectra of PFOA@ITHD(Zn) (10 kHz, CP at a contact time of 3.0 ms).


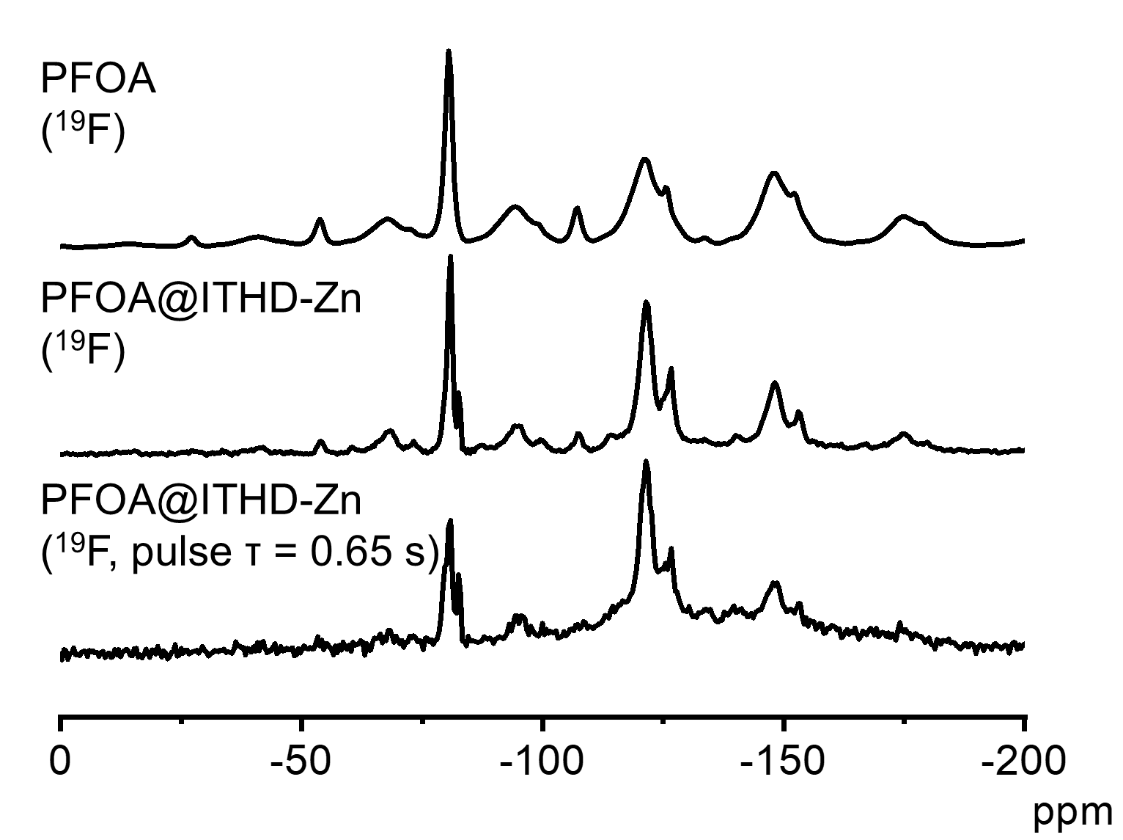


**Figure S56.** The ^19^F MAS NMR spectra recorded for PFOA (10 kHz), PFOA@ITHD(Zn) (10 kHz), and PFOA@ITHD(Zn) obtained by the inversion-recovery 180°-τ-90° pulse sequence at τ = 0.65 s (10 kHz).


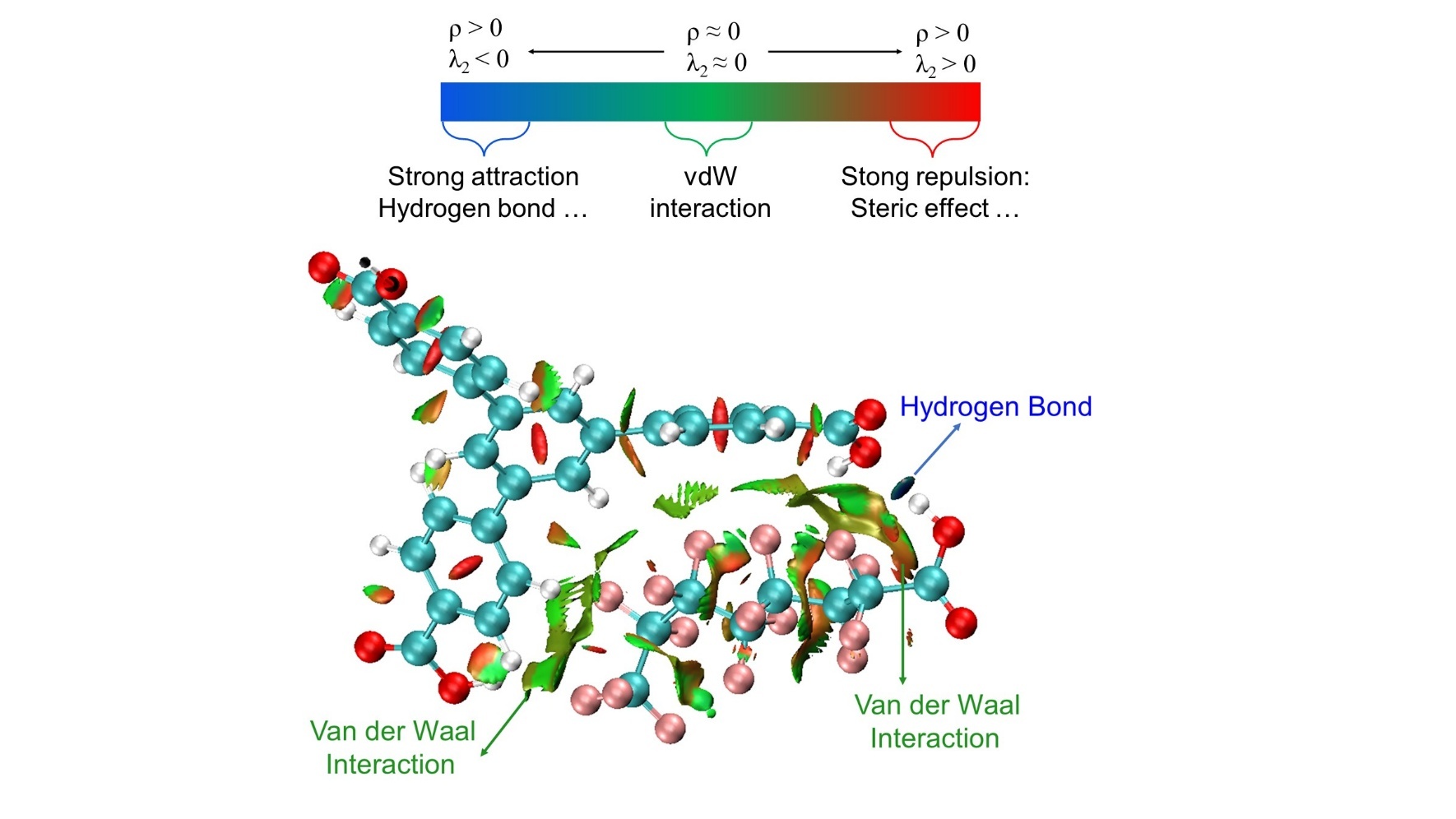


**Figure S57.** RDG isosurface map of H_3_BTB and perfluorooctanoic acid, mapped with sign(λ₂)ρ. Green isosurfaces indicate van der Waals interactions, while blue regions correspond to hydrogen bonding between the carboxyl group of PFOA and the hydrogen atoms on the H_3_BTB phenyl rings. Atom code: C, cyan; O, red; H, white; F, pink.


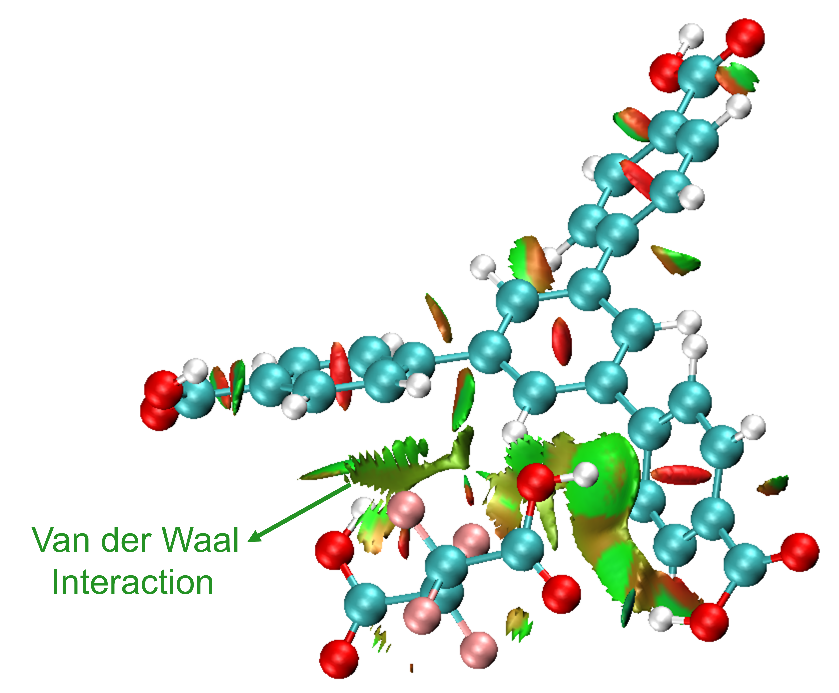


**Figure S58.** RDG isosurface map of H_3_BTB and tetrafluorosuccinic acid. Atom code: C, cyan; O, red; H, white; F, pink.


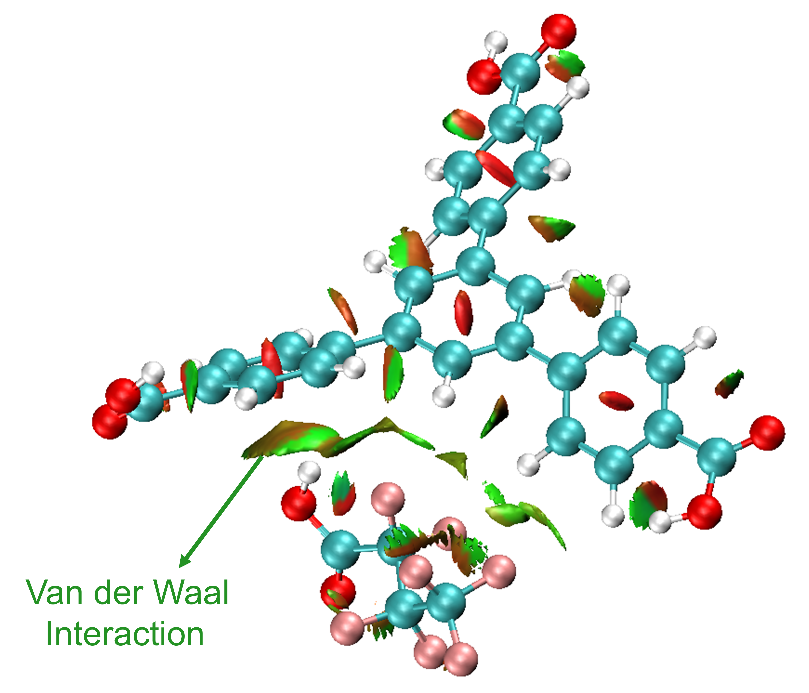


**Figure S59.** RDG isosurface map of H_3_BTB and heptafluorobutyric acid. Atom code: C, cyan; O, red; H, white; F, pink.


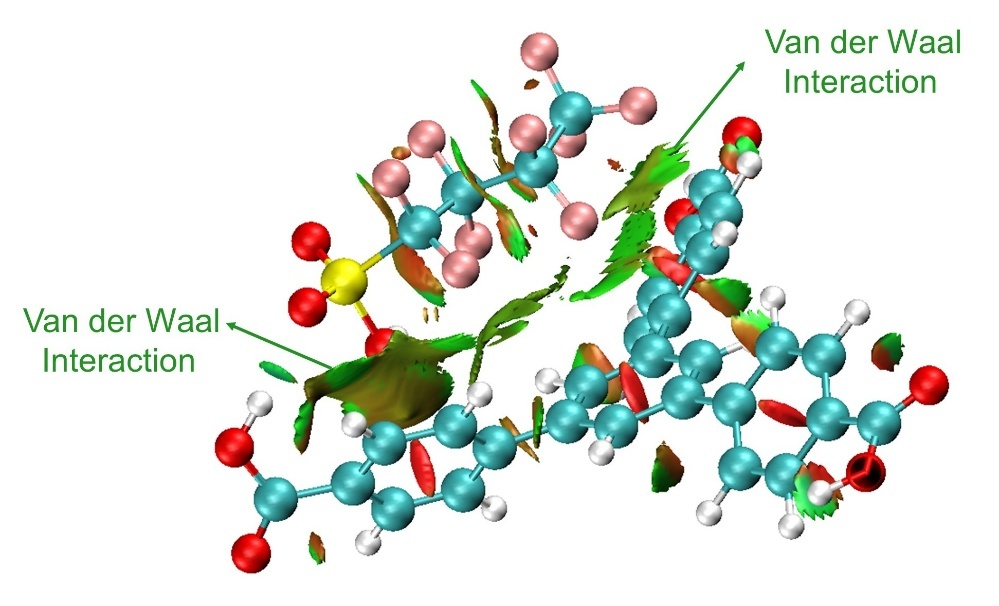


**Figure S60.** RDG isosurface map of H_3_BTB and perfluorobutanesulfonic acid. Atom code: C, cyan; O, red; H, white; F, pink; S, yellow.


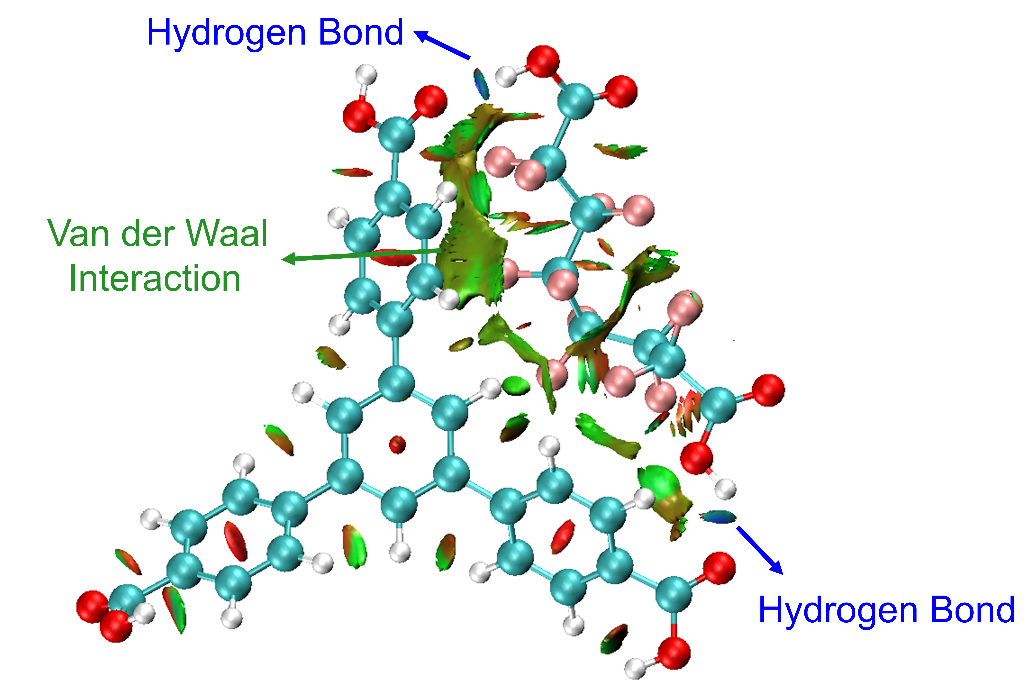


**Figure S61.** RDG isosurface map of H_3_BTB and perfluorobutanesulfonic acid. Atom code: C, cyan; O, red; H, white; F, pink.


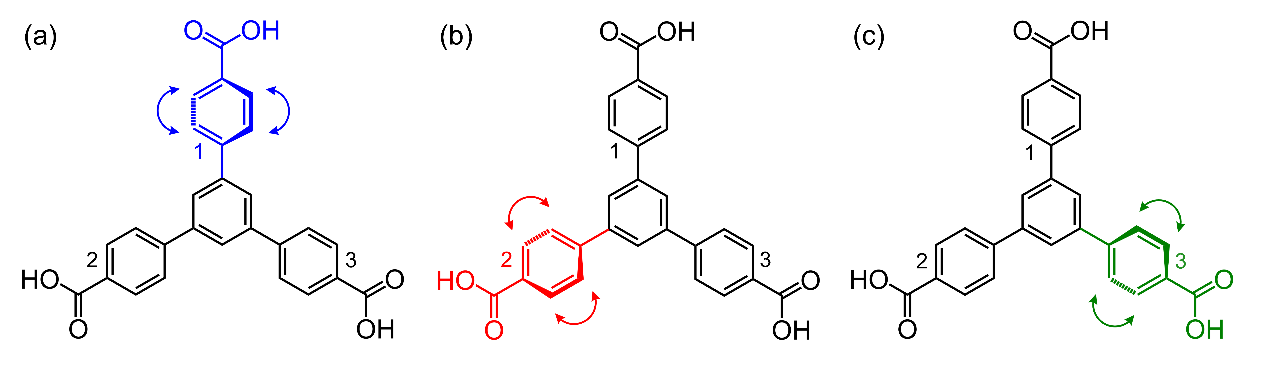


**Figure S62.** Out of plane vibration modes of H_3_BTB.


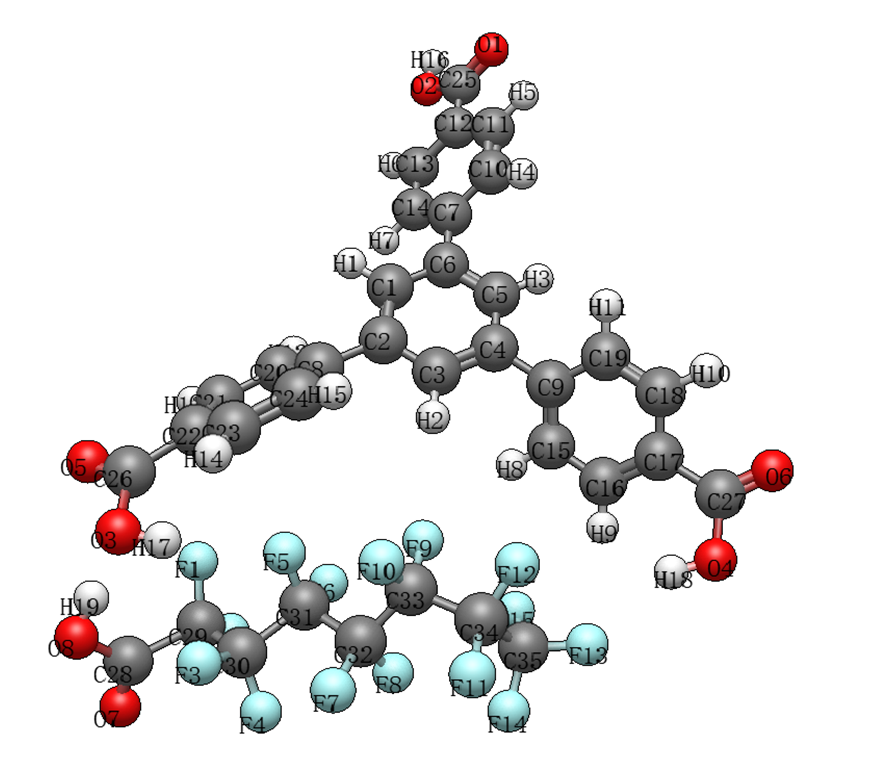


**Figure S63.** Vibration displacement calculation model of H_3_BTB with perfluorooctanoic acid. Atom code: C, grey; O, red; H, white; F, celeste.


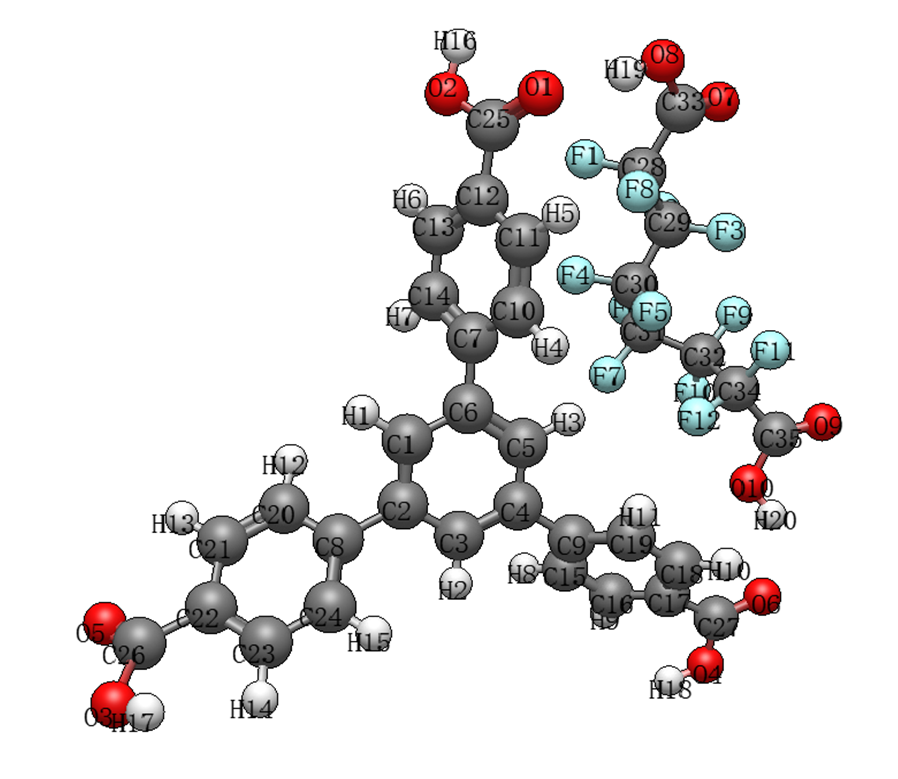


**Figure S64.** Vibration displacement calculation model of H_3_BTB with perfluorobutanesulfonic acid. Atom code: C, grey; O, red; H, white; F, celeste.

**Table**

**Table S1.** Isotropic chemical shifts measured at different temperatures for ^19^F MAS NMR experiments.

| *T* (K) | -C**F_3_** δ(iso) (ppm) | -C**F_2_**- δ(iso) (ppm) | -C**F_2_**-CF_3_ δ(iso) (ppm) |
| --- | --- | --- | --- |
| 316 | -79.5, -80.8, -82.6 | -121.4 | -126.4 |
| 306 | -79.7, -80.8, -82.7 | -121.5 | -126.5 |
| 297 | -79.3, -80.7, -82.6 | -121.5 | -126.6 |
| 273 | -79.6, -80.9, -82.8 | -121.8 | -126.8 |
| 263 | -79.6, -81.0, -82.9 | -121.8 | -127.0 |
| 253 | -79.7, -81.0, -82.8 | -121.9 | -127.0 |

**Table S2.** Vibration frequencies of H_3_BTB and PFAS.

| Target | Vibration modes | | |
| --- | --- | --- | --- |
|  | Out-of-plane (a) | Out-of-plane (b) | Out-of-plane (c) |
| H_3_BTB | 51.55 cm^-1^ | 54.59 cm^-1^ | 59.75 cm^-1^ |
| H_3_BTB with  perfluorooctanoic acid | 62.94 cm^-1^ | 60.69 cm^-1^ | 57.73 cm^-1^ |
| H_3_BTB with  tetrafluorosuccinic acid | 54.27 cm^-1^ | 66.30 cm^-1^ | 75.82 cm^-1^ |
| H_3_BTB with  heptafluorobutyric acid | 90.50 cm^-1^ | 66.94 cm^-1^ | 85.17 cm^-1^ |
| H_3_BTB with  perfluorobutanesulfonic acid | 52.86 cm^-1^ | 84.35 cm^-1^ | 68.79 cm^-1^ |
| H_3_BTB with  perfluorobutanesulfonic acid | 47.69 cm^-1^ | 71.17 cm^-1^ | 77.56 cm^-1^ |

**Table S3.** Vibration displacement results of H_3_BTB with perfluorooctanoic acid.

| Atom |  | H_3_BTB  (Å) | Vibration  displacement (Å) | H_3_BTB with  PFAS (Å) | Vibration  displacement (Å) |
| --- | --- | --- | --- | --- | --- |
| C9 | dx | 0.0164 | 0.034246 | 0.0178 | 0.04164 |
|  | dy | 0.0298 |  | 0.0116 |  |
|  | dz | -0.00397 |  | 0.0358 |  |
| C15 | dx | 0.0581 | 0.190805 | 0.0292 | 0.09134 |
|  | dy | 0.128 |  | 0.0639 |  |
|  | dz | -0.129 |  | -0.0583 |  |
| C16 | dx | 0.0552 | 0.198474 | 0.0340 | 0.1182 |
|  | dy | 0.125 |  | 0.0814 |  |
|  | dz | -0.144 |  | -0.0786 |  |
| C17 | dx | 0.0044 | 0.025176 | 0.0276 | 0.04834 |
|  | dy | 0.0147 |  | 0.0375 |  |
|  | dz | -0.0199 |  | 0.0130 |  |
| C18 | dx | -0.0355 | 0.133967 | 0.0132 | 0.1374 |
|  | dy | -0.0769 |  | -0.0238 |  |
|  | dz | 0.104 |  | 0.135 |  |
| C19 | dx | -0.0279 | 0.125946 | 0.0105 | 0.1412 |
|  | dy | -0.0638 |  | -0.0321 |  |
|  | dz | 0.105 |  | 0.137 |  |
| C27 | dx | -0.0134 | 0.024050 | 0.0462 | 0.09697 |
|  | dy | -0.0178 |  | 0.0840 |  |
|  | dz | 0.00906 |  | -0.0148 |  |
| RMS of  selected atoms | 0.1265 Å | | | 0.1032 Å | |
| RMS of  full system | 0.1400 Å | | | 0.1318 Å | |

**Table S4.** Vibration displacement results of H_3_BTB with perfluorobutanesulfonic acid.

| Atom |  | H_3_BTB  (Å) | Vibration  displacement (Å) | H_3_BTB with  PFAS (Å) | Vibration  displacement (Å) |
| --- | --- | --- | --- | --- | --- |
| C7 | dx | -0.00442 | 0.01255 | 0.0275 | 0.05792 |
|  | dy | -0.0118 |  | 0.0144 |  |
|  | dz | 0.00004 |  | -0.0489 |  |
| C10 | dx | 0.102 | 0.1777 | -0.0113 | 0.1097 |
|  | dy | 0.00136 |  | 0.0624 |  |
|  | dz | -0.146 |  | 0.0894 |  |
| C11 | dx | 0.0919 | 0.1627 | -0.00292 | 0.1273 |
|  | dy | 0.00028 |  | 0.0716 |  |
|  | dz | -0.134 |  | 0.105 |  |
| C12 | dx | -0.0114 | 0.01615 | 0.0493 | 0.06016 |
|  | dy | -0.0105 |  | 0.0292 |  |
|  | dz | 0.00447 |  | -0.0185 |  |
| C13 | dx | -0.114 | 0.1850 | 0.0915 | 0.1928 |
|  | dy | -0.0222 |  | -0.0252 |  |
|  | dz | 0.144 |  | -0.168 |  |
| C14 | dx | -0.116 | 0.1904 | 0.0788 | 0.1982 |
|  | dy | -0.0222 |  | -0.0335 |  |
|  | dz | 0.149 |  | -0.179 |  |
| C25 | dx | -0.0200 | 0.02927 | 0.0802 | 0.08900 |
|  | dy | -0.0118 |  | 0.0377 |  |
|  | dz | 0.0178 |  | -0.00837 |  |
| RMS of  selected atoms | 0.136166 Å | | | 0.130705 Å | |
| RMS of  full system | 0.140028 Å | | | 0.115336 Å | |

**References**

[1] G. M. Sheldrick, Crystal structure refinement with SHELXL. *Acta Crystallogr., Sect. C: Struct. Chem.* **2015**, *71*, 3-8.

[2] G. M. Sheldrick, A short history of SHELX. *Acta Crystallogr., Sect. A: Found. Crystallogr.* **2008**, *64*, 112-122.

[3] X. Song, T. K. Kim, H. Kim, D. Kim, S. Jeong, H. R. Moon, M. S. Lah, Post-synthetic modifications of framework metal ions in isostructural metal-organic frameworks: core-shell heterostructures via selective transmetalations. *Chem. Mater.* **2012**, *24*, 3065-3073.

[4] S. Pawsey, L. Reven, ^19^F fast magic-angle spinning NMR studies of perfluoroalkanoic acid self-assembled monolayers. *Langmuir* **2006**, *22*, 1055-1062.

[5] A. H. Karoyo, P. Sidhu, L. D. Wilson, P. Hazendonk, Characterization and dynamic properties for the solid inclusion complexes of β-cyclodextrin and perfluorooctanoic acid. *J. Phys. Chem. B* **2013**, *117*, 8269-8282.

[6] S. Hayashi, Effects of magic-angle spinning on spin-lattice relaxations in talc. *Solid State Nucl. Magn. Reson.* **1994**, *3*, 323-330.

[7] Y. Zhao, D. G. Truhlar, The M06 suite of density functionals for main group thermochemistry, thermochemical kinetics, noncovalent interactions, excited states, and transition elements: two new functionals and systematic testing of four M06-class functionals and 12 other functionals. *Theor. Chem. Acc.* **2008**, *120*, 215-241.

[8] F. Weigend, R. Ahlrichs, Balanced basis sets of split valence, triple zeta valence and quadruple zeta valence quality for H to Rn: Design and assessment of accuracy. *Phys. Chem. Chem. Phys.* **2005**, *7*, 3297-3305.

[9] F. Furche, R. Ahlrichs, C. Hättig, W. Klopper, M. Sierka, F. Weigend, Turbomole. *Wiley Interdiscip. Rev.: Comput. Mol. Sci.* **2014**, *4*, 91-100.

[10] E. R. Johnson, S. Keinan, P. Mori-Sánchez, J. Contreras-García, A. J. Cohen, W. T. Yang, Revealing noncovalent interactions. *J. Am. Chem. Soc.* **2010**, *132*, 6498-6506.

[11] Z. Liu, T. Lu, Q. Chen, Intermolecular interaction characteristics of the all-carboatomic ring, cyclo[18]carbon: Focusing on molecular adsorption and stacking. *Carbon* **2021**, *171*, 514-523.

[12] W. Humphrey, A. Dalke, K. Schulten, VMD: Visual molecular dynamics. *J. Mol. Graphics.* **1996**, *14*, 33-38.

[13] J. Mei, N. L. C. Leung, R. T. K. Kwok, J. W. Y. Lam, B. Z. Tang, Aggregation-induced emission: together we shine, united we soar! *Chem. Rev.* **2015**, *115*, 11718-11940.
